# Locus Coeruleus‐Dorsolateral Septum Projections Modulate Depression‐Like Behaviors via BDNF But Not Norepinephrine

**DOI:** 10.1002/advs.202303503

**Published:** 2023-12-28

**Authors:** Qian Zhang, You Xue, Ke Wei, Hao Wang, Yuan Ma, Yao Wei, Yi Fan, Lei Gao, Hang Yao, Fangfang Wu, Xin Ding, Qingyu Zhang, Jianhua Ding, Yi Fan, Ming Lu, Gang Hu

**Affiliations:** ^1^ Department of Pharmacology School of Medicine Nanjing University of Chinese Medicine Nanjing 210023 China; ^2^ Department of Neurology Affiliated Nanjing Brain Hospital Nanjing Medical University Nanjing 210024 China; ^3^ Jiangsu Key Laboratory of Neurodegeneration Department of Pharmacology Nanjing Medical University Nanjing 211166 China

**Keywords:** BDNF signaling, dorsolateral septum, depression‐like behavior, locus coeruleus

## Abstract

Locus coeruleus (LC) dysfunction is involved in the pathophysiology of depression; however, the neural circuits and specific molecular mechanisms responsible for this dysfunction remain unclear. Here, it is shown that activation of tyrosine hydroxylase (TH) neurons in the LC alleviates depression‐like behaviors in susceptible mice. The dorsolateral septum (dLS) is the most physiologically relevant output from the LC under stress. Stimulation of the LC^TH^‐dLS^SST^ innervation with optogenetic and chemogenetic tools bidirectionally can regulate depression‐like behaviors in both male and female mice. Mechanistically, it is found that brain‐derived neurotrophic factor (BDNF), but not norepinephrine, is required for the circuit to produce antidepressant‐like effects. Genetic overexpression of BDNF in the circuit or supplementation with BDNF protein in the dLS is sufficient to produce antidepressant‐like effects. Furthermore, viral knockdown of BDNF in this circuit abolishes the antidepressant‐like effect of ketamine, but not fluoxetine. Collectively, these findings underscore the notable antidepressant‐like role of the LC^TH^‐dLS^SST^ pathway in depression via BDNF‐TrkB signaling.

## Graphical abstract

Optogenetic and chemogenetic activation of LC^TH^ neurons after chronic social defeat stress increases the expression of BDNF, which in turn acts on SST neurons in the dLS and promotes resilience to stress. These findings highlight the notable antidepressant‐like role of the LC^TH^‐dLS^SST^ pathway in depression which can serve as prominent targets for neurostimulation.

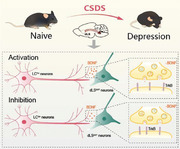

## 1 Introduction

Major depressive disorder (MDD) is a common, costly, and debilitating mental health disorder that is tied to an increased suicide risk around the world.^[^ ^1^, ^2^, ^3^, ^4^ ^]^ In spite of significant progress, most current treatments for depression are ineffective and cause intolerable side effects.^[^ ^5^, ^6^, ^7^ ^]^ A deeper understanding of depression's pathophysiology and the development of novel treatments is needed.

The locus coeruleus (LC) has been one of the most intensively studied brain regions in depression.^[^ ^8^ ^]^ The region is composed of only 3,000 brain cells ^[^ ^9^, ^10^, ^11^ ^]^ in rodents but sends numerous outputs to many CNS areas with widely divergent functions.^[^ ^12^, ^13^, ^14^, ^15^, ^16^, ^17^, ^18^ ^]^ Despite previous results from human studies showing changes in LC volume, metabolism, and receptors in depressed patients,^[^ ^8^ ^]^ how LC‐linked neural circuits participated in the pathogenesis of depression remains poorly unknown. The dorsolateral septum (dLS) is a major downstream target of the LC. Much less is known about the functional importance of the LC‐dLS connections. The dLS is a subcortical forebrain structure that receives emotional information from multiple brain regions and projects to the hypothalamus and midbrain to modulate behavioral responses.^[^ ^19^ ^]^ The dLS GABAergic neurons have been implicated in reward‐related functions such as amelioration of social deficits and drug addiction.^[^ ^19^, ^20^, ^21^, ^22^ ^]^ Recent chemogenetic and optogenetic studies revealed that activating output from the dCA3 to dLS reversed chronic social defeat stress‐induced anhedonia, social avoidance, and despair behaviors.^[^ ^23^, ^24^ ^]^ These findings suggest that the LC‐dLS circuit might be involved in the pathophysiology of depression. However, little is known about whether the LC‐dLS circuit works in depression and how the LC instructs dLS neurons to modulate depression.

For many years, LC was thought to be structurally and functionally homogeneous,^[^ ^9^, ^25^ ^]^ and the heterogeneity of the norepinephrine (NE) in LC was poorly understood. Studies have shown that brain‐derived neurotrophic factor (BDNF), dopamine, galanin, and other neuroactive substances are co‐transmitters in LC‐NE cells.^[^ ^26^, ^27^, ^28^, ^29^, ^30^ ^]^ Notably, BDNF mRNA is high in LC but very limited in dLS, while there is strong protein immunoreactivity in dLS, suggesting that it may be present in axon terminals.^[^ ^27^ ^]^ A recent study reports that BDNF‐TrkB signaling was necessary for the thalamic‐primary auditory cortex circuit to exert antidepressant‐like effects.^[^ ^31^ ^]^ More importantly, in the case of fast‐acting antidepressants such as ketamine, BDNF signaling in the hippocampus is necessary for both acute and sustained effects.^[^ ^32^, ^33^ ^]^ These studies imply that BDNF is a potential direct target for antidepressant mechanisms. However, whether BDNF in the LC‐dLS circuit regulates depressive‐like behaviors induced by chronic social defeat stress (CSDS) is still unclear.

In this study, using fiber photometry and depression‐like behavioral tasks, we observed that 10 days of chemogenetic activation of LC^TH^ neurons can rescue depression‐like behaviors in susceptible mice. Combining viral tracing, c‐Fos mapping, fiber photometry, chemogenetics, and optogenetics confirmed that the dLS^SST^ neurons are the most physiologically relevant output from the LC under stress. Using RNA‐sequence, pharmacological profiling techniques, and gain‐ or loss‐of‐function genetic approaches strongly revealed that BDNF but not norepinephrine was needed for LC^TH^‐dLS^SST^ circuit to produce antidepressant‐like effects. Further, we found that BDNF signaling in LC^TH^‐dLS circuit is necessary for ketamine to exert antidepressant‐like effects. In summary, these results were the first to suggest that the downregulation of BDNF in the LC–dLS circuit represents an endophenotype for chronic stress‐induced depression and shows the essential role of BDNF‐TrkB signaling within this circuit in conferring resilience to stress.

## 2 Results

### 2.1 Time‐Dependent Effects of Activating LC^TH^ Neurons on CSDS‐induced Depression‐Like Behaviors

**Figure 1:**
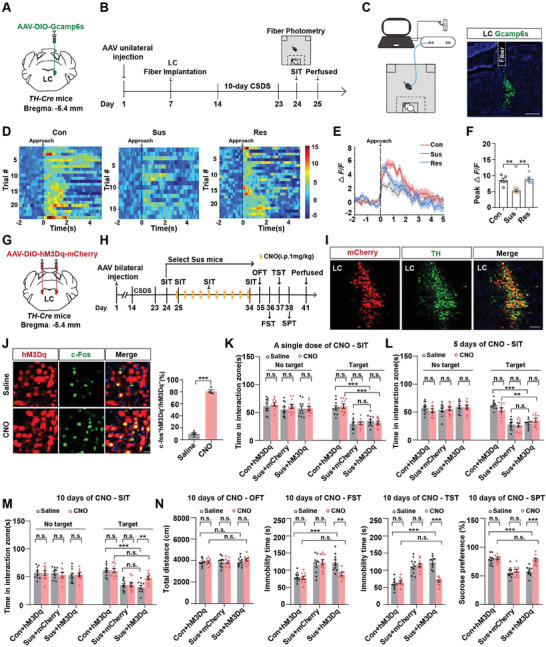
Activation of LC^TH^ neurons plays a vital role in CSDS‐induced depression‐like behaviors. A) Schematic representation of the virus injection. B) Experimental scheme of fiber photometry recordings in control and CSDS‐treated mice. C) Representative image of the LC injection sites. Scale bar = 250 µm. D) Heatmaps of Ca^2+^ transients evoked by approaching an unfamiliar CD1 mouse in control, susceptible, and resilient mice (control, trial = 24, mice = 5; susceptible, trial = 17, mice = 5; resilient, trial = 25, mice = 5). E) Average plots of Ca^2+^ responses in the control, susceptible, and resilient mice. F) Statistical analysis of the peak Ca^2+^ activity at the onset of approaching an unfamiliar CD1 mouse (*n =* 5). G) Schematic representation of virus injection. H) The experimental scheme. I) Representative images of LC injection sites. Scale bar = 100 µm. J) Representative images showing hM3Dq cells expressing c‐Fos in the saline and CNO groups (left panel). Percentage of hM3Dq‐mCherry cells expressing c‐Fos (*n =* 4). Scale bar = 50 µm. K–M) Statistical analysis of time spent in the social interaction zone in the SIT after a single dose of CNO or saline K), 5 days of repeated CNO or saline L), and 10 days of repeated CNO or saline treatment in different groups M) (*n =* 10). Con, control; Sus, susceptible; CNO, clozapine. N) Locomotor activity in the OFT, immobility time in the FST and TST, and sucrose preference in the SPT after 10 days of repeated CNO or saline stimulation in different groups (*n =* 10). Data represented as mean ± SEM. *p < 0.05, **p < 0.01, ***p < 0.001; n.s., not significant. Unpaired two‐tailed Student's t‐test was used for (J). One‐way ANOVA followed by Tukey's post hoc analysis for (F). Two‐way ANOVA followed by Bonferroni's post hoc analysis for (K–N). The statistical details can be found in Table S1, Supporting Information.

To investigate the role of the LC in depression‐like behaviors, we introduced mice to a 10‐day CSDS paradigm and segregated them into susceptible (Sus) or resilient (Res) groups according to their social interaction test (SIT) behaviors (Figure S1A,B, Supporting Information). Since the LC has been implicated in maladapted emotional states, we first examined the neuronal activity via c‐Fos expression in LC in mice subjected to CSDS. The c‐Fos signal was highly reduced in the LC^TH^ neurons in Sus mice compared to that in control and Res mice and was positively correlated with the social interaction ratio (Figure S1G–J, Supporting Information). To measure the dynamics of LC^TH^ neurons in the defeated Sus mice, we injected AAV‐DIO‐Gcamp6s into the LC of *TH‐Cre* mice and implanted optical fibers at the same site (**Figure** **1**A–C). The specificity of Cre expression in *TH‐Cre* mice was validated before use. We generated *TH‐Cre;Ai14* mice and found that robust tdTomato expression was restricted to TH neurons and was not observed in microglial cells or astrocytes (Figure S2A,B, Supporting Information). We then performed fiber photometry to record the calcium fluorescence in the mice when they interacted with a novel CD1 mouse. The amplitude of Ca^2+^ signals showed a steady decline in the Sus mice compared to that in control and Res mice (Figure 1D–F), and no signal was detected in mice expressing EGFP (Figure S3A–C, Supporting Information). Moreover, to directly investigate whether the activity of LC^TH^ neurons in Sus mice decreased gradually or suddenly during the 10‐day CSDS, we employed fiber photometry to record the activity of LC^TH^ neurons during social defeat stress. We found that the activity of LC^TH^ neurons in Sus mice gradually decreased during the 10‐day CSDS (Figure S4A,B, Supporting Information). Furthermore, we also found a selective decrease in the activity of LC^TH^ neurons in Sus mice during the onset of struggling epochs in the forced swim test (FST) and the onset of sucrose licking in the sucrose preference test (SPT) compared to the control and Res mice (Figure S5A–H, Supporting Information). Taken together, these results indicate that the activity of LC^TH^ neurons is impaired in Sus mice.

To determine whether the activation of LC^TH^ neurons produces antidepressant‐like effects, we bilaterally injected AAV‐DIO‐hM3Dq‐mCherry (or AAV‐DIO‐mCherry as control) into the LC of *TH‐Cre* mice (Figure 1G–I). The intraperitoneal injection of clozapine‐N‐oxide (CNO, 1 mg kg^−1^) activated hM3Dq‐expressing LC^TH^ neurons (Figure 1J). For acute stimulation, we administered CNO 30 min before SIT in Sus mice. Interestingly, a single dose of CNO failed to rescue social avoidance (Figure 1K). We then used chronic stimulation to investigate whether repeated activation of LC^TH^ neurons could reverse depression‐like behaviors. The avoidant behavior in Sus mice was not reversed by 5 days of chemogenetic activation but was changed by 10 days of CNO injection (Figure 1L,M). Moreover, 10‐day repeated activation of LC^TH^ neurons significantly decreased immobility time in the FST and tail suspension test (TST) and increased sucrose preference without affecting locomotor activity (Figure 1N). The control virus, hM3Dq virus, and CNO alone had no effect on depression‐like behaviors (Figure 1K–N), excluding off‐target effects.^[^ ^34^ ^]^ Additionally, the hM3Dq virus also had no impact on control mice (Figure S6A–F, Supporting Information). To rule out the possibility that non‐TH neurons in LC contribute to this process, we administered AAV‐hSyn‐DO‐hM3Dq‐EGFP to *TH‐Cre* mice. The AAV transcription can only be turned off by Cre.^[^ ^35^ ^]^ Repeated activation of non‐TH neurons in the LC failed to alleviate depression‐like behaviors induced by chronic stress (Figure S7A‐G, Supporting Information), indicating the specificity of TH neurons in mediating depression‐like behaviors. Taken together, these findings suggest that 10‐day repeated activation of LC^TH^ neurons could reverse depression‐like behaviors in the Sus mice.

### 2.2 One‐to‐One Projection Pattern of LC^TH^ Neurons to dLS, mPFC, and CeA

**Figure 2:**
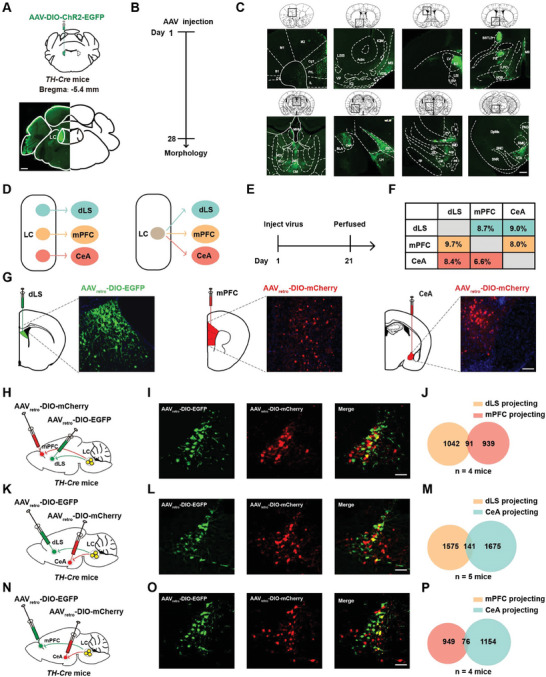
One‐to‐one projection pattern of LC^TH^ neurons to dLS, mPFC, and CeA A) Schematic representation of the viral infection in *TH‐Cre* mice. Scale bar = 600 µm. B) Schematic representation of the experimental design. C) The main distribution of LC^TH^ neuronal terminals in the brain. Scale bar = 300 µm. D1, dysgranular insular cortex; S1, primary somatosensory cortex; M1, primary motor cortex; M2, secondary motor cortex; Cg1, cingulate cortex, area 1; PrL, prelimbic cortex; IL, infralimbic cortex; LSS, lateral stripe of the striatum; Acbc, accumbens nucleus, core; VDB, nucleus of the vertical limb of the diagonal band; ICjM, islands of Calleja, major island; MS, medial septal nucleus; ICj, islands of Calleja; LV, lateral ventricle; dLS, dorsolateral septum; LSI, lateral septal nucleus, intermediate part; LSV, lateral septal nucleus, intermediate part; BSTLD, bed nucleus of the stria terminalis, lateral division, dorsal part; PS, parastrial nucleus; LPO, lateral preoptic area; HDB, nucleus of the horizontal limb of the diagonal band; MHb, medial habenular nucleus; PV, paraventricular thalamic nucleus; MD, mediodorsal thalamic nucleus; MDL, mediodorsal thalamic nucleus, lateral part; MDC, mediodorsal thalamic nucleus, central part; CM, central medial thalamic nucleus; CeA, central nucleus of the amygdala; BLA, basolateral amygdaloid nucleus, anterior part; ZID, zona incerta, dorsal part; LH, lateral hypothalamic area; fr, fasciculus retroflexus; PV, paraventricular thalamic nucleus; mL, medial lemniscus; IMD, intermediodorsal thalamic nucleus; scp, superior cerebellar peduncle (brachium conjunctivum); ZIV, zona incerta, ventral part; ZID, zona incerta, dorsal part; ns, nigrostriatal bundle; mt, mammillothalamic tract; cp, cerebral peduncle, basal part; PAG, periaqueductal gray; RMC, red nucleus, magnocellular part; DpMe, deep mesencephalic nucleus; VTA, ventral tegmental area; SNC, substantia nigra, compact part; SNR, substantia nigra, reticular part. D) Two possible projection patterns: LC^TH^ neurons independently send axons to A, B, or C (left) and LC^TH^ neurons send axons to A, B, and C (right). E) The experimental scheme. F) Overlap percentages of LC^TH^ neurons projecting to different downstream targets. G) Coronal brain slice includes a schematic (left) and the histology (right) of the AAV_retro_ injection site in the dLS, mPFC, and CeA of *TH‐Cre* mice. Scale bar = 100 µm. H) Schematic representation of AAV_retro_‐DIO‐EGFP (green) injection into the dLS, and AAV_retro_‐DIO‐mCherry (red) injection into the mPFC of *TH‐Cre* mice. I) Coronal brain slices of LC^TH^ neurons in *TH‐Cre* mice labeled with AAV_retro_‐DIO‐EGFP in green (dLS) and AAV_retro_‐DIO‐mCherry in red (mPFC). Scale bar = 100 µm. J) Venn diagram reflecting the overlap between dLS‐ and mPFC‐projecting LC^TH^ neurons (*n =* 4). K) Schematic representation of AAV_retro_‐DIO‐EGFP injection into the dLS and AAV_retro_‐DIO‐mCherry injection into the CeA of *TH‐Cre* mice. L) Coronal brain slices of LC^TH^ neurons in *TH‐Cre* mice labeled with AAV_retro_‐DIO‐EGFP in green (dLS) and AAV_retro_‐DIO‐mCherry in red (CeA). Scale bar = 100 µm. M) Venn diagram showing the overlap between dLS‐ and CeA‐projecting LC^TH^ neurons (*n =* 5). N) Schematic representation of AAV_retro_‐DIO‐EGFP injection into the mPFC and AAV_retro_‐DIO‐mCherry injection into the CeA of *TH‐Cre* mice. O) Coronal brain slices of LC^TH^ neurons from *TH‐Cre* mice labeled with AAV_retro_‐DIO‐EGFP in green (mPFC) and AAV_retro_‐DIO‐mCherry in red (CeA). Scale bar = 100 µm. P) Venn diagram showing the overlap between mPFC‐ and CeA‐projecting LC^TH^ neurons (*n =* 4 mice). The statistical details can be found in Table S1, Supporting Information.

Given that repeated activation of LC^TH^ neurons can alleviate depression‐like behaviors, we next investigated the neural circuits underlying the role of the LC in depression. We injected AAV‐DIO‐ChR2‐EGFP into *TH‐Cre* mice to label downstream brain regions (**Figure** **2**A,B). We observed intense EGFP expression in the dLS, medial prefrontal cortex (mPFC), central nucleus of the amygdala (CeA), medial septal nucleus (MS), zona incerta (ZI), ventral tegmental area (VTA), and other regions (Figure 2C). To detect neuronal activity in the downstream regions of the LC associated with social stress, we examined the expression of c‐Fos when the animals were re‐exposed to a new CD1 mouse. We found that after SIT, the number of c‐Fos positive neurons was significantly decreased in the dLS in Sus mice compared to control and Res groups (Figure S8A,B, Supporting Information). Since the CeA and mPFC serve as crucial downstream outputs of the LC, we want to investigate whether dLS, CeA, and mPFC are involved in processing stress information from the LC.

To further identify the neural circuits of the LC^TH^‐dLS, LC^TH^‐mPFC, and LC^TH^‐CeA, retrograde tracers AAV_retro_‐DIO‐EGFP and AAV_retro_‐DIO‐mCherry were injected into the dLS, mPFC, and CeA of *TH‐Cre* mice to track each target region (Figure 2D,E,G). Interestingly, only 8.7% of dLS‐projecting LC^TH^ neurons projected to the mPFC, whereas 9.7% of mPFC‐projecting LC^TH^ neurons projected to the dLS (Figure 2F,H–J). Additionally, 9.0% of the dLS‐projecting LC^TH^ neurons sent axon projections to the CeA, whereas 8.4% of the CeA‐projecting LC^TH^ neurons sent axon projections to the dLS (Figure 2F,K–M). Furthermore, 8.0% of the mPFC‐projecting LC^TH^ neurons projected to the CeA, and 6.6% of the CeA‐projecting LC^TH^ neurons projected to the mPFC (Figure 2F,N–P). These data suggest a minimal overlap between LC^TH^ neurons projecting to the dLS, mPFC, and CeA, indicating a one‐to‐one projection pattern for LC^TH^ neurons targeting these regions.

### 2.3 Activating/Silencing the LC^TH^‐dLS Circuit Oppositely Modulates Mouse Depression‐Like Behaviors

**Figure 3:**
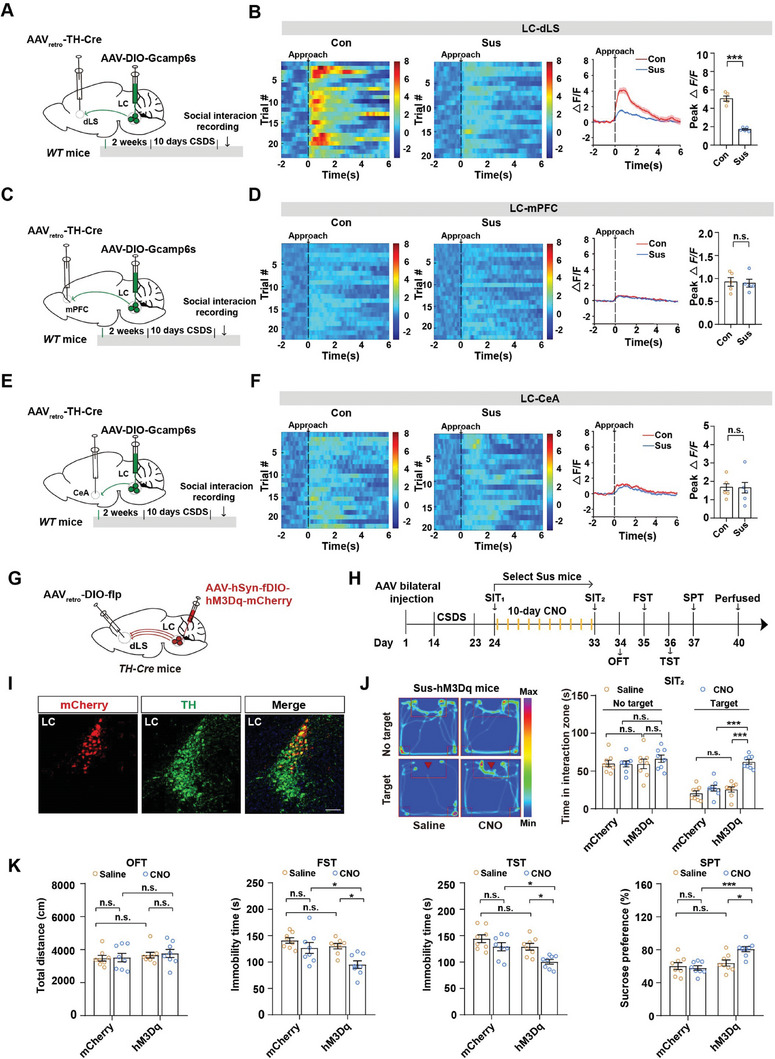
Activating LC^TH^‐dLS circuit promotes resilience to CSDS‐induced depression‐like behaviors. A) The experimental strategy for recording the activity of the LC^TH^‐dLS circuit in the SIT using fiber photometry. B) Heatmaps of Ca^2+^ transients (left), average plots of Ca^2+^ response (middle), and peak Ca^2+^ activities (right) evoked by approaching an unfamiliar CD1 mouse in the LC^TH^‐dLS circuit in control and susceptible mice (Con, trial = 23, mice = 5; susceptible, trial = 20, mice = 5). C) The experimental strategy for recording the activity of the LC^TH^‐mPFC circuit in the SIT using fiber photometry. D) Heatmaps of Ca^2+^ transients (left), average plots of Ca^2+^ response (middle), and peak Ca^2+^ activities (right) evoked by approaching an unfamiliar CD1 mouse in the LC^TH^‐mPFC circuit in control and susceptible mice (Con, trial = 22, mice = 5; susceptible, trial = 22, mice = 5). E) The experimental strategy for recording the activity of the LC^TH^‐CeA circuit in the SIT using fiber photometry. F) Heatmaps of Ca^2+^ transients (left), average plots of Ca^2+^ response (middle), and peak Ca^2+^ activities (right) evoked by approaching an unfamiliar CD1 mouse in the LC^TH^‐CeA circuit in control and susceptible mice (Con, trial = 22, mice = 5; susceptible, trial = 20, mice = 5). G) Schematic representation of virus injection. H) Experimental scheme. I) Representative images of LC injection sites. Scale bar = 100 µm. J) Representative social interaction tracks and time spent in the interaction zone in the SIT by different groups (*n =* 8) Sus, susceptible. K) Locomotor activity in the OFT, immobility time in the FST and TST, and sucrose preference in the SPT of susceptible mice expressing the mCherry or hM3Dq virus (*n =* 8). Data represented as mean ± SEM. *p < 0.05, **p < 0.01, ***p < 0.001; n.s., not significant. Unpaired two‐tailed Student's t‐test was used for (B,D,F). Two‐way ANOVA followed by Bonferroni's post hoc analysis for J, K). The statistical details can be found in Table S1, Supporting Information.

To further compare the functions of the three circuits in depression, we performed in vivo calcium recordings to directly visualize whether these outputs respond promptly to social stress. We performed an intersectional viral approach, and a retrograde virus (AAV_retro_‐TH‐Cre) expressing TH‐Cre‐recombinase was stereotaxically injected into the dLS, mPFC, or CeA, while AAV‐DIO‐Gcamp6s was injected into the LC and optic fibers above the LC to collect circuit calcium signals from the neuronal soma in the LC (**Figure** **3**A,C,E). We observed that dLS‐projecting LC^TH^ neurons responded vigorously to social interactions in control mice and that this response was heavily impaired in Sus mice (Figure 3B). However, calcium signals were barely detectable in the other two groups, unlike in the LC^TH^‐dLS circuit (Figure 3C–F). These results suggest that although multiple downstream regions were inhibited during the stressor in the c‐Fos experiment, they seldom responded to social avoidance when recording calcium signals, except for the dLS. Collectively, these observations underscore the vital role of the LC^TH^‐dLS pathway in social avoidance induced by chronic social stress.

Considering that the LC^TH^‐dLS pathway responds to stress, we aimed to determine whether activation of the LC^TH^‐dLS pathway promotes resilience to stress. To achieve this, we used a dual‐virus strategy by bilaterally injecting retrogradely transported and Cre recombinase–dependent AAV_retro_‐DIO‐flp into the dLS and flip‐dependent AAV‐fDIO‐hM3Dq‐mCherry into the LC of *TH‐Cre* mice (Figure 3G,H). This strategy allows selective activation of dLS‐projecting LC^TH^ neurons (Figure 3I). We found that after repeated activation of the LC^TH^‐dLS circuit, hM3Dq‐CNO mice showed improved social interaction compared with hM3Dq‐Saline mice. Additionally, they displayed decreased immobility in the FST and TST, as well as increased sucrose preference in the SPT, without affecting the distance traveled in the open field test (OFT) (Figure 3J,K). Moreover, many studies often omit female subjects despite evidence of higher rates of depression in women. Thus, we used a novel method for CSDS in female mice ^[^ ^36^ ^]^ and found that activation of the LC^TH^‐dLS circuit in female mice mimicked the antidepressant‐like effect found in male mice (Figure S9A–D, Supporting Information). As expected, repeated activation of the LC^TH^‐mPFC circuit (Figure S10A–D, Supporting Information) or the LC^TH^‐CeA circuit (Figure S10E–H, Supporting Information) had no significant effect on depression‐like phenotypes in Sus mice.

Next, we examined whether the reduced activity of the LC^TH^‐dLS circuit could lead to depression‐like behaviors in a subthreshold social defeat stress (SSDS) paradigm. Previous studies have shown three days of SSDS has no behavioral effects on wild‐type mice. Only mice vulnerable to acute stress develop depressive behaviors following SSDS.^[^ ^37^, ^38^ ^]^ The Sus mice were intraperitoneally administered CNO 30 min before each SSDS. We found that the hM4Di‐CNO group exhibited apparent depression‐like behaviors compared to hM4Di‐Saline and mCherry‐CNO groups. These results suggest that inhibition of LC^TH^‐dLS circuit can render mice more susceptible to SSDS and lead to depression‐like behaviors (Figure S11A–E, Supporting Information). Taken together, these data indicate that the LC^TH^‐dLS circuit is not only necessary but also sufficient to render mice vulnerable to stress.

### 2.4 The Somatostatin Subtype of GABAergic Neurons in the dLS is the Downstream Effector of the Antidepressant LC^TH^ Neuron Circuit

**Figure 4:**
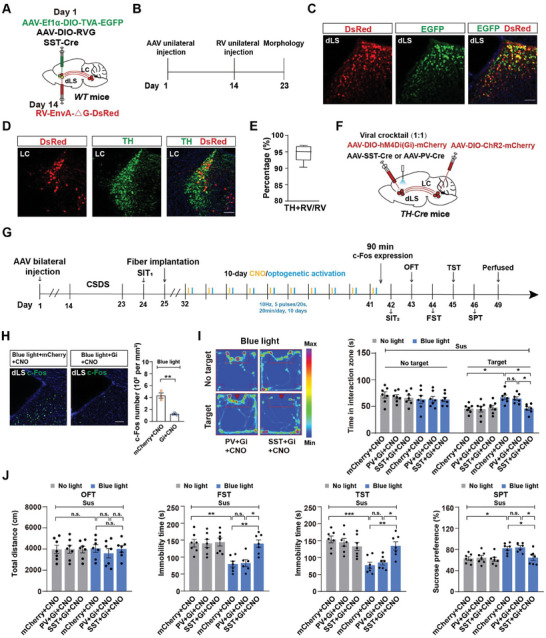
dLS^SST^ neurons but not PV neurons are involved in the antidepressant‐like activity of LC^TH^‐dLS circuit. A) Schematic representation of the Cre‐dependent retrograde monosynaptic rabies tracing strategy. B) Experimental scheme for retrograde tracing. C) Representative images showing DsRed/EGFP double‐labeled starter cells in the dLS of mice injected with the SST‐Cre virus. Scale bar = 100 µm. D) DsRed signals traced from dLS SST neurons co‐localized with TH immunofluorescence in the LC. Scale bar = 100 µm. E) The percentage of DsRed‐labeled neurons expressing TH in the LC (*n =* 6). F) Schematic of the injection strategy. AAV‐DIO‐ChR2‐mCherry was injected into the LC, and a viral cocktail (1:1) of AAV‐PV‐Cre or AAV‐SST‐Cre and AAV‐DIO‐hM4Di‐mCherry were injected into the dLS of *TH‐Cre* mice. G) Timeline of the experiments. H) Representative images showing mCherry cells and Gi cells expressing c‐Fos in dLS SST neurons induced by photostimulation of LC^TH^‐dLS terminal fibers (left). Percentage of total mCherry cells and Gi cells expressing c‐Fos in the mCherry‐CNO and Gi‐CNO groups (right) (*n =* 3). I) Representative social interaction tracks of PV‐Gi‐CNO and SST‐Gi‐CNO following 10‐day repeated stimulation with CNO and blue light in susceptible mice (left). Time spent in the interaction zone in the SIT in different groups (right) (*n =* 7). The group of “No light+ mCherry + CNO” and “Blue light + mCherry + CNO” consisted of a mixture of viruses AAV‐DIO‐mCherry, AAV‐PV‐Cre and AAV‐SST‐Cre, serving as the control. J) Locomotor activity in the OFT, immobility time in the FST and TST, and sucrose preference in the SPT in susceptible mice following the inhibition of SST/PV neurons in the dLS (*n =* 7). Data represented as mean ± SEM. *p < 0.05, **p < 0.01, ***p < 0.001; n.s., not significant. Unpaired two‐tailed Student's t‐test for (H). Two‐way ANOVA followed by Bonferroni's post hoc analysis for (I,J). The statistical details can be found in Table S1, Supporting Information.

These results led us to investigate how activation of LC^TH^ neurons modulates local dLS neurons. The dLS consists of inhibitory neurons,^[^ ^39^ ^]^ including abundant somatostatin (SST) subtype neurons and a small number of parvalbumin (PV) neurons. To map whether PV or SST neurons, were differentially targeted by LC^TH^ neuron input, a monosynaptic retrograde‐modified rabies virus tracing technique was used. Cre‐dependent helper viruses (AAV‐DIO‐TVA‐EGFP and AAV‐DIO‐RVG) and either AAV‐SST‐Cre or AAV‐PV‐Cre were injected into the dLS. After 2 weeks, RV‐EnvA‐ΔG‐DsRed was injected into the same coordinates (**Figure** **4**A,B and Figure S12A–C, Supporting Information). DsRed/EGFP co‐localization cells were observed in dLS, and red fluorescent RV‐DsRed‐infected cells were observed in LC (Figure 4C,D and Figure S12D,E, Supporting Information). We found a large population of RV‐DsRed cells (94.53% ± 1.085) that were TH‐positive among SST neurons, while a fraction of RV‐DsRed^+^/TH^+^ signal (22.22% ± 2.833) was among PV neurons (Figure 4E and Figure S12F, Supporting Information).

To investigate the roles of dLS SST or PV neurons in depression‐like behaviors, we injected a viral cocktail (1:1) containing AAV‐PV‐Cre or AAV‐SST‐Cre along with hM4Di‐mCherry into the dLS to selectively inactivate PV or SST neurons (Figure S13A–C, Supporting Information). After 3 weeks of virus expression, the CNO or Saline was intraperitoneally injection for 10 days. We found that direct inhibition of dLS SST or PV neurons alone cannot induce depression‐like behaviors (Figure S13D,E, Supporting Information). To further investigate whether SST or PV neurons are required for the antidepressant‐like effect of the LC^TH^‐dLS circuit, we targeted ChR2 to LC^TH^ neurons by injecting AAV‐DIO‐ChR2‐mCherry into the LC of *TH‐Cre* mice, and a viral cocktail (1:1) of AAV‐PV‐Cre or AAV‐SST‐Cre and hM4Di‐mCherry into the dLS for the selective inactivation of PV or SST neurons (Figure 4F,G). We found that inhibition of SST neurons in the dLS by treatment with CNO markedly decreased the expression of c‐Fos induced by photostimulation (473 nm, 10 Hz, 5 pulses/20 s, 20 min per day) of the LC^TH^‐dLS circuit (Figure 4H). We inhibited dLS SST or PV cells through intraperitoneal injection of CNO (1 mg kg^−1^) 30 min prior to optogenetic activation and repeated this procedure for 10 consecutive days. Importantly, we found that chemogenetic inhibition of dLS SST neurons, rather than PV neurons, abrogated the antidepressant‐like effects of LC^TH^‐dLS terminal stimulation on the Sus mice (Figure 4I,J). Collectively, these results indicate the dLS SST neurons are the downstream effectors of LC^TH^‐dLS circuit in antidepressant‐like effects.

### 2.5 BDNF but not NE in LC^TH^‐dLS Circuit Mediates Stress Susceptibility

As the LC is a major source of NE, we wanted to verify whether NE mediates the effects of the LC^TH^‐dLS circuit on depression‐linking. We injected a G protein‐coupled receptor activation‐based NE sensor (GRAB_NE_ sensor, NE2m) into the dLS and implanted optical fibers at the same site (Figure S14A, Supporting Information). Interestingly, we did not detect NE release in response to SIT in the control and Sus mice (Figure S14B, Supporting Information). To further confirm the role of NE in LC^TH^‐dLS circuit, we injected AAV‐DIO‐hM3Dq‐mCherry into LC and implanted cannulae into dLS of the Sus mice and treated them with a non‐selective α‐adrenoceptor antagonist (phentolamine, 2.8 µg/side) and a non‐selective β‐adrenergic receptor antagonist (propranolol, 1.3 µg/side) 30 min before injecting CNO (1 µg/side) (Figure S14C,G, Supporting Information). We found that both the NE α‐adrenergic receptor antagonist and β‐adrenergic receptor antagonist did not disrupt the anti‐depressive effects produced by the activation of the circuit (Figure S14C–J, Supporting Information). These results suggest that NE in the LC^TH^‐dLS circuit is not required for antidepressant‐like effects.

**Figure 5:**
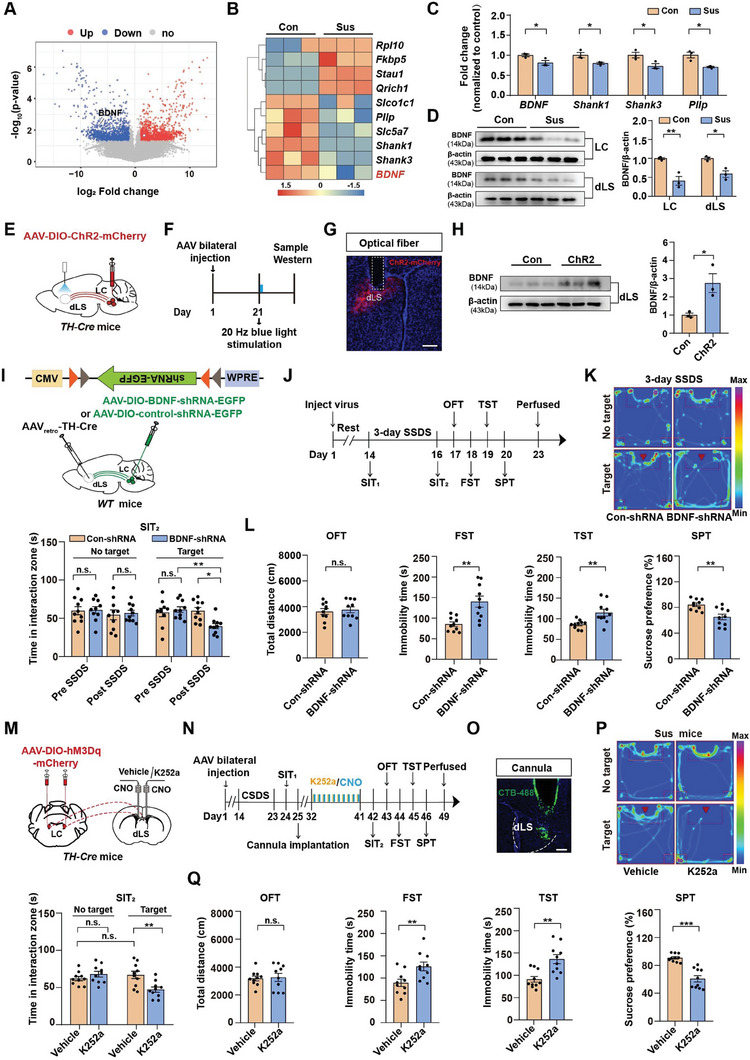
LC^TH^‐dLS circuit modulation of depression‐like behavior is BDNF signaling dependent. A) Volcano plot showing differentially regulated genes in LC of control and susceptible mice (*n =* 3). B) Heatmap of genes related to psychiatric disorders as determined by RNA sequencing of control and susceptible mice (*n =* 3). C) Quantitative real‐time PCR (qRT‐PCR) verification of specific target genes (B) (*n =* 3). D) Western blot analysis of BDNF expression in the LC and dLS (*n =* 3). E) Schematic representation of injection strategy. AAV‐DIO‐ChR2‐mCherry was injected into the LC of *TH‐Cre* mice, and optical fibers were implanted above the dLS. F) Timeline of the experiments. G) Representative image of dLS injection sites. Scale bar = 200 µm. H) Western blot analysis of BDNF expression in the dLS of Control and ChR2 mice (*n =* 3). I) Schematic representation of virus injection. J) Experimental scheme. K) Representative social interaction tracks (left). Time in the interaction zone in the SIT between Con‐shRNA and BDNF‐shRNA mice before and after 3 days of SSDS (right) (*n =* 10). L) Locomotor activity and depression‐like behaviors in con‐shRNA and BDNF‐shRNA mice (*n =* 10). M) Schematic of injection strategy. AAV‐DIO‐hM3Dq‐mCherry was injected into the LC and the CNO was injected 30 min prior to the administration of K252a in the dLS. N) Timeline of experiments O) Infusion sites were verified using 150 nL CTB‐488 in dLS. Scale bar = 200 µm. P) Representative social interaction tracks of mice infused with vehicle or K252a, and the time spent in the interaction zone in the SIT in different groups in *TH‐Cre* mice (*n =* 10). Q) Locomotor activity and depression‐like behaviors in susceptible *TH‐Cre* mice infused with vehicle or K252a (*n =* 10). Data represented as mean ± SEM. *p < 0.05, **p < 0.01, ***p < 0.001; n.s., not significant. Unpaired two‐tailed Student's t‐tests for (H), (L), and (Q). One‐way ANOVA followed by Tukey's post hoc analysis for (C,D). Two‐way ANOVA followed by Bonferroni's post hoc analysis for (K) and (P). The statistical details can be found in Table S1, Supporting Information.

To further understand the underlying mechanisms mediating decreased activity in the LC^TH^‐dLS circuit of Sus mice, we used RNA sequencing (RNA‐seq) of the LC tissue to identify candidate genes. RNA‐seq analysis of LC tissues from the defeated Sus mice showed that 1141 genes were significantly upregulated and 1331 genes were downregulated (**Figure** **5**A). Among these genes, those related to psychiatric disorders were notably altered in the defeated Sus mice compared to the controls (Figure 5B,C). *BDNF*, the gene of interest in this study, is a known neuromodulator that plays a vital role in depression and binds specifically to TrkB receptors at synaptic terminals.^[^ ^40^ ^]^ We found that the BDNF protein levels in the LC and dLS were lower in Sus mice than in control mice (Figure 5D). To test this hypothesis, we injected AAV‐DIO‐ChR2‐mCherry into the LC of *TH‐Cre* mice and activated the LC^TH^‐dLS circuit using blue light (473 nm) for 20 min. We found that optogenetic activation of the LC^TH^‐dLS circuit increased BDNF protein levels in dLS (Figure 5E–H). Next, we detected the mRNA levels in the LC^TH^‐dLS circuit using fluorescence‐activated cell sorting (FACS) after 10 days of CSDS and observed that the mRNA levels of BDNF were significantly decreased in this circuit in Sus mice compared to control mice (Figure S15A–D, Supporting Information). These results suggest that reduced BDNF transport from LC to dLS may be responsible for depression‐like behaviors.

To investigate whether BDNF signaling within LC^TH^‐dLS is required for stress susceptibility, we first investigated the role of BDNF in LC^TH^ neurons. The BDNF in LC^TH^ neurons was specifically knocked out by injecting virus‐packaged Cre recombinase fused with EGFP or EGFP under the control of the TH promoter into the LC of *BDNF^flox/+^* mice (Figure S16A,B, Supporting Information). The efficiency of the knockout was confirmed by immunostaining and western blot analysis (Figure S16C,D, Supporting Information). Intriguingly, we found that knockout BDNF in the LC^TH^ neurons had little effect on SIT in naïve mice (Figure S16E,F, Supporting Information). However, after 3 days of SSDS, the deletion of BDNF in LC^TH^ neurons induced depression‐like behavior (Figure S16F,G, Supporting Information). Next, we examined whether BDNF signaling within the LC^TH^‐dLS circuit is necessary for stress vulnerability. After validating the efficiency of BDNF‐shRNA (Figure S17A,B, Supporting Information), we deleted BDNF in the LC^TH^‐dLS circuit using an intersectional viral approach, in which a retrograde virus (AAV_retro_‐TH‐Cre) expressing TH‐Cre‐recombinase was stereotaxically injected into the dLS, and an AAV‐DIO‐BDNF‐shRNA‐EGFP (BDNF‐shRNA) or scramble virus (Con‐shRNA) was injected in LC^TH^ neurons (Figure 5I). Then all the mice were subjected to a 3‐day SSDS experiment (Figure 5J). Consistently, we found that after 3 days of the SSDS paradigm, knockdown of BDNF in the LC^TH^‐dLS circuit increased depressive‐like phenotypes, including increased social avoidance and immobility time in the FST and TST, and reduced sucrose preference in the SPT compared with control mice (Figure 5K,L). These results confirmed the expression of BDNF within the LC^TH^‐dLS circuit, which underlies its contribution to depression‐like behaviors induced by CSDS.

To investigate the functional characteristics of the BDNF in the LC^TH^‐dLS circuit, we utilized a combination of optogenetic stimulation and multi‐region electrophysiological recordings. This approach enabled us to analyze the response of dLS neuronal activity to LC^TH^‐dLS projections as well as the effects of TrkB receptor antagonists (K252a) administration. We observed that most TrkB‐positive cells co‐expressed with SST‐positive neurons (Figure S18A,B, Supporting Information). Moreover, we found activation of LC^TH^‐dLS (10 Hz, 473 nm, 5 ms) elicited excitation in dLS neurons, while administration of K252a reduced the activity of dLS neurons, implying a functional role for BDNF released from the LC to the dLS (Figure S18B–G, Supporting Information). To further confirm the function of BDNF in the LC^TH^‐dLS circuit, we injected the AAV‐DIO‐hM3Dq‐mCherry into the *TH‐Cre* mice and implanted cannulae into dLS of the Sus mice. Then we treated the *TH‐Cre* mice with K252a 30 min prior to injecting CNO (3 µM) (Figure 5M–O). We found that K252a disrupted the antidepressant‐like effects produced by the activation of the circuit (Figure 5P,Q). Thus, BDNF within the LC^TH^‐dLS circuit plays a crucial role in the antidepressant‐like effect. To rule out the influence of inhibiting TrkB receptors on depression‐like behaviors, we administered K252a to directly inhibit TrkB receptors in wild‐type mice (Figure S19A,B, Supporting Information). We found inhibition of TrkB receptors could not induce depression‐like behaviors (Figure S19C,D, Supporting Information). Since K252a is not a specific TrkB inhibitor, we want to test the effects of intra‐dLS infusion of anti‐BDNF neutralizing antibody (BDNF‐nAb) on the antidepressant‐like effects produced by chemogenetic activation of LC^TH^ neurons in *TH‐Cre* mice (Figure S20A,B, Supporting Information). We found that BDNF‐nAb disrupted antidepressant‐like effects produced by activation of the circuit, which was also consistent with our K252a results (Figure S20C,D, Supporting Information). Taken together, these results suggest that BDNF (but not NE) signaling in the LC^TH^‐dLS circuit is necessary for the antidepressant‐like effects.

### 2.6 Supplementation of BDNF in the LC^TH^‐dLS Circuit Ameliorates Depression‐Like Behaviors

**Figure 6:**
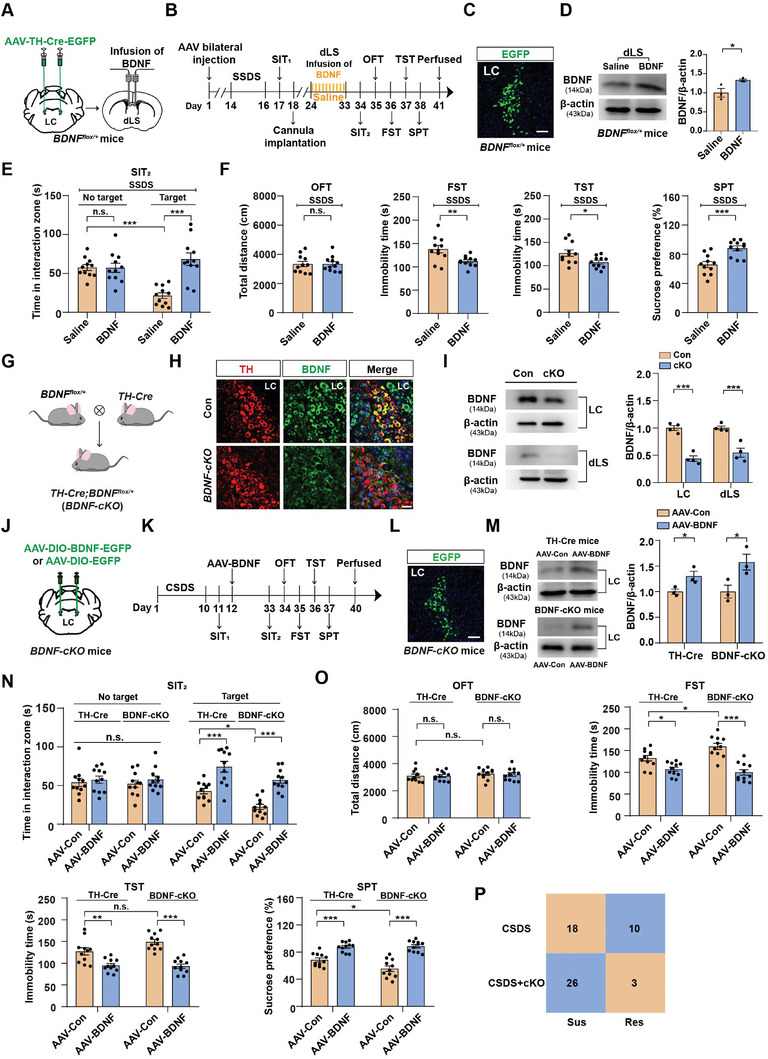
Supplementation of BDNF in the LC^TH^‐dLS circuit produces significant antidepressant‐like effects. A) Schematic representation of the viral infection in *BDNF^flox/+^* mice. B) Timeline of experiments C) Representative images of AAV‐TH‐Cre‐EGFP expression in the LC of *BDNF^flox/+^* mice. Scale bars = 100 µm. D) Western blot analysis of the BDNF protein in the dLS of *BDNF^flox/+^* mice treated with Saline or BDNF (*n =* 3). E) The time spent in the interaction zone in the SIT in different groups (*n =* 11) F) Locomotor activity and depression‐like behaviors in *BDNF^flox/+^* mice after 3 days of SSDS infusion with saline or BDNF (*n =* 11). G) Generation of *BDNF‐cKO* mice. H) Double‐immunofluorescence staining for BDNF (green) and TH (red) in *BDNF‐cKO* and control mice. Scale bar = 50 µm. I) Western blot analysis of the BDNF protein in the LC and dLS of *BDNF‐cKO* and control mice (*n =* 4). J) Schematic of injection strategy. AAV‐Ef1a‐DIO‐EGFP‐BDNF‐3FLAG was bilaterally injected into the LC of *BDNF‐cKO* and *TH‐Cre* mice. K) Timeline of the experiments. L) Representative images of AAV‐Ef1a‐DIO‐EGFP‐BDNF‐3FLAG expression in LC. Scale bars = 100 µm. M) Western blot analysis of the BDNF protein in the LC of *TH‐Cre* mice and *BDNF‐cKO* mice treated with the virus of AAV‐Con and AAV‐BDNF (*n =* 3). N) The time spent in the interaction zone in the SIT in different groups (*n =* 11). O) Locomotor activity and depression‐like behaviors in *BDNF‐cKO* and *TH‐Cre* mice expressing either AAV‐Con or AAV‐BDNF in the LC (*n =* 11). P) The 2 × 2 contingency table shows the correlation between *BDNF‐cKO* and social avoidance. Numbers in the box indicate the number of animals. Data represented as mean ± SEM. *p < 0.05, **p < 0.01, ***p < 0.001; n.s., not significant. Unpaired two‐tailed Student's t‐tests for (D) and (F). Data were analyzed by one‐way ANOVA followed by Fisher's exact test for panels (P). One‐way ANOVA followed by Tukey's post hoc analysis for (I) and (M). Two‐way ANOVA followed by Bonferroni's post hoc analysis for (E) and (N,O). The statistical details can be found in Table S1, Supporting Information.

To further examine the function of BDNF, we used a pharmacological approach to determine whether restoration of BDNF in this circuit would be sufficient to reverse depression‐like behaviors. We generated a mouse model with neuron‐specific BDNF knockout in LC^TH^ neurons by injecting AAV‐TH‐Cre‐EGFP or AAV‐TH‐EGFP. All *BDNF^flox/+^* mice were subjected to SSDS for 3 days. Next, BDNF protein or saline was infused bilaterally into the dLS for 10 days (**Figure** **6**A–C). We found that BDNF protein infusion into the dLS markedly raised the expression of BDNF in the dLS compared to the group treated with saline (Figure 6D). Moreover, we observed that BDNF gene knockout in LC^TH^ neurons and saline injection in the dLS resulted in depression‐like behaviors (Figure 6E,F). However, BDNF infusion into the dLS prevented this effect, displayed decreased social avoidance and immobility time in the FST and TST, and increased sucrose preference in the SPT (Figure 6E,F). Furthermore, since a single local infusion of BDNF into the mPFC is sufficient to produce antidepressant‐like effects,^[^ ^40^, ^41^, ^42^ ^]^ we want to test whether single local infusion of BDNF into dLS could also produce antidepressant‐like effects. At the behavioral level, we found that BDNF gene knockout in the LC^TH^ neurons and now with single local infusion of BDNF into dLS also displayed increased social interaction time and decreased immobility in the FST and TST, and improved sucrose preference in SPT, without affecting the distance traveled in the OFT (Figure S21A–D, Supporting Information). Therefore, BDNF supplementation in this circuit rescued the stress vulnerability caused by the BDNF gene knockout in the LC^TH^ neurons, supporting the hypothesis that the LC^TH^‐dLS circuit regulates depression‐like behaviors through BDNF signaling.

We then tested whether activation of residual BDNF could reverse depression‐like symptoms. We generated a TH‐specific BDNF deletion mouse line, *TH‐Cre*;*BDNF^flox/+^* (conditional knockout [*BDNF‐cKO*]), for the behavioral studies (Figure 6G and Figure S22A, Supporting Information). *BDNF‐cKO* and littermate mice showed normal growth rates, body weights, brain structures, and equal densities of TH neurons (Figure S22B,D, Supporting Information). Immunostaining and western blotting validated the efficiency of *BDNF‐cKO* mice (Figure 6H,I). We subjected *BDNF‐cKO* and control mice (*TH‐Cre* mice) to CSDS and then bilaterally infused AAV‐DIO‐BDNF‐EGFP or AAV‐DIO‐EGFP into the LC (Figure 6J–L). The efficiency of the AAV‐BDNF was confirmed by western blot analysis (Figure 6M). Behavioral tests indicated that mice injected with AAV‐BDNF exhibited significant relief from depression‐like phenotypes, including reduced immobility in the FST and TST and improved social interaction time and sucrose preference in *BDNF‐cKO* mice and *TH‐Cre* mice (Figure 6N,O). Particularly, the behavioral tests showed that stressed *BDNF‐cKO* mice exhibited a higher immobile time in the FST and TST and a lower social interaction and sucrose preference than stressed *TH‐Cre* mice (Figure 6N,O). Moreover, we found that a higher percentage of *BDNF‐cKO* mice were susceptible to CSDS (89.7% *BDNF‐cKO* vs. 64.3% control) (Figure 6P), suggesting that a lack of BDNF renders animals more vulnerable to chronic social defeat stress, and infusion of BDNF in the LC markedly alleviated this phenomenon. Collectively, these results demonstrate that enhancing BDNF expression within the LC or the dLS effectively and sufficiently rescues depression‐like behaviors in the Sus mice.

### 2.7 BDNF in the LC^TH^‐dLS Circuit is Necessary for the Sustained Antidepressant‐Like Effects Of Ketamine But Not Fluoxetine

**Figure 7:**
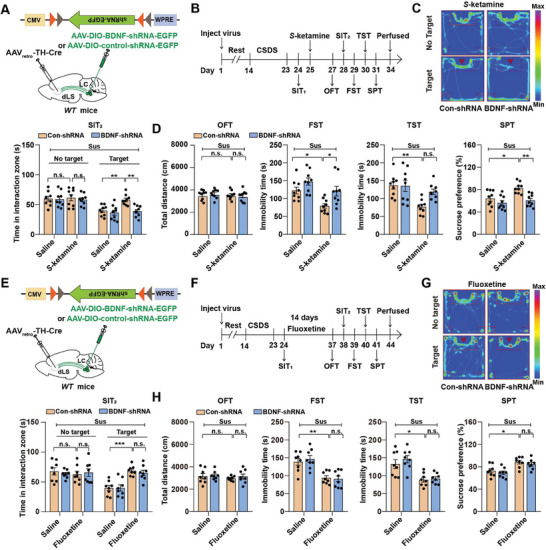
BDNF was necessary for the LC^TH^‐dLS circuit to sustain the antidepressant‐like effects of *S*‐ketamine but not fluoxetine. A) Schematic representation of the Cre‐dependent AAV expressing BDNF‐shRNA‐EGFP in the LC^TH^‐dLS circuit. B) Timeline of experiments. C) Representative social interaction tracks of BDNF‐shRNA and Con‐shRNA mice and the time spent in the interaction zone in the SIT in different groups (*n =* 9). D) Locomotor activity and depression‐like behaviors in BDNF‐shRNA and control‐shRNA mice injected with saline or *S*‐ketamine (*n =* 9). E) Schematic representation of virus injection. F) Timeline of the experiments. G) Representative social interaction tracks of BDNF‐shRNA and Con‐shRNA mice and the time spent in the interaction zone in the SIT in different groups (*n =* 8). H) Locomotor activity and depression‐like behaviors in BDNF‐shRNA and Con‐shRNA mice injected with saline or fluoxetine (*n =* 8). Data represented as mean ± SEM. *p < 0.05, **p < 0.01, ***p < 0.001; n.s., not significant. Two‐way ANOVA followed by Bonferroni's post hoc analysis for (C,D) and (G,H). The statistical details can be found in Table S1, Supporting Information.

Ketamine, a non‐competitive antagonist of N‐methyl‐D‐aspartate (NMDA) receptors, induces rapid‐acting and sustained antidepressant effects in patients with treatment‐resistant depression.^[^ ^43^ ^]^ A single subanesthetic dose of ketamine exhibits a long‐term antidepressant‐like effect that persists for days or weeks, suggesting that other mechanisms, in addition to direct pharmacological effects, may be involved in its sustained antidepressant effects. To test whether BDNF in the LC^TH^‐dLS circuit was required for the sustained antidepressant‐like effects of ketamine, we deleted BDNF in the circuit by injecting AAV_retro_‐TH‐Cre into the dLS and BDNF‐shRNA or Con‐shRNA into the LC (**Figure** **7**A). Next, all mice were subjected to CSDS, and then we intraperitoneally injected a single 10 mg kg^−1^ dose of ketamine (*S*‐ketamine) or saline into the Sus mice (Figure 7B). The antidepressant‐like effects of *S*‐ketamine were evaluated 2 days after injection. We found that *S*‐ketamine reversed the behavioral deficits in mice injected with Con‐shRNA in the SIT, FST, TST, and SPT, whereas the antidepressant‐like effects of *S*‐ketamine were not detected in mice with a BDNF deletion in the LC^TH^‐dLS circuit (Figure 7C,D). Additionally, we evaluated locomotor activity to exclude any confounding effects of hyperlocomotion on behavioral phenotypes after *S*‐ketamine treatment and found no apparent differences in total activity among the groups (Figure 7D). Since *R*‐ketamine produces more potent, long‐lasting, and safer antidepressant‐like effects than *S*‐ketamine,^[^ ^44^, ^45^ ^]^ we examined whether the antidepressant‐like effects of *R*‐ketamine were also blocked by BDNF knockdown in the LC^TH^‐dLS pathway and intra‐dLS infusion of BDNF‐neutralizing antibody. We found that the antidepressant‐like effects of *R*‐ketamine were not detected in mice with BDNF knockdown in the LC^TH^‐dLS pathway and with anti‐BDNF neutralizing antibody infusion into the dLS (Figure S23A–H, Supporting Information). These results indicate that BDNF in the LC is required for the sustained antidepressant‐like effects of *S*‐ketamine and *R*‐ketamine.

Fluoxetine is a potent psychotropic drug that acts as a selective serotonin reuptake inhibitor (SSRI) by blocking the plasma membrane serotonin transporter (SERT).^[^ ^46^ ^]^ We also tested whether these mice responded to fluoxetine (Figure 7E,F). We found that chronic fluoxetine treatment reversed depression‐like behavior after CSDS, but the knockout of BDNF in the LC^TH^‐dLS circuit did not block the antidepressant effect of fluoxetine (Figure 7G,H). These data suggest that while BDNF expression is associated with the treatment efficacy of both ketamine and fluoxetine, the underlying mechanism of action of fluoxetine is different from that of ketamine.

## 3 Discussion

In this study, we found that the repeated activation of LC^TH^ neurons exerted significant antidepressant‐like effects. Furthermore, chemogenetic and optogenetic manipulation of the LC^TH^‐dLS^SST^ circuit bidirectionally regulated depression‐like behaviors. Mechanistically, this regulation occurs by promoting BDNF signaling from the LC to the dLS. It is worth noting that BDNF in this circuit is essential for both *S*‐ketamine and *R*‐ketamine to exert their antidepressant‐like effects. Taken together, these findings add to the growing evidence for the involvement of the LC^TH^‐dLS circuit in the pathophysiology of depression.

LC neurons have long been considered key mediators of stress responses.^[^ ^47^ ^]^ In this study, to cover more facets, we combined immunohistochemistry, anterograde and retrograde viral tracing methods, immediate early gene expression, calcium recording, and behavioral tests to identify the dLS is a key downstream node in processing stress from the LC. Since mPFC and CeA are important regions associated with depression, we also conducted experiments to investigate the relevance of LC^TH^‐mPFC and LC^TH^‐CeA to depression‐like behaviors. In Figure 3, we observed that the calcium signals were barely detectable in the LC^TH^‐mPFC and LC^TH^‐CeA circuits in the SIT. Immunohistochemistry showed minimal overlap between the three LC populations, suggesting that they arose from distinct LC subpopulations. Moreover, functional behavioral tests among the identified downstream brain regions of the LC further confirmed that LC inputs to the dLS, rather than the mPFC or CeA, played a particularly significant role in depression (Figure S10, Supporting Information). Furthermore, studies have reported that LC to the mPFC or CeA is more relevant to anxiety‐like behaviors and behavioral flexibility.^[^ ^48^, ^49^ ^]^ However, whether and how LC^TH^‐ mPFC and LC^TH^‐ CeA circuits are related to anxiety‐like behaviors needs to be further investigated.

In this study, we employed RNA‐sequence analysis, pharmacological profiling, and gain‐ or loss‐of‐function genetic approaches to demonstrate that BDNF, rather than NE, was crucial for the LC^TH^‐dLS circuit to induce antidepressant‐like effects. Previous studies have reported that NE enhances excitatory synaptic transmission to LS^SST^ neurons, but does not affect stress‐related behaviors.^[^ ^50^ ^]^ This was consistent with our finding that NE was not required for the LC^TH^‐dLS circuit to alleviate social avoidance following social trauma. We acknowledge that BDNF has been extensively studied in depression, yet there is no consensus in the literature.^[^ ^40^, ^51^, ^52^, ^53^ ^]^ These inconsistent results may be a consequence of BDNF being modulated in different brain regions, time windows, and stress types. For example, the knockdown of BDNF in the dorsolateral PFC (dlPFC) is sufficient to induce MDD‐ and stress‐related phenotypes.^[^ ^53^ ^]^ While knockdown of BDNF in a brainstem nucleus such as the dorsal raphe nucleus (DRN) resulted in no impairment in depression‐like behaviors as assessed in the FST and SPT.^[^ ^54^, ^55^, ^56^ ^]^ In this study, we demonstrated that optogenetic activation of LC^TH^ neurons induced a marked increase in BDNF protein levels in the dLS and K252a or BDNF‐nAb disrupted the antidepressant‐like effects produced by the activation of the circuit. It was consistent with a previous study that reported BDNF release within the mPFC is necessary for the antidepressant actions of ketamine.^[^ ^57^ ^]^ Moreover, in this study, supplementation of BDNF in the dLS or overexpression of AAV‐BDNF in the LC could significantly alleviate the depression‐like behaviors caused by CSDS. However, there are some limitations in our study. For example, in Figure 5E–H, when the LC is activated, excitatory neurotransmitters like glutamate are also released from the LC. We cannot rule out the possibility that glutamate within this circuit also plays an antidepressant role. In addition, in Figure 6, the supplementation of BDNF in the dLS or the overexpression of AAV‐BDNF in the LC occurred only at the local nuclei level, rather than at the circuit level. Future studies will be important in elucidating the role of glutamate within the circuit and in investigating the effects of BDNF overexpression on depression‐like behavior at the circuit level.

It is well known that BDNF binds to its high‐affinity TrkB receptor to activate distinct signaling pathways. TrkB activation leads to the phosphorylation of phospholipase C‐γ (PLC‐γ), which induces the generation of inositol trisphosphate (IP3) and the subsequent release of Ca^2+^ from intracellular stores to promote synaptic plasticity.^[^ ^40^, ^58^, ^59^ ^]^ However, in our study inhibiting the TrkB receptor in the dLS alone was not sufficient to induce depression‐like behaviors. It was consistent with previous studies that reported TrkB dominant‐negative genotypes, which show reduced TrkB activation in the brain, could not induce depressive phenotypes.^[^ ^54^ ^]^ We speculate that depression‐like behaviors may not be triggered by deficits in TrkB signaling alone but, rather, may require impairments in multiple pathways. Alternatively, inhibition of TrkB receptors could be compensated by the upregulation of other receptors. Perhaps one factor could increase vulnerability but is insufficient alone to produce depression‐like behaviors. Moreover, *BDNF^flox/+^* mice with BDNF knockout in LC^TH^ neurons and then infusion of BDNF protein in the dLS exhibited a significant reduction—but did not completely prevent—the response to stress (Figure 6J–P), further implying that other factors contribute to social behaviors induced by chronic stress.

Ketamine, an antidepressant that can induce a response within hours, is thought to inhibit NMDA receptors involved in spontaneous transmission.^[^ ^32^, ^60^, ^61^ ^]^ In this study, we found that BDNF in the LC^TH^‐dLS circuit is necessary for the sustained antidepressant‐like effects of *S*‐ketamine and *R*‐ketamine. However, chemogenetic or optogenetic stimulation of LC^TH^‐dLS circuit took as long as 10 days to become effective, which seems contradictory. We hypothesized that *S*‐ketamine and *R*‐ketamine may rapidly increase the levels of BDNF in the LC. While chemogenetic or optogenetic manipulations may produce a slow induction of BDNF release, and then require more time to alter the structure and function of neural networks, as well as the expression of relevant proteins. This process may necessitate a certain amount of time to initiate and sustain these changes. Thus, it is not contradictory that *S*‐ketamine and *R*‐ketamine produce rapid and sustained responses after a single injection while chemogenetic or optogenetic manipulations require 10 days of stimulation. Moreover, in Figure 7E–H, we have demonstrated that BDNF‐TrkB signaling in the LC^TH^‐dLS circuit is not essential for the antidepressant‐like effects of fluoxetine. A recent paper suggests that fluoxetine and ketamine acts by directly binding to TrKB receptors.^[^ ^33^ ^]^ Antidepressant binding stabilizes TrkB in synaptic membranes and promotes BDNF‐mediated TrkB signaling.^[^ ^33^ ^]^ However, fluoxetine binds to serotonin transporter (5HTT) with a much higher affinity than to TrkB, while ketamine has a high affinity for TrkB receptors.^[^ ^33^, ^62^ ^]^ Therefore, we speculate that after inhibiting the BDNF‐TrkB signaling in the LC^TH^‐dLS circuit, fluoxetine may still exert antidepressant‐like effects through the 5HTT signaling pathway. Furthermore, it is proposed that ketamine inhibits NMDA receptors on inhibitory neurons, resulting in raised levels of extracellular glutamate and activation of AMPA receptors.^[^ ^40^, ^63^, ^64^, ^65^ ^]^ This in turn prompts the release of BDNF to exert the antidepressant response.^[^ ^65^ ^]^ In our study, the activation of SST neurons induced by BDNF‐TrkB signaling in the dLS leads to antidepressant‐like effects, which contradicts the hypothesis suggesting an initial inhibition of SST neurons and disinhibition of glutamatergic cells by ketamine. Ketamine is a known non‐competitive NMDA receptor antagonist that blocks the ion permeation pore within these receptors.^[^ ^66^ ^]^ However, ketamine cannot enter the channel pore and block NMDA receptors if they are closed.^[^ ^67^ ^]^ Our study revealed a significant decrease in the activity of SST neurons in the dLS during depression‐like states. This suggests that the channel pores of NMDARs in SST neurons may predominantly be closed, preventing ketamine from entering and blocking them. Moreover, meta‐analyses have shown that the effect sizes of ketamine are larger than those of other NMDAR antagonists,^[^ ^68^, ^69^ ^]^ indicating other mechanisms also contribute to the robust antidepressant effects of ketamine. Importantly, a recent study has demonstrated that antidepressants such as ketamine directly bind to TrkB receptors with high affinity.^[^ ^33^ ^]^ Ketamine stabilizes a configuration of TrkB dimers that promote the binding of BDNF, thereby allosterically enhancing the effects of BDNF on TrkB.^[^ ^33^ ^]^ This potentially results in the activation of SST neurons. The lateral habenula (LHb) is recognized as a significant downstream target of the dLS, and aberrant overactivity within LHb plays a crucial role in depression.^[^ ^70^ ^]^ Inhibiting the activity of LHb neurons can alleviate depression‐like behaviors. We hypothesize that ketamine may directly bind to TrkB, promoting the binding of BDNF onto TrkB, and subsequently activating the dLS^SST^ neurons. As a result, SST neurons transmit inhibitory synaptic information to the LHb nucleus, promoting inhibitory effects on LHb neurons and ultimately ameliorating depressive‐like behaviors. However, these are all our hypotheses, lacking experimental evidence and it is crucial to investigate this issue in future work.

Taken together, our findings demonstrate the critical role of BDNF signaling in the LC^TH^‐dLS^SST^ circuit in the pathogenesis of depression. Moreover, our study provides specific molecular, cellular, and behavioral evidence for the function of the LC^TH^‐dLS^SST^ pathway, which may shed light on the pathophysiology of depression.

## 4 Experimental Section

### Animals

All animal care procedures were performed in accordance with the National and International Guidelines of the Animal Resource Center of Nanjing Medical University (approval number I ACUC‐22050569). Mice, housed in a 12 h light‐dark cycle, were given standard food and water ad libitum. Adult C57BL/6J mice (male and female, 8 weeks old) and CD1 mice (male, 30–35 weeks old) were purchased from the Animal Resource Center of Nanjing Medical University. *BDNF^flox/+^* mice (strain no. T013188) were purchased from Gem Pharmatech (Nanjing, China). *BDNF^flox/+^* mice were crossed with *TH‐Cre* mice to generate *TH‐Cre;BDNF^flox/+^* mice (*BDNF‐cKO*). To produce *TH‐Cre;Ai14* mice, *TH‐Cre* mice were crossed with *B6.Cg‐Gt(ROSA)26Sor^tm14(CAGtdTomato) Hze^/J (Ai14)* mice (stock no. 007914; Jackson Laboratory).

### Virus Vectors and Drugs

The viruses: AAV2/9‐EF1α‐DIO‐Gcamp6s, AAV2/9‐EF1α‐DIO‐EGFP, AAV2/9‐DIO‐hM3Dq‐mCherry, AAV2/9‐DIO‐hM4Di‐mCherry, AAV2/9‐hSyn‐DO‐hM3Dq‐EGFP, AAV2/9‐DIO‐ChR2‐mCherry, AAV_retro_‐EF1α‐DIO‐flp, AAV2/9‐hSyn‐fDIO‐hM3Dq‐mCherry, AAV2/9‐hSyn‐fDIO‐hM4Di‐mCherry, AAV_retro_‐TH‐Cre, AAV_retro_‐EF1α‐DIO‐EGFP, AAV_retro_‐EF1α‐DIO‐mCherry, AAV2/9‐EF1α‐DIO‐RVG, AAV2/9‐EF1α‐DIO‐TVA‐EGFP, RV‐ENVA‐ΔG‐DsRed, AAV2/9‐TH‐Cre‐EGFP, AAV2/9‐TH‐EGFP, AAV‐hSyn‐NE2m were purchased from BrainVTA (Wuhan, China). For infection of PV and SST neurons, AAV‐PV‐Cre virus (5.27 × 10^12^ vector genomes mL^−1^) and AAV‐SST‐Cre virus (5.02 × 10^12^ vector genomes mL^−1^) were purchased from Obio Technology (Shanghai, China). The retrograde tracer Cholera Toxin Subunit B (CTB‐488) was purchased from Brain Case (Wuhan, China). Viral titers ranged from 2 to 8 × 10^12^ vector genomes mL^−1^. Viruses were subdivided into aliquots and stored at −80 °C until used.

For BDNF overexpression, the coding regions of BDNF were amplified by PCR and then cloned into pAAV‐EF1α‐DIO‐EGFP‐3FLAG. The procedures were commercially developed by Obio Technology (Shanghai, China). The titers of the virus were: pAAV2/9‐EF1α‐DIO‐EGFP‐BDNF‐3FLAG (AAV‐BDNF), 1.15 × 10^13^ vector genomes mL^−1^, or its control pAAV2/9‐EF1α‐DIO‐EGFP‐3FLAG (AAV‐Con), 1.35 × 10^13^ vector genomes mL^−1^. The samples were diluted to 5 × 10^12^ with PBS prior to the experiments.

For BDNF knockdown, a short hairpin RNA (shRNA) targeting BDNF pAAV2/9‐CMV‐DIO‐EGFP‐miR30‐WPRE was bought from Obio Technology (Shanghai, China). The titers of the virus were: pAAV2/9‐CMV‐DIO‐EGFP‐miR30‐shRNA (BDNF)‐WPRE (BDNF‐shRNA), 1.02 × 10^13^ vector genomes mL^−1^; pAAV2/9‐CMV‐DIO‐EGFP‐miR30‐shRNA (NC)‐WPRE (Con‐shRNA), 1.32 × 10^13^ vector genomes mL^−1^. The sequences of con‐shRNA and BDNF‐shRNA were 5’‐CCTAAGGTTAAGTCGCCCTCG‐3’ and 5′‐GGTGATGCTCAGCAGTCAAGT‐3′, respectively.

Drugs were prepared: CNO (1 mg kg^−1^, i.p., diluted in 0.9% saline; 3 mM, 1 μL per site, CAS NO.: C0832; Sigma‐Aldrich), K252a (1 μM, 1 μL per site, CAS NO.:99533‐80‐9, Sigma‐Aldrich), propranolol (5 mM, 1 μL per site, CAS NO.:318‐98‐9, Sigma‐Aldrich), phentolamine (10 mM, 1 μL per site, CAS NO.: 73‐05‐2, Sigma‐Aldrich), Recombinant Human BDNF Protein (200 ng in 0.9% saline for local injection, 1 μL per site, CAS NO.:248‐BDB; R&D Systems), anti‐BDNF neutralizing antibody (0.2 µg in 0.9% saline for local injection, 0.2 μL per site, EMD Millipore), *S*‐ketamine (10 mg kg^−1^, i.p., diluted in 0.9% saline, CAS NO.:1430202‐70‐2, Sigma‐Aldrich), *R*‐ketamine (10 mg kg^−1^, i.p., diluted in 0.9% saline, CAS NO.: 1430202‐69‐9, Sigma‐Aldrich), and fluoxetine (20 mg kg^−1^ i.p., diluted in 0.9% saline, CAS NO.:56296‐78‐7, Sigma‐Aldrich). Drug concentrations for intraperitoneal and intracranial injections were as previously described.^[^ ^11^, ^23^, ^30^, ^60^, ^71^, ^72^, ^73^, ^74^ ^]^

### Stereotaxic Injection

As frequently performed,^[^ ^11^ ^]^ mice were deeply anesthetized using sodium pentobarbital (50 mg kg^−1^, i.p. injection) and fixed to a stereotaxic apparatus (RWD Life Science) with a heating pad to maintain body temperature during surgery. After the dura was exposed, a pulled glass micropipette coupled with a syringe pump (RWD Life Science) was used for virus infusion into the brain; the microinjectors were held in place for 10 min after injection and then slowly withdrawn. After suturing, the mice were placed in a warm environment and allowed to recover for 3 days with care. The infusion rate for the virus and CTB was 30 nL min^−1^, with total volumes of 100–300 nL per side.

For the experiment tracing output areas of LC^TH^ neurons, *TH‐Cre* mice were unilaterally injected with 150 nL of AAV2/9‐EF1α‐DIO‐ChR2‐EGFP into the LC regions (AP: ‐5.3 mm; ML: +0.8 mm; DV: ‐4.0 mm; relative to bregma). To characterize neuronal populations of LC based on downstream projection targets, 150 nL AAV_retro_‐EF1α‐DIO‐EGFP or AAV_retro_‐EF1α‐DIO‐mCherry was unilaterally injected into the mPFC (AP: +1.5 mm; ML: +0.35 mm; DV: ‐2.5 mm; relative to bregma), dLS (AP: +0.35 mm; ML: +0.35 mm; DV: ‐2.6 mm; relative to bregma), or CeA (AP: ‐1.22 mm; ML: +2.6 mm; DV: ‐4.5 mm; relative to bregma) of *TH‐Cre* mice. For monosynaptic retrograde tracing, helper viruses (AAV2/9‐DIO‐RVG and AAV2/9‐DIO‐TVA‐EGFP) were mixed with AAV2/9‐SST‐Cre or AAV2/9‐PV‐Cre and then unilaterally co‐injected into the dLS of mice a total volume of 300 nL. Three weeks later, the rabies virus RV‐ENVA‐ΔG‐DsRed was injected into the same site in dLS. To delete BDNF in the LC^TH^‐dLS, AAV‐DIO‐BDNF‐shRNA‐EGFP was bilaterally injected into the LC, and AAV_retro‐_TH‐Cre was injected into the dLS. To delete BDNF in the LC, AAV‐DIO‐TH‐Cre‐EGFP was bilaterally microinfused into the LC of *BDNF^flox/+^* mice. For overexpression of the BDNF, the pAAV2/9‐EF1α‐DIO‐EGFP‐BDNF‐3FLAG was bilaterally microinfused into the LC of *TH‐Cre; BDNF^flox/+^* mice. Data from mice with incorrect injection sites or plantation locations were excluded.

### In Vivo Fiber Photometry

Ca^2+^ signals were recorded using a fiber photometry system (Thinker Tech Co., Nanjing, China). To capture fluorescence signals from GCaMP6s, a 470 nm LED light was filtered with a bandpass filter, collimated, reflected by dichroic mirrors, and focused using a 20× objective. The laser power at the tip of the optical fiber was adjusted to 40–60 µW to minimize bleaching of the GCaMP6s probes. To serve as an isosbestic control channel, a 405 nm LED light was delivered alternately with a 470 nm LED light. The Thinker Tech software was utilized to calculate the mean value for each region of interest (ROI) on the fiber's end‐face. To record the Ca^2+^ signals of LC^TH^ neurons, AAV2/9‐EF1α‐DIO‐Gcamp6s or AAV2/9‐EF1α‐DIO‐EGFP was injected into LC of *TH‐Cre* mice. To record the Ca^2+^ signals of LC^TH^ neurons projecting to mPFC, dLS, or CeA, AAV2/9‐EF1α‐DIO‐Gcamp6s was injected in the LC along with AAV_retro_‐TH‐Cre injected to the downstream regions. One week after injection, the optical fiber (diameter, 200 µm; fiber length, 5.0 mm; numerical aperture (NA, 0.37) housed in a ceramic ferrule was implanted 0.2 mm above LC. Fiber recordings were performed in behaving mice during the SIT. Neural activity was captured using a photometry recording system when a novel CD1 mouse was placed in an empty cage. In Figures 1E and 3B,D,F, the “Approach” refers to the mice entering the social interaction zone. To record the Ca^2+^ signals of the FST, we monitor the entire swimming process. The mobility time start was defined as at least 2 s of mobility time followed by at least 2 s of complete immobility. Moreover, the bout of licks in the study was defined as one or more licks separated by a minimum interval of 10 s based on a previous study.^[^ ^75^ ^]^ Calcium data and behavioral videos were aligned offline using event markers. The data were processed using the MATLAB program developed by Thinkertech. The values of fluorescence change were derived by calculating *F*/*F*_0_, where *F* is the variation of fluorescence between each sampling (sampling frequency 100Hz), and *F*_0_ was the averaged fluorescence baseline in the whole duration of the entire test (2 min Fluorescence change (Δ*F/F*) value was defined as *(F‐F_0_)/F_0_* and presented with heatmaps and average plots.

### In Vivo Optogenetic and Chemogenetic Manipulation

For optogenetic activation of the LC^TH^‐dLS circuit experiments, mice were exposed to 10 days of CSDS and then received blue light with the same pattern (473 nm, 10 Hz, 5 pulses/20 s) 20 min per day for 10 days on the dLS. For chemogenetic manipulations, two or three weeks after the injection of AAVs expressing hM4Di‐mCherry or hM3Dq‐mCherry, CNO was administrated intraperitoneally (1 mg kg^−1^) or locally (3 μM).

### Cannula Implantation

Cannula implantation was performed as described previously. The surgical procedure was the same as that described for the virus injection. A bilateral cannula (RWD Life Science) was implanted in the region of LC (AP: ‐5.3 mm; ML: ±0.8 mm; DV: ‐3.8 mm; relative to bregma) or dLS (AP: +0.35 mm; ML: ±0.35 mm; DV: ‐2.4 mm; relative to bregma) and was fixed to the skull with dental cement. After the surgery, a dummy cannula was inserted into the guide cannula to seal the opening. The mice were allowed to rest for one week, during which they were handled gently and gradually habituated to the daily infusion procedure. In order to prevent damage to the dLS, the injection cannulae were designed to protrude 0.20 mm from the tip of the guide cannulae and thus penetrate into the LC or dLS. Therefore, it would not damage the brain regions after 10 days of chronic infusion. Following infusion, the injection cannula was left in the dLS for an additional 5–7 min to allow the drug solution to diffuse from the cannula tips. To clearly check the drug infusion sites, mice were injected with 150–250 nL of CTB‐488 on each side of the dLS after all behavioral tests. Only the correct cannula locations were used for further analyses.

For pharmacological experiments, a bilateral cannula (RWD Life Science) was implanted above the dLS for infusion of ß‐adrenergic receptor antagonist propranolol (5 mM) or BDNF receptor TrkB receptor antagonist K252a (1 μM) 30 min before CNO. Regarding overexpression of BDNF in the LC^TH^‐dLS circuit, recombinant human BDNF protein (200 ng, 1μL per side) was infused into LC or dLS.

### Fluorescence‐Activated Cell Sorting of LC Neurons

As previously described,^[^ ^76^ ^]^ mice were deeply anesthetized and perfused with cold sterile PBS to remove blood cells. The LC regions were microdissected on ice from sections (1000 µm thick) sliced using rodent brain matrices. Tissue pieces were coarsely chopped and enzymatically incubated in 1 mL HBSS (CAS NO.:14175103; Gibco) digestion solution containing 2 mg mL^−1^ papain (CAS NO.: p4762; Sigma‐Aldrich), 100 µg mL^−1^ DNase I (CAS NO.:10104159001; ROCHE), and 0.5 mg mL^−1^ EDTA for 30 min at 37 °C. The cells were dissociated with a P1000 wide‐bore pipette tip four times every 10 min. After digestion, cell suspensions were filtered by 70‐µm filters and centrifuged at 500 × g for 5 min with supernatants discarded. To remove myelin debris, cell pellets were resuspended in 15% Percoll solution and centrifuged at 1000 × g for 10 min. Supernatants were removed, and samples were resuspended in 0.3 mL of PBS with 5% FBS and kept on ice.

Cells were sorted utilizing a fluorescent‐activated cell sorting, the Aria Fusion System (BD Biosciences) with a nozzle diameter of 100 mm directly into 500 μL TRIzol (CAS NO.: YFXM0011; YIFEIXUE BIO TECHNOLOGY). TH neurons were labeled with EGFP. DAPI was added 5 min prior to sorting. DAPI^−^ and EGFP^+^ cells were identified as positive targets for the isolation.

### In Vivo Electrophysiology Recordings

Adult wild‐type mice were injected 100 nL with recombinant AAV vectors (AAV_2/9_‐DIO‐ChR2‐mCherry in LC, AAV_retro‐_TH‐Cre in dLS) unilateral. A week later, an 8‐channel electrode containing a cannula is implanted into the dLS, Electrodes consisted of eight individually insulated nichrome wires (35‐µm inner diameter, impedance 300–900 Kohm; Stablohm 675, California Fine Wire, U.S.A.) and one cannula (RWD Life Technology Co. Ltd., China) in center. Three weeks after virus injection, neural signals were simultaneously recorded using a multichannel data‐acquisition system (Zeus, Bio‐Signal Technologies: McKinney, TX, U.S.A). Offline Sorter (Plexon: Dallas, TX, U.S.A.) was used for spike‐sorting, and analyzing data in NeuroExplorer 5 (Nex Technologies: Boston, MA, U.S.A.). Neurons were selected that responded to the 10 Hz stimulation during the “light on” epoch for further study. After infusing either saline or K252a through a cannula in the dLS, the spike frequency was compared in 90 s windows: 30 s before light onset, 30 s during light on, and 30 s after light off.

### RNA Sequencing (RNA‐seq)

A library was constructed and sequenced using Capital Bio‐Technology (Beijing, China). Total messenger RNA (mRNA) was extracted from the LC tissues of susceptible or control mice using TRIzol (Tiangen, Beijing). Qualified samples with standard RNA integrity numbers (RIN) > 7.0 and 28S:18S ratio > 1.8 were used for library construction. Briefly, mRNA was purified from 1µg total RNA and fragmented into pieces (≈200 base pairs) using the NEB Next Ultra RNA Library Prep Kit for Illumina (New England Biolabs, Beijing, China). First‐strand and cDNA synthesis with some modifications (a single “A” base connected to the adapters) were performed and then amplified by polymerase chain reaction, finally quantified by an Agilent 2100 Bioanalyzer. The libraries were sequenced after cluster generation on a HiSeq sequencer (Illumina) using paired‐end chemistry (PE150).

Fast QC (version 0.11.5) was used to assess sequence read quality, and NGSQC (v0.4) was used to filter low‐quality data. HISAT2 (Johns Hopkins University, USA) was used to align the clean reads with the reference genome. Gene expression analyses were performed using StringTie and DEGs between samples were analyzed using DESeq2. DEGs with significance are classified using |log_2_FC| ≥ 1 (FC: fold change in expression) and *q* < 0.05.

### Chronic Social Defeat Stress (CSDS)

The CSDS paradigm was conducted as described by Golden et al.^[^ ^37^ ^]^ Briefly, male CD1 aggressors were screened for their ability to defeat intruders. Over a period of 10 days, each mouse was forced to become an intruder and placed into the home cage of a novel male CD1 aggressor to experience physical defeat for 5 min per day (referred to as the “physical contact period”). After the physical interaction, the defeated mouse experiences sustained psychological stress from sensory interaction with the aggressor for the duration of the experiment through a clear perforated translucent Plexiglas barrier in a shared home cage (referred to as the “sensory interaction”). After 10 days of CSDS, aggressors were removed, and the mice were singly housed for the next stage of the experiment. Undefeated control mice were housed in pairs with a barrier separating them from each other and were handled daily for 10 days. For defeating female mice, a previously described paradigm was used.^[^ ^36^ ^]^ The urine was exploited by females to enhance aggressive behavior in male mice. The method of urine collection involved placing CD1 mice in plastic containers with raised wire caging on the bottom for one hour. Urine excreted throughout this duration was gathered from the container's base and stored at a temperature of 4 °C. During the social defeat period, female C57BL/6 mice were exposed daily (5 min per day) to episodes of social defeat by a larger, physically aggressive CD‐1 male mouse. This exposure occurs after applying specific male CD1 mouse urine to the vagina, tail base, and upper back of the female. Each female mouse was paired with the urine of a particular CD1 mouse that was unknown to the resident CD1 aggressors throughout the entire course of social defeat. Male urine was essential to initiate aggression toward females and parallels the classic male‐to‐male 10‐day CSDS protocol. In order to avoid gender differences during social interaction between male CD1 mice and female model mice, the same method was used as modeling to smear urine from male CD1 mice on each tested female mouse to reduce sex differences during SIT. An SIT was conducted 24 h after the last exposure to social stress. Defeated mice that display social avoidance were considered susceptible (Sus).

### Three‐Day Subthreshold Social Defeat Stress (SSDS)

This paradigm ^[^ ^77^ ^]^ was in accordance with the normal CSDS procedure, except that the procedure time was reduced to 3 consecutive days.

### Social Interaction Test (SIT)

This test was performed a day after the last defeat to determine social avoidance behavior and comprised two phases. In the first step, mice were placed in an open arena (50 cm × 50 cm× 50 cm) with an empty wire mesh enclosure (9.5 cm wide × 6.5 cm deep × 50 cm high) placed along the center of one side. The movements of the animals in the arena were monitored for 2.5 min in the first phase using a TopScan RealTime Option tracking system (Version 2.00, Clever Sys Inc., USA). The animals were then removed from the arena and allowed to rest for a short period. In the second phase, a new aggressor male CD1 mouse was placed in the enclosure, and the mice were reintroduced to the recording arena for another 2.5 min. Before each trial, 30% ethanol was sprayed onto the apparatus to remove olfactory cues from the experimental mice. The Social interaction ratio (SIR) was calculated by dividing the time spent in the interaction zone when the target was present by the time spent there when the target was absent. Animals were defined as susceptible (Sus) if SIR < 1 (social avoidance), and resilient (Res) if SIR ≥ 1 (social preference).

### Open Field Test (OFT)

An open‐field area (50 × 50 × 50 cm) adjusted for illumination was used to assess locomotor activity. Mice were introduced into the center zone of the field and allowed to freely explore the arena for 10 min. A TopScan video‐tracking system (version 2.00, Clever Sys Inc., USA) was used to record and measure the animal locomotor activity. The total distance traveled in the entire open‐field arena over 10 min was analyzed.

### Forced Swim Test (FST)

Mice were forced to swim in a cylinder (20 cm in diameter, 28 cm in height) of water at a temperature of 22 ± 1 °C. Immobility was defined as floating without any movement or with only the necessary movements to maintain balance in the water. The total swimming lasted for 6 min and was video‐tracked by ForcedSwimScan (Clever Sys Inc., USA). The duration of immobile gestures during the last 4 min was analyzed.

### Tail Suspension Test (TST)

The mice were suspended upside down 25 cm above the floor using an adhesive tape attached to the tip of the tail. Plastic pipes were placed over the tail to prevent the mouse from climbing up the tail. Mice that hung passively or were completely motionless were considered immobile. The total suspension lasted for 6 min and was video‐tracked by ForcedSwimScan (Clever Sys Inc., USA). The time spent immobile during the last 4 min was analyzed.

### Sucrose Preference Test (SPT)

The preference for 1% sucrose solution over plain water was tested using a two‐bottle choice procedure. During the first 2 days, the mice were habituated to two bottles containing 1% sucrose and pure water. The bottle locations were switched every 12 h to reduce the potential influence of side preference. After habituation, mice were deprived of water and food for 12 h. The mice were then introduced to two pre‐weighed bottles for the next two days with their locations interchanging after each weight measurement (every 8 h). Based on the percentage of sucrose solution to the total liquid consumed, the sucrose preference index was calculated to describe the preference for sucrose solutions.

### Western Blot (WB)

LC or dLS tissues (≈0.5 mg) from susceptible or control mice were lysed in RIPA buffer containing a protease inhibitor cocktail (CAS No. A32955, Invitrogen). The lysates were centrifuged at 12 000 × g for 15 min at 4 °C after lysis, and the supernatants were used for immunoblotting on 15% gels and then transferred to polyvinylidene fluoride (PVDF) membrane. After that, the blots were blocked with 5% nonfat dry milk in TBST for 3 h at room temperature and then incubated with primary antibodies overnight at 4 °C. The next day, the membranes were washed with TBST and incubated with horseradish peroxidase‐conjugated anti‐rabbit antibody (1:3000) or anti‐mouse antibody (1:3000) for 1 h at room temperature. After three washes with TBST, the antibody‐reactive bands were visualized using enhanced chemiluminescence (ECL) detection reagents (1:1, GE Healthcare) and a gel imaging system (Tanon, Shanghai, China). The primary antibodies used in assays were: BDNF (1:500, CAS NO.: ab108319, Abcam) and β‐actin (1:3000, CAS NO.: A5441, Sigma‐Aldrich). Quantification was performed using the ImageJ software.

### Quantitative Real‐Time Reverse Transcription PCR (qRT‐PCR)

LC tissue samples and LC^TH^‐dLS circuit cells were collected as described above. Total RNA was extracted using a TRIzol reagent. Reverse transcription was performed using a HiScript III 1st Strand cDNA Synthesis Kit (CAS NO.: R312‐01, Vazyme, China). All real‐time PCR reactions were performed by using the stepOnePlus instrument (Applied Biosystems, USA) and ChamQTM SYBR qPCR Master Mix Kit (CAS NO.: Q311‐02, Vazyme). The target gene expression was calculated as 2^−ΔΔCt^ method. The primers used were: *BDNF* mRNA (forward:5’‐TCATACTTCGGTTGCATGAAGG‐3’; reverse:5’‐ACACCTGGGTAGGCCAAGTT‐3’), *GAPDH* mRNA (forward:5’‐AGGTCGGTGTGAACGGATTTG‐3’; reverse:5’‐TGTAGACCATGTAGTTGAGGTCA‐3’), *Shank1* mRNA (forward:5’‐TGCATCAGACGAAATGCCTAC‐3’; reverse:5’‐AACAGTCCATAGTTCAGCACG‐3’), *Shank3* mRNA (forward:5’‐GCCATTTCAACAGAAGCCCC‐3’; reverse:5’‐CGCCTTCGATCTCATGGTCC‐3’), and *Pllp* mRNA (forward:5’‐GCCTATGGCTGGGTCATGTT‐3’; reverse:5’‐ATGTAGAGAACCGTGGCAGC‐3’) based on previously published results. *GAPDH* served as an internal control.

### Immunofluorescence (IF)

The mice were deeply anesthetized and intracardially perfused with PBS, followed by 4% PFA. The brains were harvested, post‐fixed in 4% PFA overnight, dehydrated in 20% sucrose in PBS, and then transferred to 30% sucrose‐PBS until isotonic. Coronal sections (30 µm) were sliced using a freezing microtome (Leica CM1950) and mounted on slides with PBS. For immunofluorescence staining, slices were permeabilized and blocked by 3% bovine serum albumin (BSA) and 5% goat serum in 0.25% Tween‐20/PBS (PBST) at 37 °C for 1 h. The sections were then incubated with primary antibodies in a blocking buffer at 4 °C for 16 h. The primary antibodies used were as follows: rabbit anti‐TH (1:600, CAS No.:25859‐1‐AP, Proteintech), rabbit anti‐c‐Fos (1:800, CAS No.:2250S, CST), rabbit anti‐BDNF (1:400, CAS No.: ab108319, Abcam), mouse anti‐GFAP (1:400, CAS No.: MAB360, Millipore), rabbit anti‐Iba‐1 (1:400, CAS No.: PA5‐27436, Invitrogen), mouse anti‐SST(1:100, CAS No.:sc‐74556, Santa Cruz), rabbit anti‐PV (1:100, CAS No.:ab‐181086, Abcam) and mouse anti‐TrkB(1:100, CAS No.:sc‐377218, Santa Cruz). After washing with PBST, sections were incubated with secondary antibodies conjugated with either Alexa Fluor 488 or Alexa Fluor 594 at 37 °C for 1.5 h. After washing, DAPI (1:1000, CAS No.: C0065; Solarbio) was used to visualize the nuclei. The sections were coverslipped with an anti‐fluorescence quencher and mounted on the cover glass.

Multiple TSA staining was performed when the primary antibodies were raised in the same host species. For each antibody, the brain slices were permeabilized and blocked at 37 °C for 1 h in blocking buffer and then incubated with the primary antibody at 4 °C overnight. Washed with PBST, sections were incubated with secondary antibody conjugated with HRP at 37 °C for 1.5 h followed by TSA‐dendron‐fluorophores (1:150, NEON 4‐color IHC Kit for cryosection, Histova) for 1 min. After the chromogenic reaction, the sections were washed in stripping solution (Histova, Abcracker) at 37 °C until all combinations were eluted. The previous steps were repeated, and each antigen was labeled with different fluorophores. Images were acquired using a THUNDER tissue imager (Leica Thunder). Cells were manually by a blinded experimenter and counted using ImageJ. Based on previous study,^[^ ^78^ ^]^ for the counting of mCherry‐expressing or EGFP‐expressing LC^TH^ neurons, three sections from each animal were selected (From Bregma ‐5.34 mm, ‐5.40 mm, ‐5.52 mm). Next, we manually counted the EGFP^+^+mCherry^+^ double‐positive cells and divided them by the total number of mCherry^+^ or EGFP^+^ neurons to represent the co‐localization percentage (%). For the counting of c‐Fos positive neurons in dLS or other regions, three slices from each brain region of 3–5 animals were selected. Next, the appropriate areas were selected, and then manually counted the number of the c‐Fos positive neurons within the area, was averaged in all three slices, treated as a value of a subject, and then converted to mm^2^ unit.

### Statistical Analysis

For two normally distributed groups, the differences were calculated using an unpaired two‐tailed Student's *t‐*test. Single‐factor experiments with >2 groups were analyzed using one‐way ANOVA with post hoc Tukey's multiple comparison. Two‐way ANOVA followed by Bonferroni's test was used for double‐factor experiments. All statistical analyses were performed in a blinded manner using GraphPad Prism 8 software. All results were represented as mean ± S.E.M. ^*^ *p <* 0.05, ^**^ *p <* 0.01, and ^***^ *p <* 0.001 was considered to be statistically significant. The statistical details can be found in Table S1, Supporting Information.

## Conflict of Interest

The authors declare no conflict of interest.

## Author Contributions

Q.Z., Y.X., and K.W. contributed equally to this work. G.H., and Q.Z., designed the experiments. Q.Z. and Y.X. performed immunostaining and behavior tests. Q.Z. and K.W. performed stereotactic injection, cannula implantation, western blot, and q‐PCR. Y.X., Y.F., and Y.M. performed the in vivo activity recordings. H.W., Y.W., Y.M, L.G., H.Y. assisted with behavioral experiments, histology, and microscopy. H.W., Y.W., F.F.W., X.D, Q.Y.Z., J.H.D., Y.F., and M.L. provide experimental guide. Q.Z., Y.X., and K.W. analyzed the data and wrote the manuscript. G.H. supervised this research and edited the paper.

## Supporting information

Supporting Information

## Data Availability

The data that support the findings of this study are available on request from the corresponding author. The data are not publicly available due to privacy or ethical restrictions.
