# Supplementary material for: Locus Coeruleus‐Dorsolateral Septum Projections Modulate Depression‐Like Behaviors via BDNF But Not Norepinephrine
Source: Adv Sci (Weinh). 2023 Dec 28;11(10):2303503. doi: 10.1002/advs.202303503 (PMC10933643; doi:10.1002/advs.202303503)
Supplement: Supplementary file 1 — Supporting Information [file ADVS-11-2303503-s001.pdf]

ADVANCED SCIENCE
Open Access

# Supporting Information

for *Adv. Sci.*, DOI 10.1002/advs.202303503

Locus Coeruleus-Dorsolateral Septum Projections Modulate Depression-Like Behaviors via BDNF But Not Norepinephrine

*Qian Zhang, You Xue, Ke Wei, Hao Wang, Yuan Ma, Yao Wei, Yi Fan, Lei Gao, Hang Yao, Fangfang Wu, Xin Ding, Qingyu Zhang, Jianhua Ding, Yi Fan, Ming Lu and Gang Hu**

Supplementary Information for

# Locus coeruleus-dorsolateral septum projections modulate depression-like behaviors via BDNF but not norepinephrine

Qian Zhang, You Xue, Ke Wei, Hao Wang, Yuan Ma, Yao Wei, Yi Fan, Lei Gao,

Hang Yao, Fangfang Wu, Xin Ding, Qingyu Zhang, Jianhua Ding,

Yi Fan, Ming Lu, Gang Hu*

**This file includes:**

Figure S1 to S26
Table S1

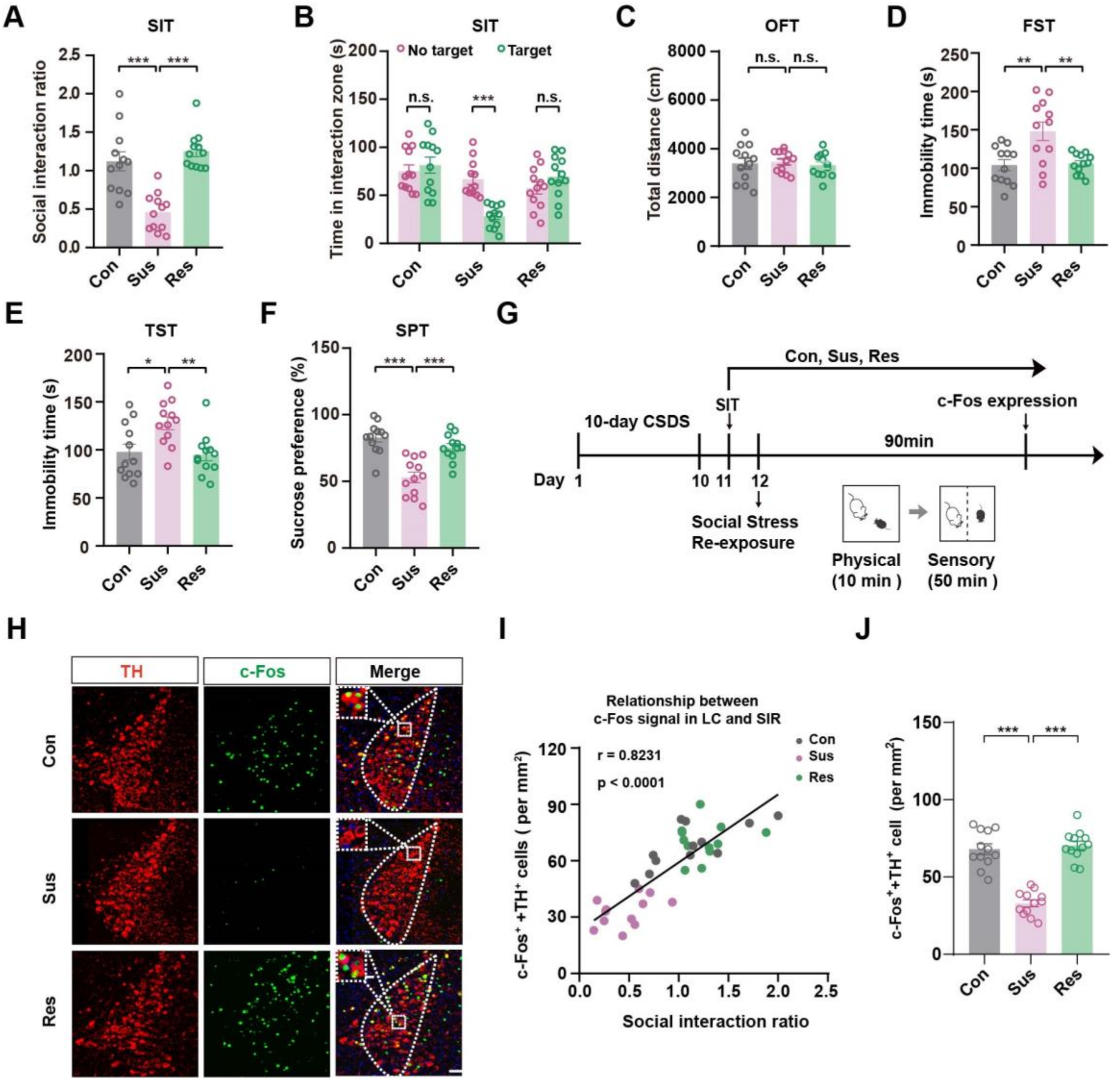


**Figure S1. CSDS markedly decrease the activity of LC$^{TH}$ neurons. (A)** Chronic stressed mice were divided into susceptible or resilient groups according to the social interaction ratio (n = 12). **(B)** Statistics of time spent in the absence or presence of social target in control, susceptible and resilient groups (n = 12). **(C)** Statistics of total distance in the open field (OFT) test in control, susceptible and resilient groups (n = 12). **(D)** Statistics of immobility time in the forced swim test (FST) in control, susceptible and resilient groups (n = 12). **(E)** Statistics of immobility time in tail suspension test (TST) in control, susceptible and resilient groups (n = 12). **(F)**

Statistics of immobility time in the sucrose preference test (SPT) in control, susceptible and resilient groups (n = 12). **(G)** Schematic of c-Fos experimental design. **(H)** The expression of c-Fos (green) and co-labeled with TH (red) neurons in LC from control, susceptible and resilient mice. Scale bars = 100 μm; Central inset, magnified view of rectangular image, Scale bars = 20 μm. **(I)** Correlation of double-positive cells in LC with social interaction ratio after CSDS (n = 12). **(J)** Statistics of c-$Fos^{+}$+$TH^{+}$ signals in control, susceptible and resilient groups (n =12). Data represent mean ± SEM. *P < 0.05, **P < 0.01, ***P < 0.001; n.s., not significant. One-way ANOVA followed by Tukey's post hoc analysis for **(A), (C-F)** and **(J)**. SIR, social interaction ratio. Two-way ANOVA followed by Bonferroni's post hoc analysis for **(B)**. Pearson correlation test for **(I)**. The statistical details can be found in Table S1, Supporting Information.

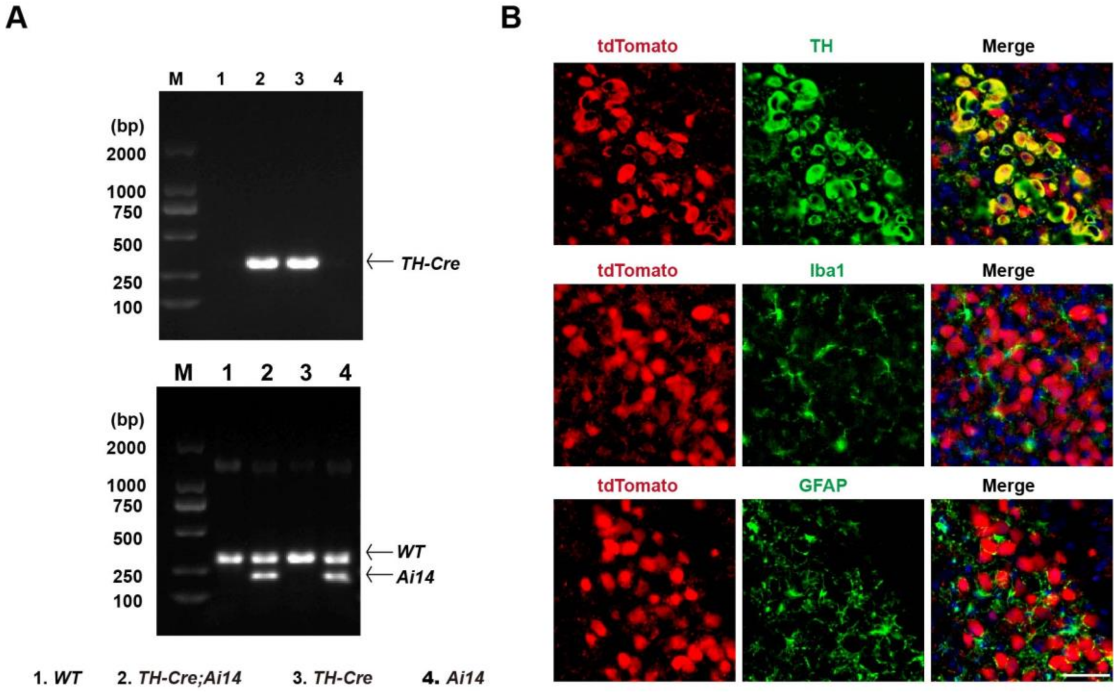


**Figure S2. Generation of *TH-Cre;Ai14* mice. (A)** PCR genotyping of *TH-Cre* and

*TH-Cre;Ai14* mice. M, maker. **(B)** Images of tdTomato expression in LC of *TH-Cre;Ai14* mice, when co-stained with TH (marker for TH neurons), IBA1 (marker for microglial cells) and GFAP (marker for astrocytes). Scale bars = 50 μm.

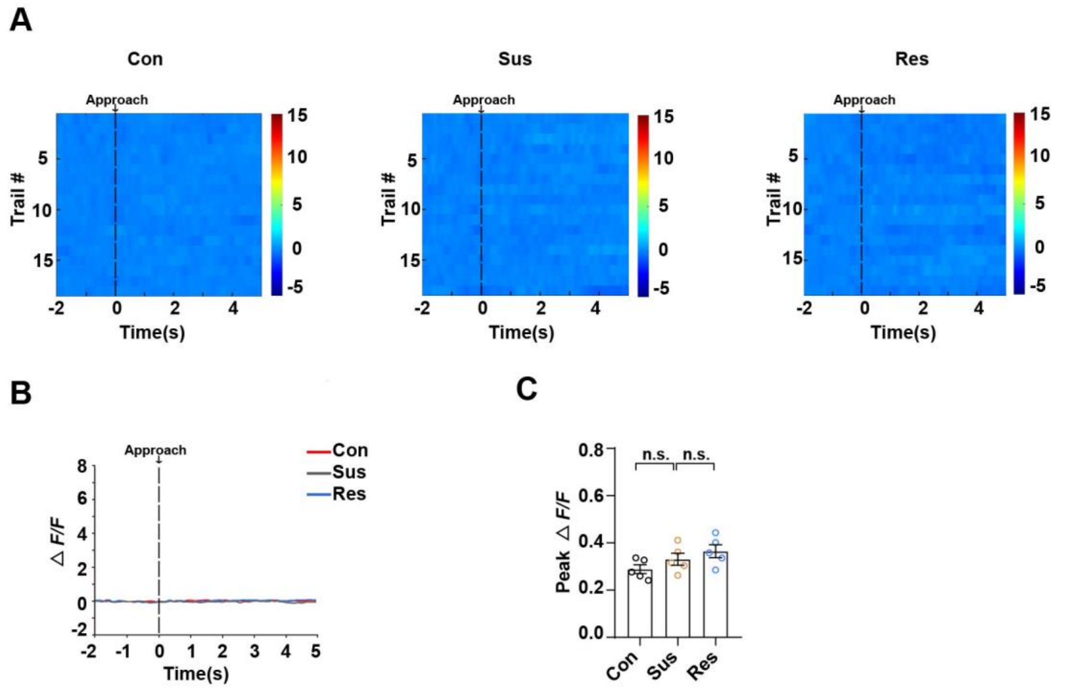


**Figure S3. $Ca^{2+}$ signals injected with EGFP in LC in *TH-Cre* mice. (A)** Heatmaps of $Ca^{2+}$ transients evoked by approaching an unfamiliar CD1 mouse from control, susceptible and resilient mice (trial =18, mice = 5). EGFP was expressed in the $LC^{TH}$ neurons instead of GCamp6s. Color scales at the right referring to the ΔF/F. **(B)** The average plots of $Ca^{2+}$ response from control, susceptible and resilient mice in EGFP positive neurons. **(C)** Statistics of the peak $Ca^{2+}$ activities at the onset of approaching an unfamiliar CD1 mouse from control, susceptible and resilient mice (n = 5). Data represent mean ± SEM. *P < 0.05, **P < 0.01, ***P < 0.001; n.s., not significant. One-way ANOVA followed by Tukey's post hoc analysis for **(C)**. The statistical details can be found in Table S1, Supporting Information.

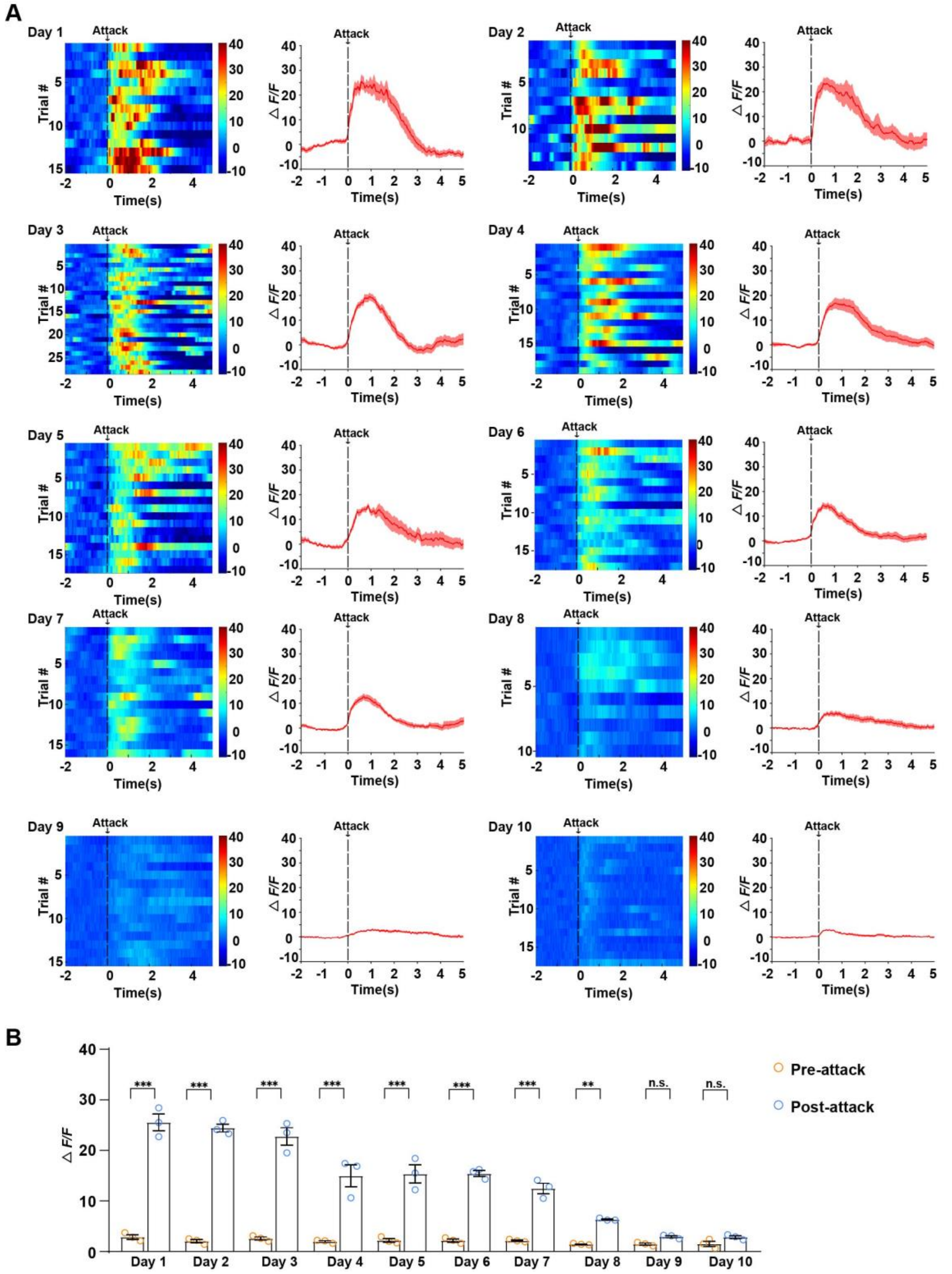


**Figure S4. The changes in $Ca^{2+}$ signals in $LC^{TH}$ neurons during 10-day CSDS in Sus mice. (A)** Heatmaps of $Ca^{2+}$ transients (left) and the average plots of $Ca^{2+}$ transients (right) in $LC^{TH}$ neurons before and after attacks by an unfamiliar CD1 mouse on susceptible mice from Day 1 to Day 10, (Day 1, trial = 15; Day 2, trial = 14;

Day 3, trial = 28; Day 4, trial = 19; Day 5, trial = 17; Day 6, trial = 17; Day 7, trial = 16; Day 8, trial = 10; Day 9, trial = 15; Day 10, trial = 17; mice = 3). Color scales at the right referring to the ΔF/F. **(B)** Statistics of the peak $Ca^{2+}$ activities before and after attacks by an unfamiliar CD1 mouse on susceptible mice from Day 1 to Day 10 (n = 3). Data represent mean ± SEM. *P < 0.05, **P < 0.01, ***P < 0.001; n.s., not significant. Two-way ANOVA followed by Tukey's post hoc analysis for **(B).** CSDS, chronic social defeat stress; Sus, susceptible. The statistical details can be found in Table S1, Supporting Information.

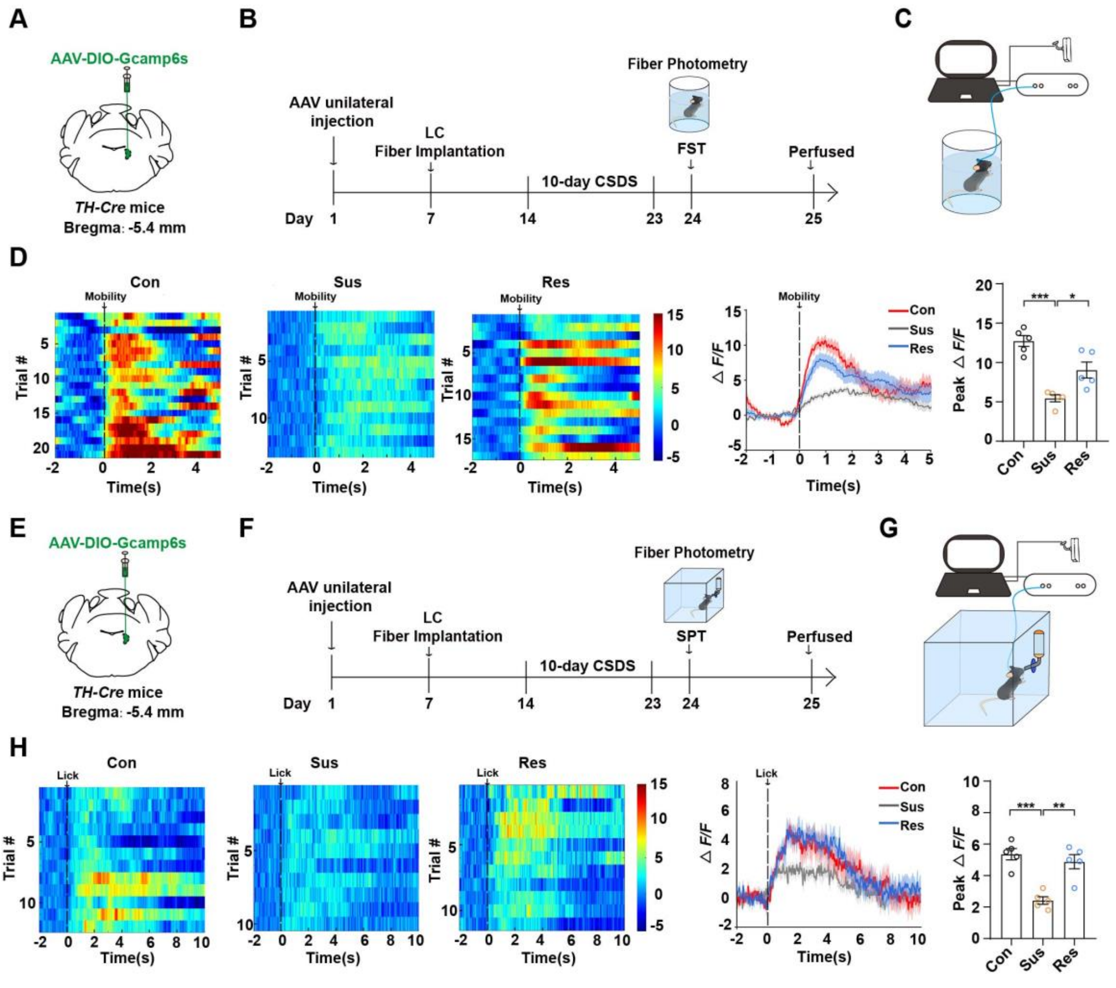


**Figure S5. The $Ca^{2+}$ signals in $LC^{TH}$ neurons in FST and SPT in Con, Sus and Res mice**. **(A)** Schematic representation of the virus injection. **(B)** Experimental

scheme of fiber photometry recordings in control and CSDS-treated mice in FST. CSDS, chronic social defeat stress; FST, forced swim test. **(C)** Schematic of the fiber photometry setup. **(D)** Heatmaps of $Ca^{2+}$ transients (left), average plots of $Ca^{2+}$ response (middle), and peak $Ca^{2+}$ activities (right) evoked by the onset of mobility in $LC^{TH}$ neurons in Con, Sus and Res mice (Con, trial =21, mice = 5; Sus, trial = 13, mice = 5; Res, trial = 17, mice = 5). Con, control; Sus, susceptible; Res, resilient. **(E)** Schematic representation of the virus injection. **(F)** Experimental scheme of fiber photometry recordings in control and CSDS-treated mice in SPT. SPT, sucrose preference test. **(G)** Schematic of the fiber photometry setup. **(H)** Heatmaps of $Ca^{2+}$ transients (left), average plots of $Ca^{2+}$ response (middle), and peak $Ca^{2+}$ activities (right) evoked by the onset of sucrose lick in the $LC^{TH}$ neurons in control and CSDS-treated mice (Con, trial =13, mice = 5; Sus, trial = 10, mice = 5; Res, trial = 11, mice = 5). Data represent mean ± SEM. *P < 0.05, **P < 0.01, ***P < 0.001; n.s., not significant. One-way ANOVA followed by Tukey's post hoc analysis for **(D)** and **(H)**. The statistical details can be found in Table S1, Supporting Information.

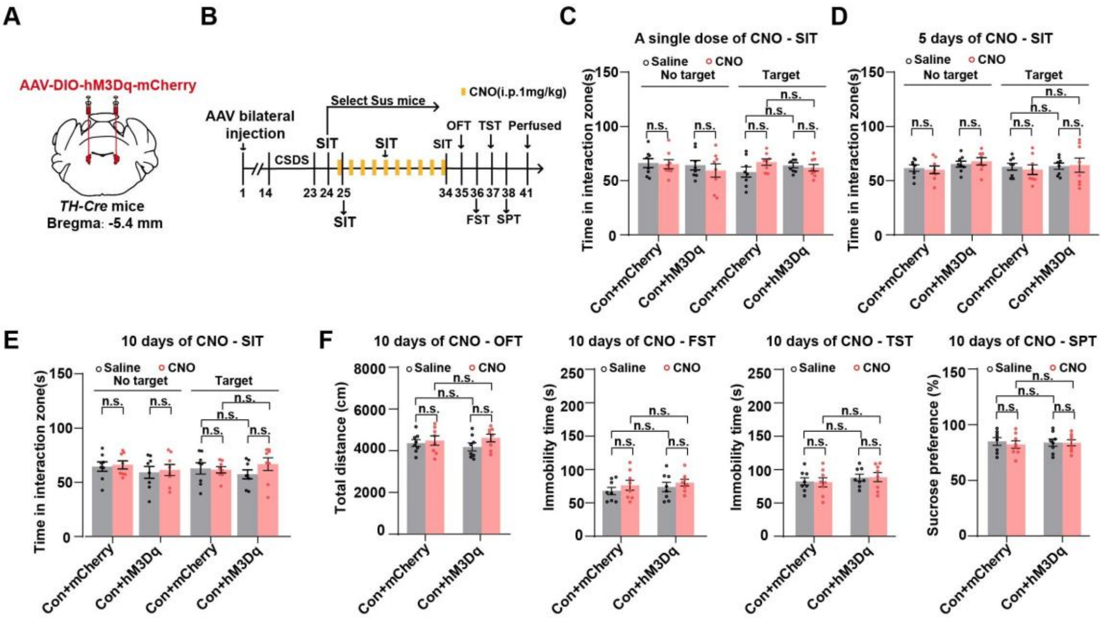


**Figure S6. Chemogenetic activation of LC$^{TH}$ neurons has no effects on depression-like behaviors in control mice. (A)** Schematic representation of virus injection. **(B)** Schematic of the experimental design. **(C-E)** Statistical analysis of time spent in the social interaction zone in the SIT after a single dose of CNO or saline **(C)**, 5 days of repeated CNO or saline **(D)**, and 10 days of repeated CNO or saline treatment in different groups **(E)** Con, control; CNO, clozapine (n = 8). **(F)** Locomotor activity in the OFT, immobility time in the FST and TST, and sucrose preference in the SPT after 10 days of repeated saline or CNO stimulation in different groups (n = 8). Data represented as mean ± SEM. *P < 0.05, **P < 0.01, ***P < 0.001; n.s., not significant. Two-way ANOVA followed by Bonferroni's post hoc analysis for **(C-F)**. The statistical details can be found in Table S1, Supporting Information.

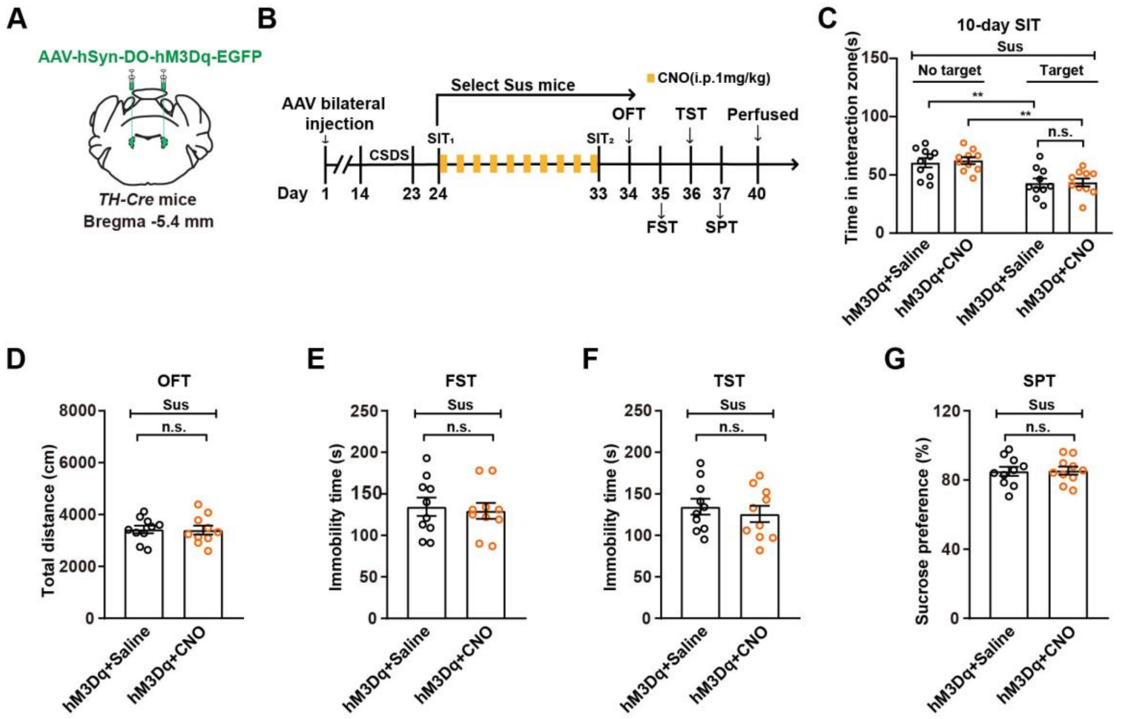


**Figure S7. Activation of non-TH neurons in LC failed to alleviate the depression-like behaviors induced by CSDS. (A)** Schematic of bilateral virus infection in the non-TH neurons in the LC. **(B)** Schematic of the experimental design. **(C)** Results of time spent in the absence or presence of social target in control, susceptible and resilient groups **(C)**, locomotor activity in OFT **(D)**, immobility time in FST **(E)**, TST **(F)** and sucrose preference **(G)** in SPT after 10 days of repeated CNO or Saline treatment in susceptible mice (n =10). Data represent mean ± SEM. *P < 0.05, **P < 0.01, ***P < 0.001; n.s., not significant. Unpaired two-tailed Student's t test for **(D-G)**. Two-way ANOVA followed by Bonferroni's post hoc analysis for **(C)**. The statistical details can be found in Table S1, Supporting Information.

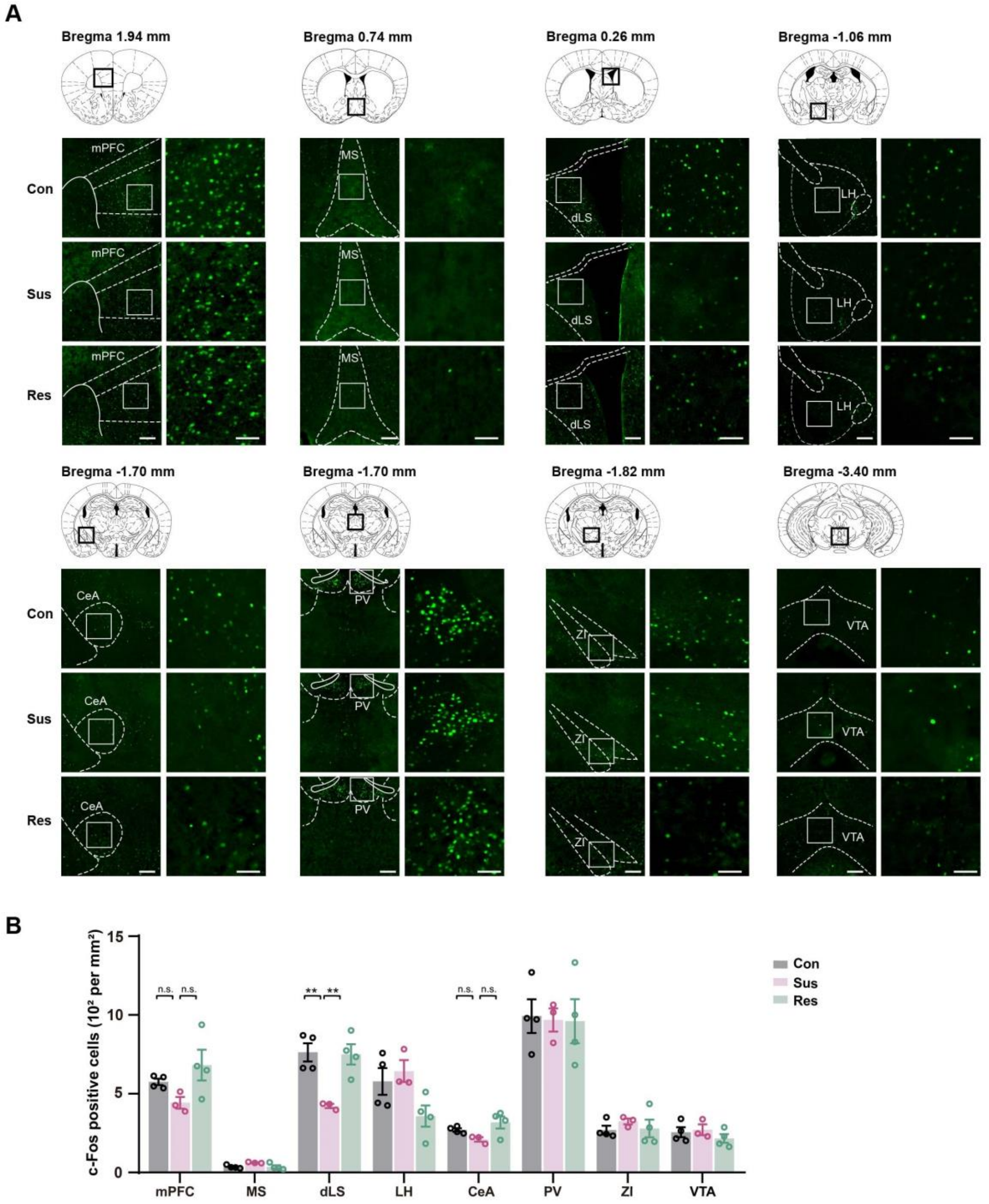


**Figure S8. The altered neuronal activity activated by social interaction test in downstream brain areas of projection in the $LC^{TH}$ neurons in Con, Sus and Res mice. (A)** Example of c-Fos immunostaining in downstream brain regions of $LC^{TH}$ neurons in Con, Sus and Res mice. Scale bars in left are 100 μm, magnified view of rectangular image, Scale bars = 50 μm. **(B)** Counts of c-$Fos^{+}$ cells in different

downstream brain regions after CSDS in control (n = 4), susceptible mice (n = 3) and resilient mice (n = 4). Data represent mean ± SEM. *P < 0.05, **P < 0.01, ***P < 0.001; n.s., not significant. One-way ANOVA followed by Tukey's post hoc analysis for **(B).** mPFC, medial prefrontal cortex; MS, medial septal nucleus; dLS, dorsolateral septum; LH, lateral hypothalamic area; CeA, central nucleus of the amygdala; PV, paraventricular thalamic nucleus; ZI, zona incerta; VTA, ventral tegmental area. The statistical details can be found in Table S1, Supporting Information.

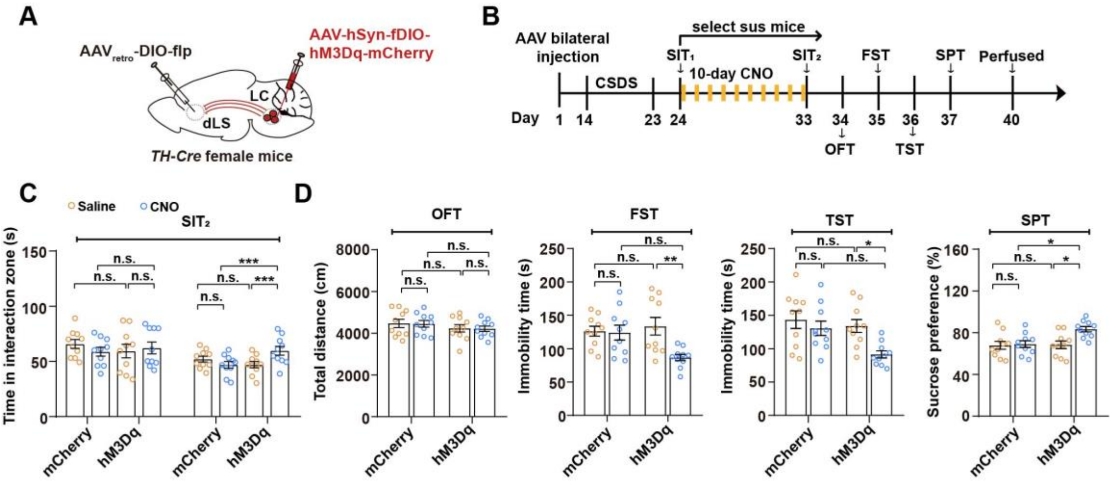


**Figure S9. Manipulating the LC$^{TH}$-dLS circuit regulates female mice depression-like behaviors. (A)** Schematic of virus injection to express hM3Dq in LC$^{TH}$-dLS circuit in *TH-Cre* female mice. **(B)** Experimental timeline of 10-day repeated chemogenetic activation of the LC$^{TH}$-dLS circuit in susceptible female mice following 10-day CSDS. **(C)** Results of time spent in the absence or presence of social target in susceptible female mice expressing either mCherry or hM3Dq virus in LC$^{TH}$-dLS circuit (n =10). **(D)** Results of locomotor activity in OFT, immobility time

in FST, TST and sucrose preference in SPT in susceptible female mice expressing either mCherry or hM3Dq virus in LC$^{TH}$-dLS circuit (n =10). Data represent mean ± SEM. *P < 0.05, **P < 0.01, ***P < 0.001; n.s., not significant. Two-way ANOVA followed by Bonferroni's post hoc analysis for **(C-D)**. The statistical details can be found in Table S1, Supporting Information.

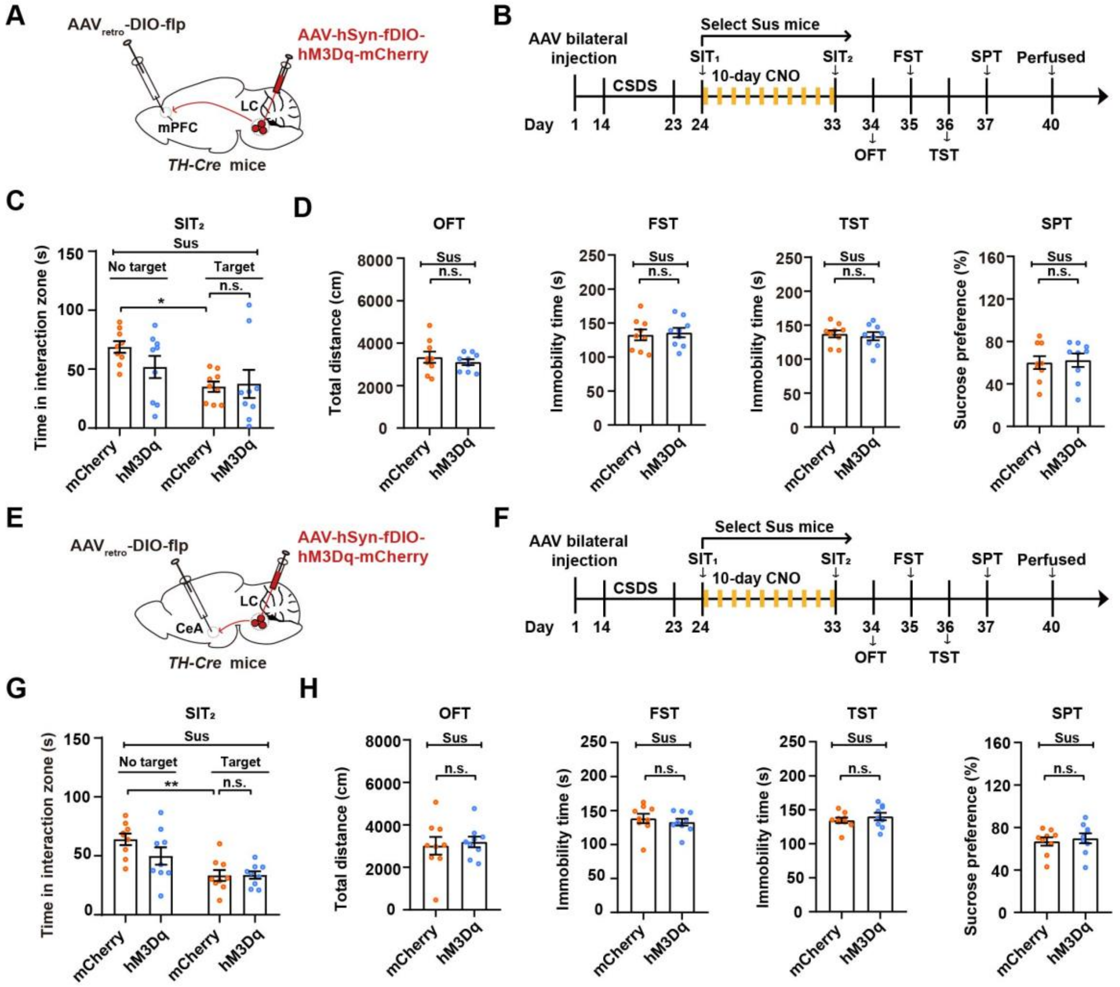


**Figure S10. Chemogenetic activation of LC$^{TH}$-mPFC or LC$^{TH}$-CeA circuit has no effects on depression-like behaviors. (A)** Schematic of virus injection to express hM3Dq in LC$^{TH}$-mPFC circuit in *TH-Cre* mice. **(B)** Experimental timeline of 10-day repeated chemogenetic activation of the LC$^{TH-}$mPFC circuit in susceptible mice following 10-day CSDS. **(C)** Results of time spent in the absence or presence of

social target in susceptible mice expressing either mCherry or hM3Dq virus in $LC^{TH}$-mPFC circuit (n = 9). **(D)** Results of locomotor activity in OFT, immobility time in FST, TST and sucrose preference in SPT in susceptible mice expressing either mCherry or hM3Dq virus in $LC^{TH}$-mPFC circuit (n = 9). **(E)** Schematic of virus injection to express hM3Dq in $LC^{TH}$-CeA circuit in *TH-Cre* mice. **(F)** Paradigms of 10-day repeated chemogenetic activation of the $LC^{TH}$-CeA circuit in susceptible mice following 10-day CSDS. **(G)** Results of time spent in the absence or presence of social target in susceptible mice expressing either mCherry or hM3Dq virus in $LC^{TH}$-CeA circuit (n = 9). **(H)** Results of locomotor activity in OFT, immobility time in FST, TST and sucrose preference in SPT in susceptible mice expressing either mCherry or hM3Dq virus in $LC^{TH}$-CeA circuit (n = 9). Data represent mean ± SEM. *P < 0.05, **P < 0.01, ***P < 0.001; n.s., not significant. Unpaired two-tailed Student's t test for **(D, H)**. Two-way ANOVA followed by Bonferroni's post hoc analysis for **(C, G)**. The statistical details can be found in Table S1, Supporting Information.

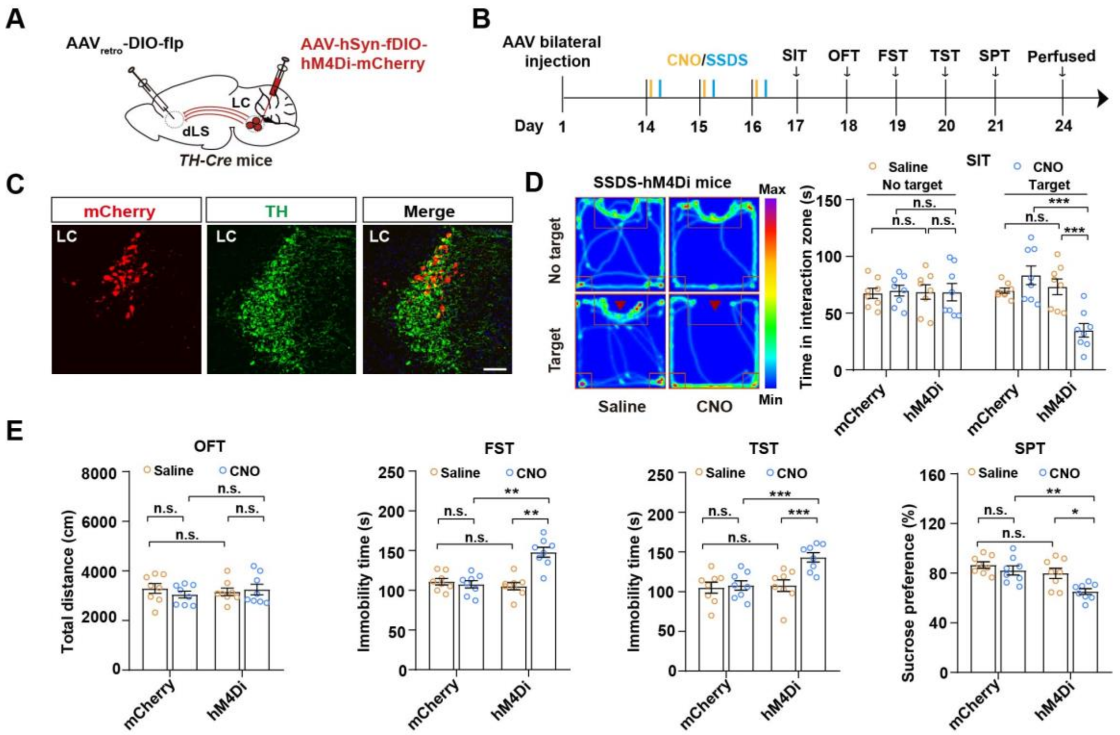


**Figure S11. Chemogenetic inhibition of $LC^{TH}$-dLS circuit could trigger SSDS-induced depression-like behaviors. (A)** Schematic of viral infection. **(B)** Experimental scheme. **(C)** Representative image of LC injection sites. Scale bar = 100 µm. **(D)** Representative social interaction tracks and the time spent in the interaction zone in the SIT in different groups (n = 8). SSDS, subthreshold social defeat stress. **(E)** Results of locomotor activity in the OFT, immobility time in the FST and TST, sucrose preference in the SPT in susceptible mice expressing the mCherry or hM4Di virus (n = 8). The data represent the mean ± SEM. *P < 0.05, **P < 0.01, ***P < 0.001; n.s., not significant. Two-way ANOVA followed by Bonferroni's post hoc analysis for **(D, E)**. The statistical details can be found in Table S1, Supporting Information.

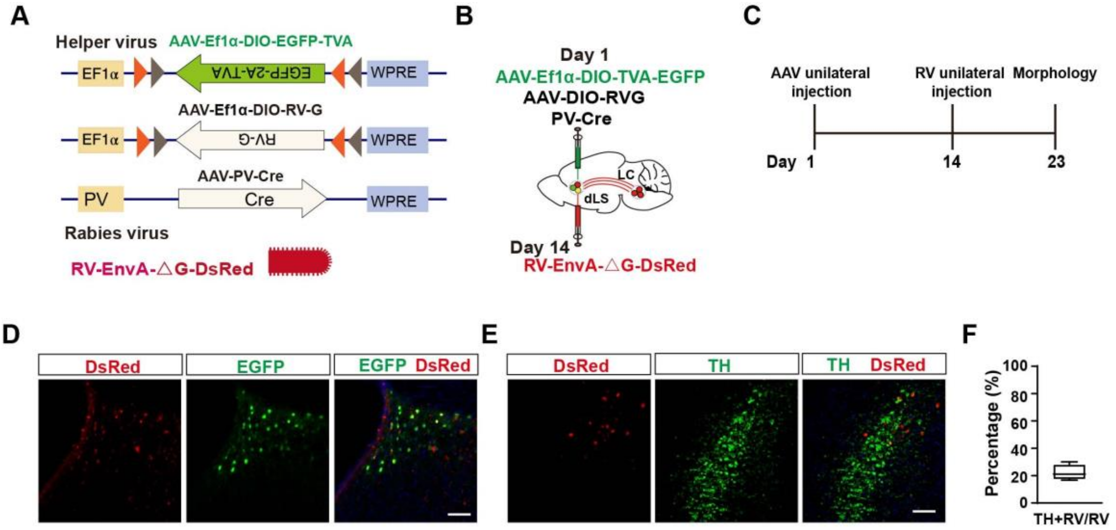


**Figure S12. Monosynaptic rabies retrograde tracing of parvalbumin (PV) neurons in dLS. (A)** Schematic representation. **(B)** dLS was injected with AAV-DIO-TVA-EGFP, AAV-DIO-RVG and AAV-PV-Cre in mice and 3 weeks later RV-EnvA-ΔG-DsRed was subsequently injected into the dLS of the same site. **(C)** Experimental scheme of retrograde tracing. **(D)** Representative images showing the DsRed/EGFP double-stained cells were observed in the dLS PV neurons. Scale bar = 100 μm. **(E)** DsRed signals traced from the dLS PV neurons were co-localized with TH immunofluorescence in the LC. Scale bar = 100 μm. **(F)** Percentage of DsRed-labeled neurons that expressed TH in the LC (n = 3). PV, parvalbumin.

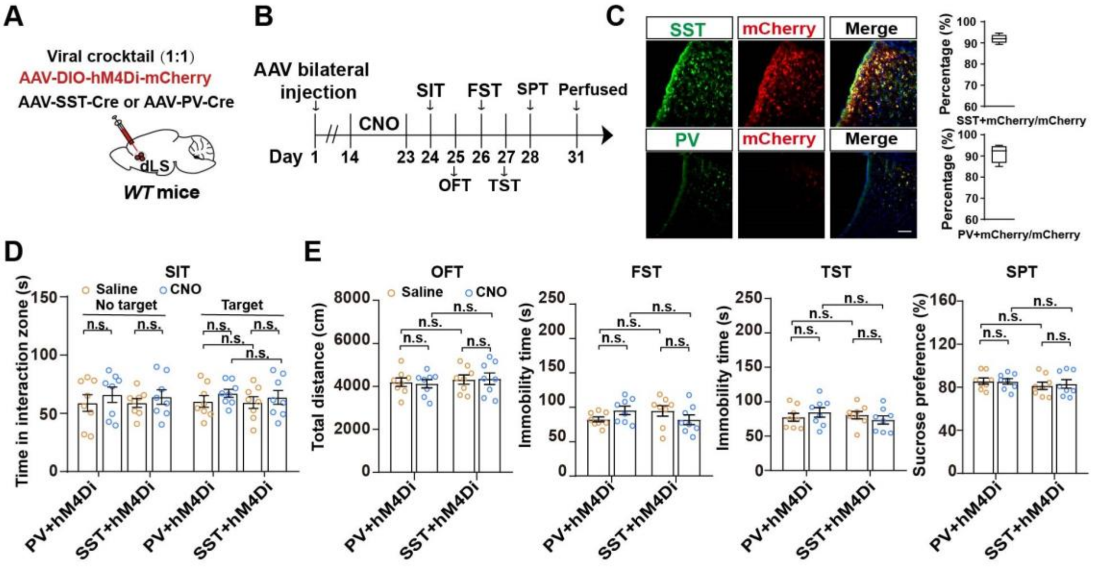

**Figure S13. Direct inhibition of PV or SST neurons has no effect on depression-like behaviors. (A)** Schematic of viral infection. **(B)** Experimental scheme. **(C)** Representative images and quantification of co-expression of SST antibody or PV antibody (green) and AAV-DIO-hM4Di-mCherry+AAV-PV-Cre or AAV-SST-Cre (red) in dLS of WT mice (n = 3). Scale bar = 100 μm. **(D)** The time spent in the interaction zone in the SIT in different groups (n = 8). **(E)** Results of locomotor activity in the OFT, immobility time in the FST and TST, sucrose preference in the SPT in *WT* mice expressing the PV or SST virus (n = 8). PV, parvalbumin; SST, somatostatin. The data represent the mean ± SEM. *P < 0.05, **P < 0.01, ***P < 0.001; n.s., not significant. Two-way ANOVA followed by Bonferroni's post hoc analysis for **(D, E)**. The statistical details can be found in Table S1, Supporting Information.

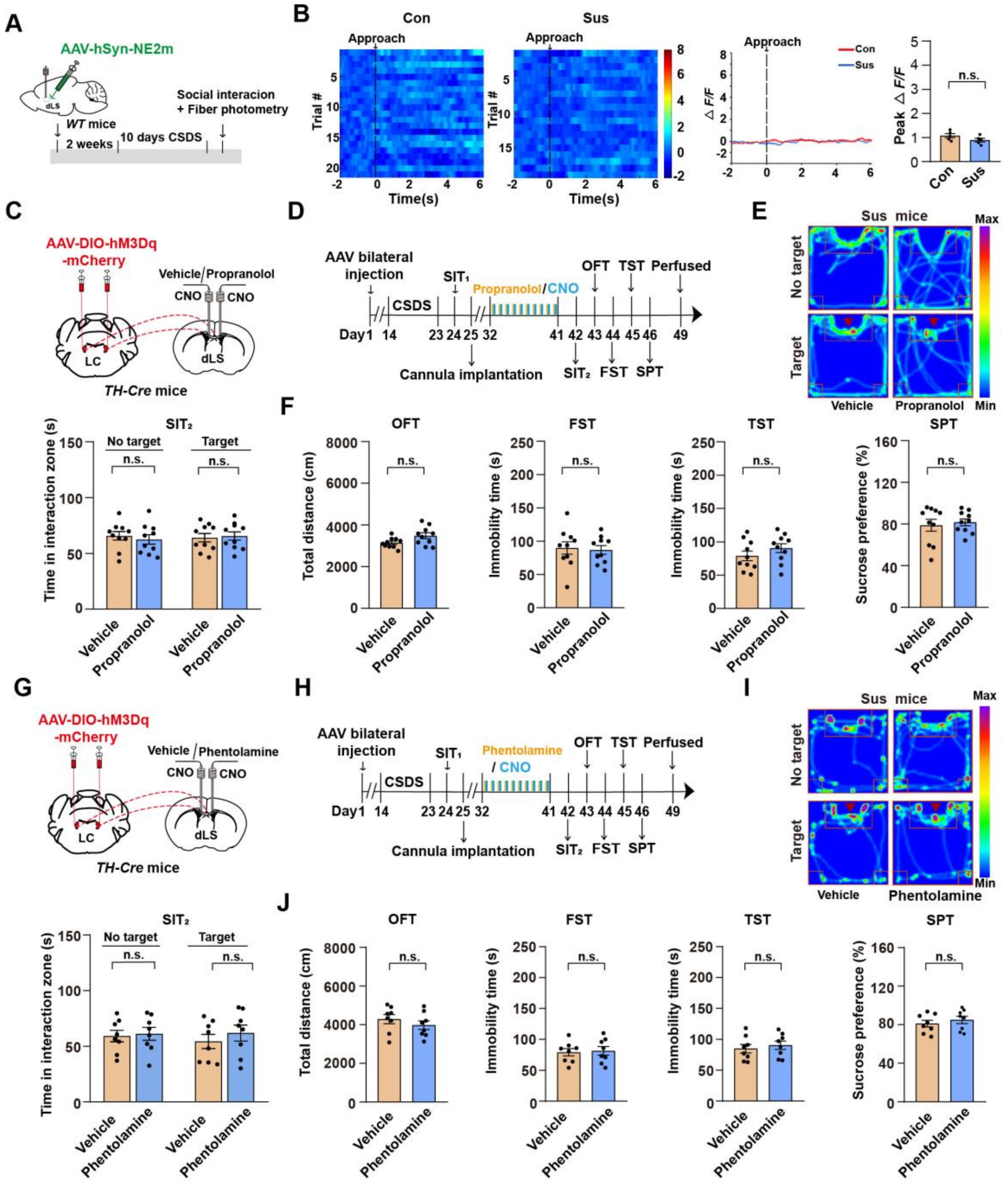


**Figure S14. LC$^{TH}$-dLS circuit modulation of depression-like behavior is not NE dependent. (A)** Mice with CSDS treatment received AAVs carrying the GRABNE sensor NE2m and the social interaction test with simultaneous fiber photometry. **(B)** Heatmaps of NE2m transients (left), average plots of NE2m response (middle), peak NE2m activities (right) evoked by approaching an unfamiliar CD1 mouse in the Con and Sus mice (Con, trial =21, mice = 5; Sus, trial = 19, mice = 5). **(C)** Schematic of the injection strategy. **(D)** Timeline of the experiments performed. **(E)** Representative social interaction tracks of mice infused with vehicle or propranolol and the time

spent in the interaction zone in the SIT in different groups (n = 10). **(F)** Results of locomotor activity and depressive-like behaviors in susceptible mice infused with vehicle and propranolol (n = 10). **(G)** Schematic of the injection strategy. **(H)** Timeline of the experiments performed. **(I)** Representative social interaction tracks of mice infused with vehicle or phentolamine and the time spent in the interaction zone in the SIT in different groups (n = 8). **(J)** Results of locomotor activity and depressive-like behaviors in susceptible mice infused with vehicle and phentolamine (n = 8). The data represent the mean ± SEM. *P < 0.05, **P < 0.01, ***P < 0.001; n.s., not significant. Unpaired two-tailed Student's t test for **(B), (F)** and **(J).** Two-way ANOVA followed by Bonferroni's post hoc analysis for **(E)** and **(I)**. The statistical details can be found in Table S1, Supporting Information.

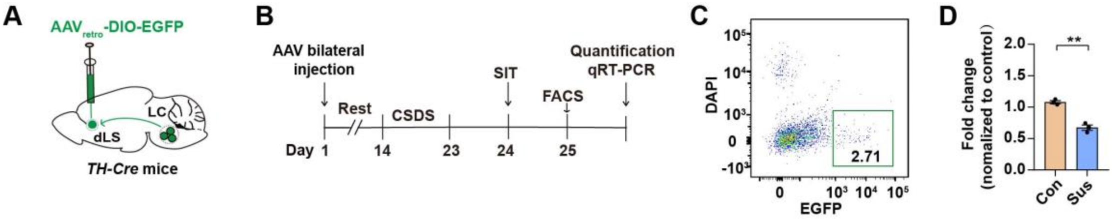


**Figure S15. The mRNA levels of BDNF were significantly decreased in LC$^{TH}$-dLS circuit in Sus mice.** **(A)** Schematic of virus injection. **(B)** Experimental scheme of analysis of LC$^{TH}$-dLS neurons sorted by FACS in the LC following CSDS. FACS, fluorescence-activated cell sorting. **(C)** Representative FACS image of sorted the LC$^{TH}$-dLS neurons. **(D)** The levels of BDNF mRNA in the LC$^{TH}$-dLS circuit from control and susceptible mice (n = 3). The data represent the mean ± SEM. **P < 0.01. Unpaired two-tailed Student's t test for **(D).** The statistical details can be found in Table S1, Supporting Information.

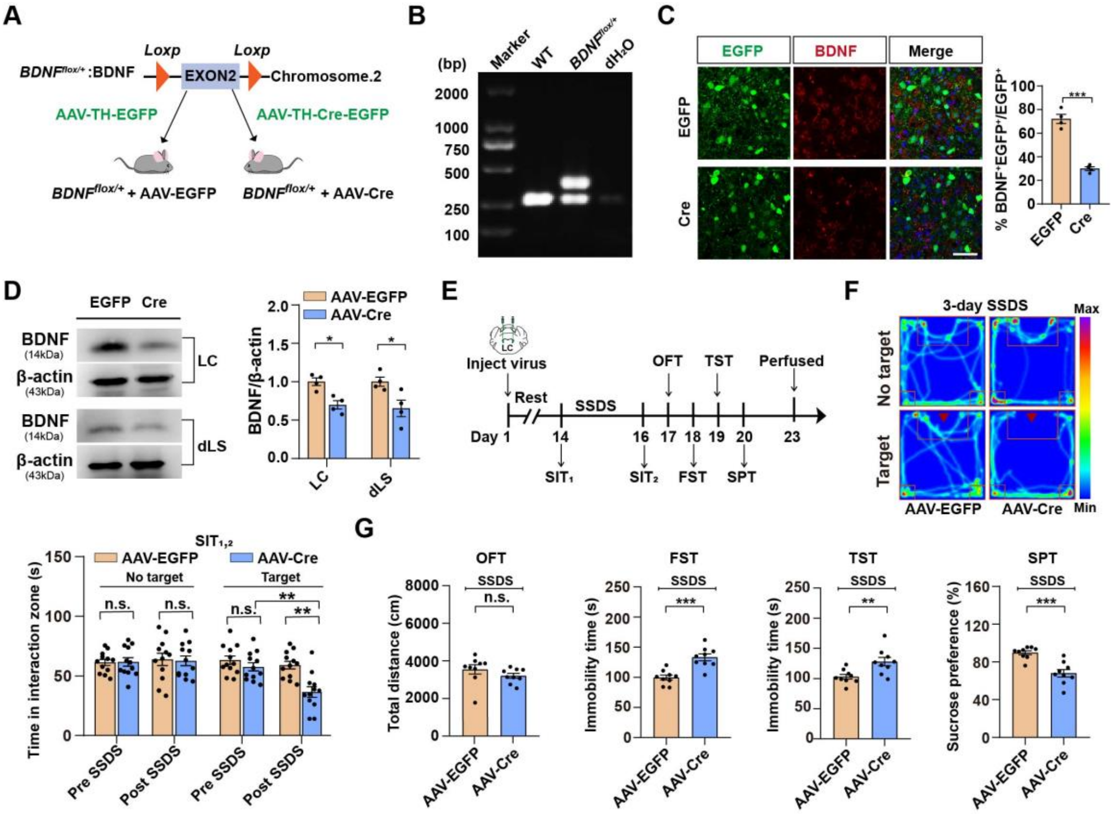


**Figure S16. The expression of BDNF in LC was reduced in the BDNF$^{flox/+}$ mice after injection of AAV-TH-Cre-EGFP virus. (A)** Schematic of *BDNF$^{flox/+}$* mice injected with AAV-TH-Cre-EGFP (AAV-Cre) and AAV-TH-EGFP (AAV-EGFP) in the LC. **(B)** PCR genotyping of *BDNF$^{flox/+}$* mice. **(C)** Representative images and quantification of co-expression of BDNF (red) and AAV-TH-Cre-EGFP (green) in LC of *BDNF$^{flox/+}$* mice (n = 4). Scale bar = 50 μm. **(D)** Western blot analysis of BDNF in the LC and dLS of *BDNF$^{flox/+}$* mice injected with AAV-TH-Cre-EGFP or AAV-TH-EGFP (n = 4). **(E)** Experimental scheme of behavioural studies in *BDNF$^{flox/+}$* mice. All the *BDNF$^{flox/+}$* mice were injected with either AAV-Cre or the control virus AAV-EGFP in LC to select loss the BDNF in TH neurons of LC. **(F)** Representative social interaction tracks of AAV-EGFP and AAV-Cre mice following

3-day SSDS (left). Time in the interaction zone in the SIT between AAV-EGFP and AAV-Cre mice following 3-day SSDS (right) (n = 12). **(G)** Results of locomotor activity in OFT, immobility time in FST and TST, sucrose preference in SPT between AAV-EGFP and AAV-Cre mice (n = 9). Data represent mean ± SEM. ***P < 0.001. Unpaired two-tailed Student's t test for **(C, G).** One-way ANOVA followed by Tukey's post hoc analysis for **(D).** Two-way ANOVA followed by Bonferroni's post hoc analysis for **(F).** The statistical details can be found in Table S1, Supporting Information.

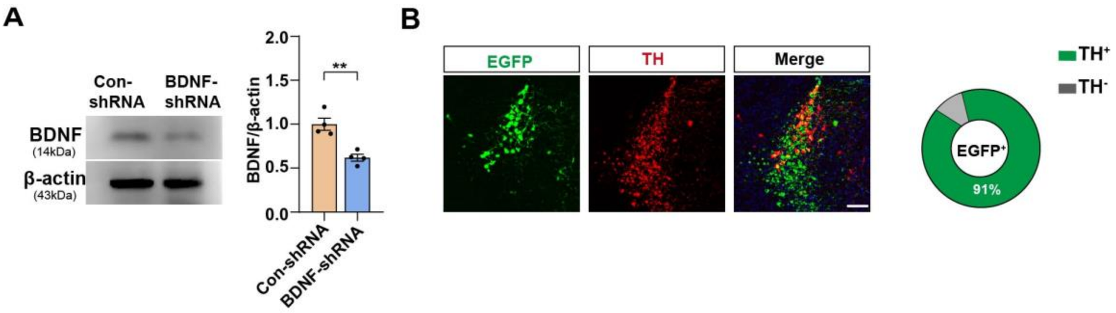


**Figure S17. The knockdown efficiency of BDNF in the LC$^{TH}$-dLS circuit. (A)** Western blot analysis of BDNF protein in the tissue of LC between control-shRNA and BDNF-shRNA group (n = 4). **(B)** Representative images and quantification of co-expression of BDNF-shRNA-EGFP (green) and TH (red) in LC with AAV$_{retro}$-TH-Cre injected in dLS and AAV-DIO-BDNF-shRNA-EGFP injected in LC. Scale bar = 100 μm. The data represent the mean ± SEM. **P < 0.01. Unpaired two-tailed Student's t test for **(A).** The statistical details can be found in Table S1, Supporting Information.

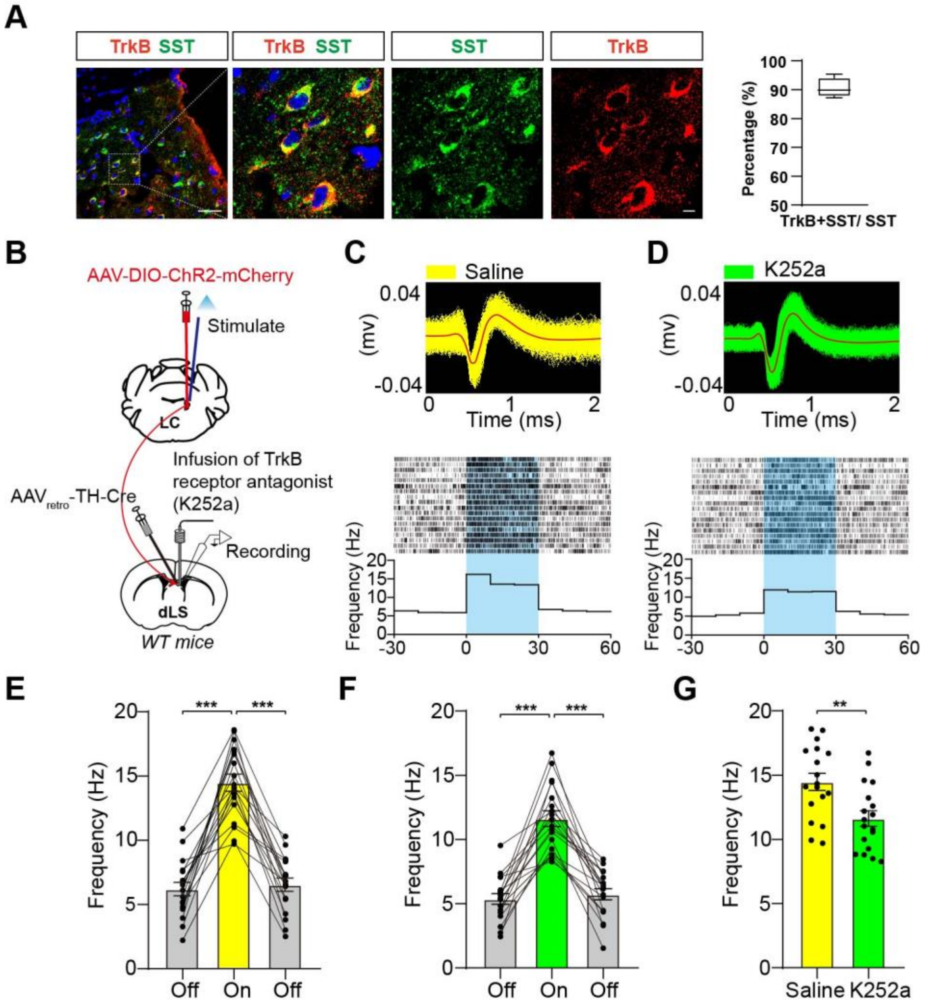


**Figure S18. Excitatory projection of LC to dLS is impaired by TrkB receptor antagonists (K252a). (A)** Representative images and quantification of co-expression of SST antibody (green) and TrkB antibody (red) in dLS. Scale bar = 100 μm (left); Scale bar = 100 μm (right). **(B)** Optogenetics virus injecting strategies from LC to dLS, cannulae and in vivo electrophysiological recording paradigm. **(C-D)** Representative normalized spikes waveforms (upper) and raster plots (lower) of dLS neurons in response to activation of dLS projecting $LC^{TH}$ neurons under saline or K252a condition. **(E-F)** Firing rate of neurons upon ChR2-optogenetic stimulation for **(C)** and **(D)**. **(G)** Firing rate difference of dLS neurons in response to activation of dLS projecting $LC^{TH}$ neurons under saline or K252a condition (neurons = 18; mice = 4). The data represent the mean ± SEM. **P < 0.01, ***P < 0.001. One-way repeated-measures ANOVA followed by Dunn's multiple comparisons test for (**E-F**).

Unpaired two-tailed Student's t test for **(G).** The statistical details can be found in Table S1, Supporting Information.

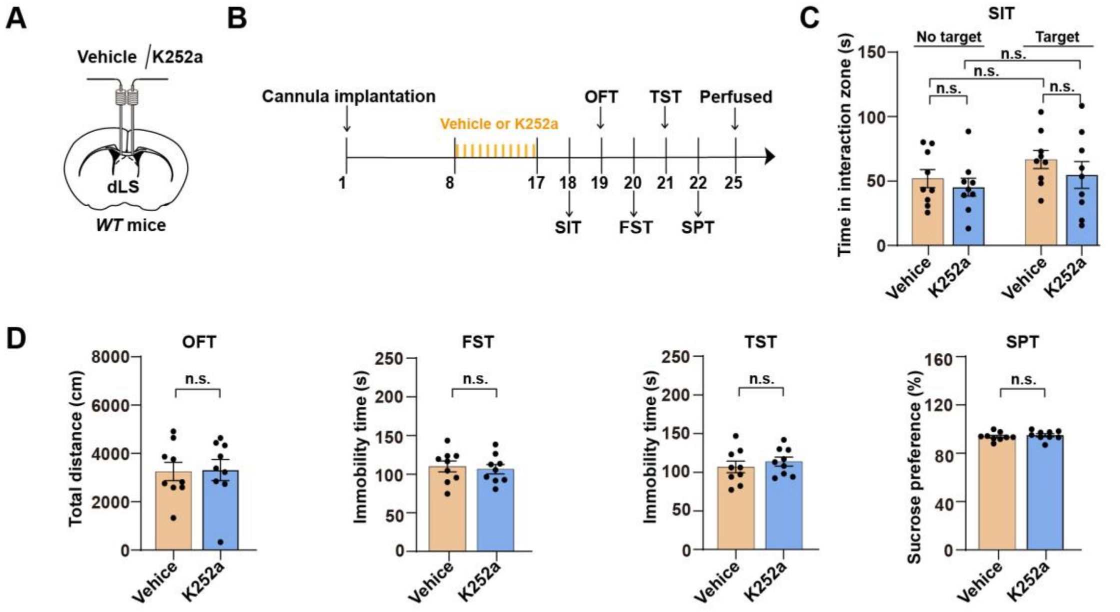


**Figure S19. Direct inhibition of TrkB receptors has no effect on depression-like behaviors. (A)** Schematic of drug injection. **(B)** Timeline of the experiments performed. **(C)** The time spent in the interaction zone in the SIT in different groups (n = 9). **(D)** Results of locomotor activity and depression-like behaviors in *WT* mice infused with vehicle or k252a (n = 9). The data represent the mean ± SEM. n.s., not significant. Unpaired two-tailed Student's t test for (**D**). Two-way ANOVA followed by Bonferroni's post hoc analysis for (**C**). The statistical details can be found in Table S1, Supporting Information.

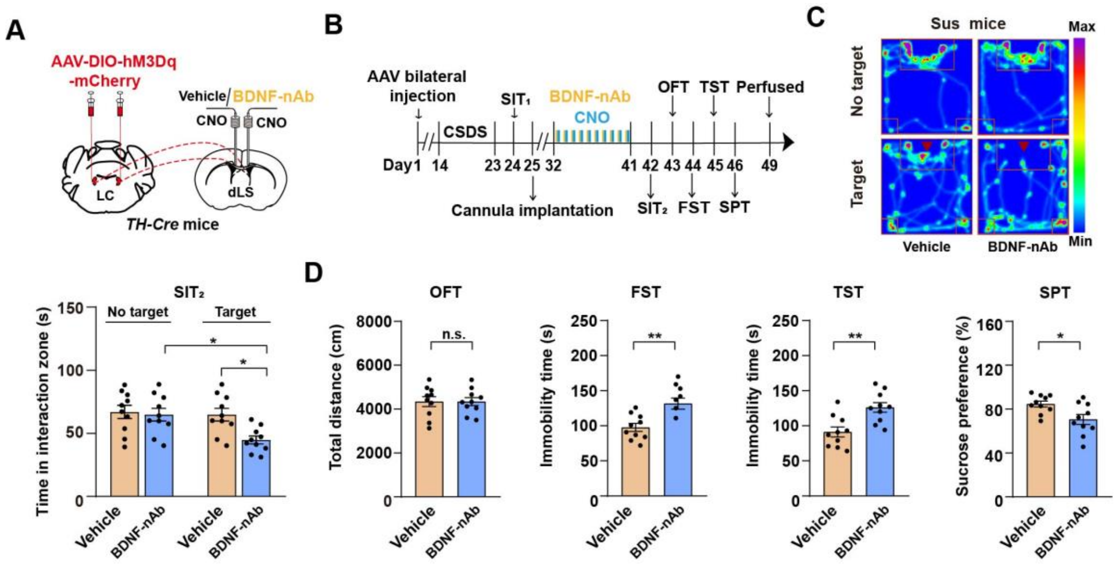


**Figure S20. Infusion of anti-BDNF neutralizing antibody into the dLS abolished the antidepressant-like effects produced by chemogenetic activation of LC$^{TH}$-dLS circuit.** **(A)** Schematic representation of the viral infection in *TH-Cre* mice. **(B)** Timeline of experiments. **(C)** Time spent in the interaction zone in the SIT in mice infused with vehicle or anti-BDNF neutralizing antibody (BDNF-nAb) (n = 10). BDNF-nAb, BDNF neutralizing antibody. **(D)** Locomotor activity and depression-like behaviors in *TH-Cre* mice infused with vehicle or BDNF-nAb (n = 10). Data represented as mean ± SEM. *P < 0.05, **P < 0.01, ***P < 0.001; n.s., not significant. Unpaired two-tailed Student's t-tests for **(D)**. Data were analyzed by two-way ANOVA followed by Bonferroni's post hoc analysis for **(C)**. The statistical details can be found in Table S1, Supporting Information.

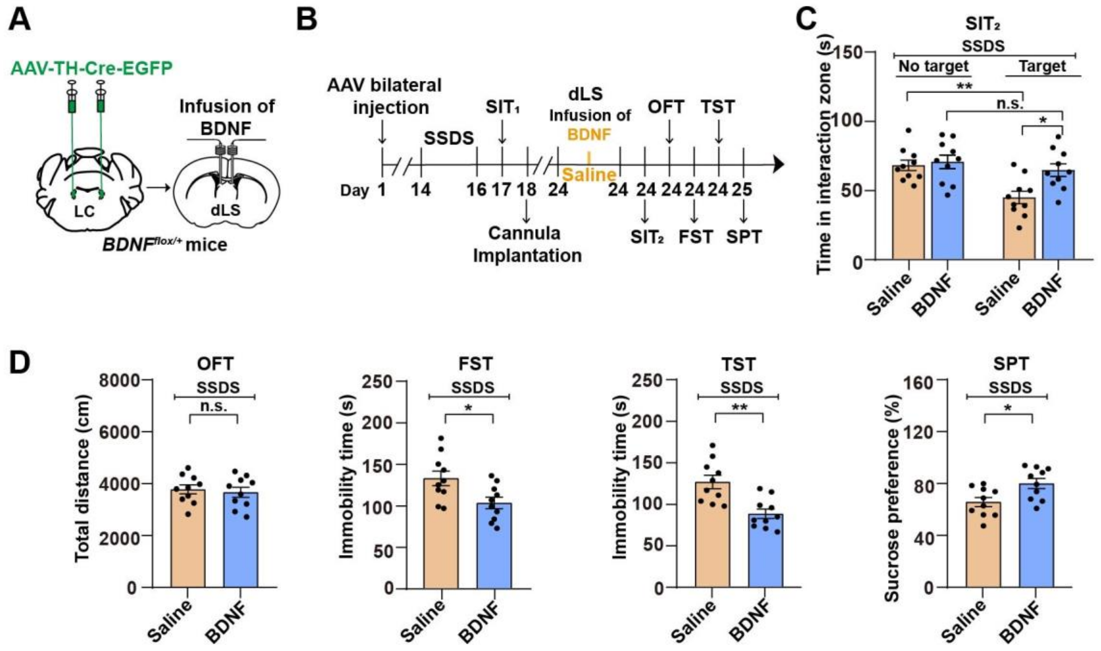


**Figure S21. Single intra-dLS infusion of BDNF produces antidepressant-like effects.** **(A)** Schematic representation of the viral infection in *BDNF*$^{flox/+}$ mice. **(B)** Timeline of experiments. **(C)** Time spent in the interaction zone in the SIT in different groups (n = 10). **(D)** Locomotor activity and depression-like behaviors in *BDNF*$^{flox/+}$ mice after 3 days of SSDS and infused with saline or BDNF (n = 10). Data represented as mean ± SEM. *P < 0.05, **P < 0.01; n.s., not significant. Unpaired two-tailed Student's t-tests for **(D)**. Data were analyzed by two-way ANOVA followed by Bonferroni's post hoc analysis for **(C)**. The statistical details can be found in Table S1, Supporting Information.

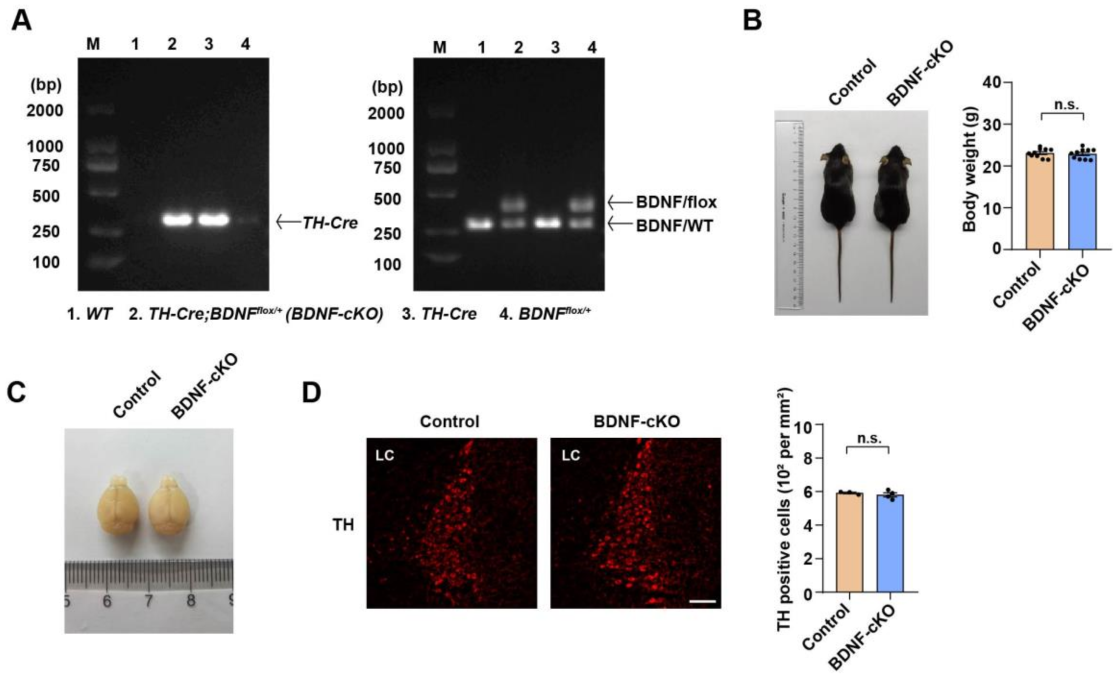


**Figure S22. Generation of *TH-Cre;BDNF$^{flox/+}$* (*BDNF-cKO*) mice. (A)** PCR genotyping of *TH-Cre;BDNF$^{flox/+}$*(*BDNF-cKO*) mice. M, maker. **(B)** Body size and weight for adult *BDNF-cKO* and control mice. cKO, conditional knockout (n = 10). **(C)** Brain size for adult *BDNF-cKO* and control mice. **(D)** Representative images and quantification of TH-positive neurons for adult *BDNF-cKO* and control mice (control, n=3; BDNF-cKO, n = 4). Scale bar = 100 μm. Data represent mean ± SEM. n.s., not significant. Unpaired two-tailed Student's t test for **(B, D)**. The statistical details can be found in Table S1, Supporting Information.

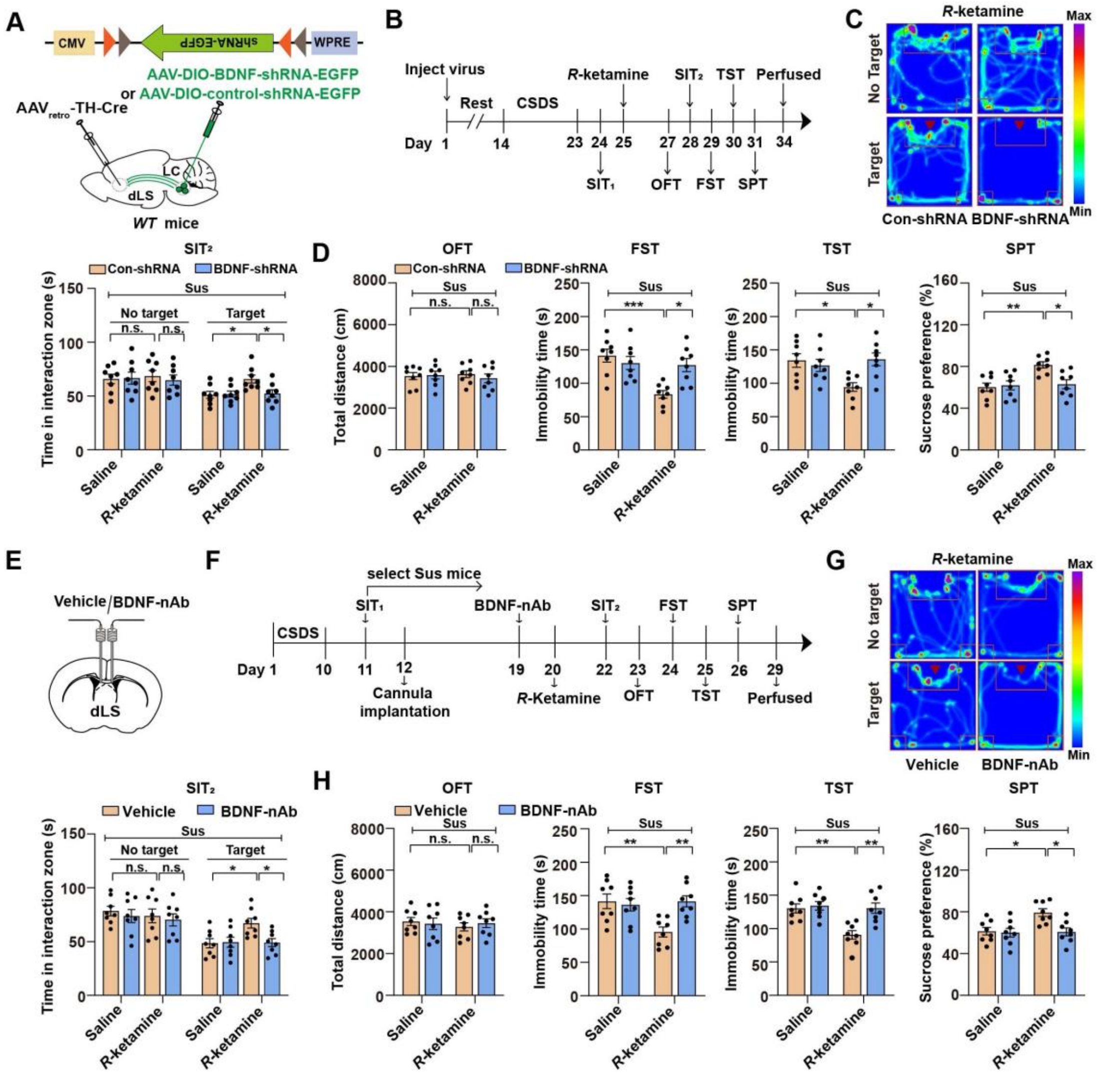

**Figure S23. BDNF was necessary in the $LC^{TH}$-dLS circuit or dLS to sustain the antidepressant effects of *R*-ketamine.** **(A)** Schematic representation of virus injection. **(B)** Experimental scheme. **(C)** Representative social interaction tracks of Con-shRNA and BDNF-shRNA mice and the time spent in the interaction zone in the SIT in different groups (n = 8). **(D)** Locomotor activity and depression-like behaviors in Con-shRNA and BDNF-shRNA mice (n = 8). **(E)** Schematic of injection strategy. **(F)** Timeline of experiments. **(G)** Representative social interaction tracks of mice infused with vehicle or BDNF-nAb and the time spent in the interaction zone in the

SIT in different groups (n = 8). BDNF-nAb, BDNF neutralizing antibody. **(H)** Locomotor activity and depression-like behaviors in susceptible mice infused with vehicle or BDNF-nAb (n = 8). Data represented as mean ± SEM. *P < 0.05, **P < 0.01, ***P < 0.001; n.s., not significant. Two-way ANOVA followed by Bonferroni's post hoc analysis for **(C)**, **(D)**, **(G)** and **(H)**. The statistical details can be found in Table S1, Supporting Information.

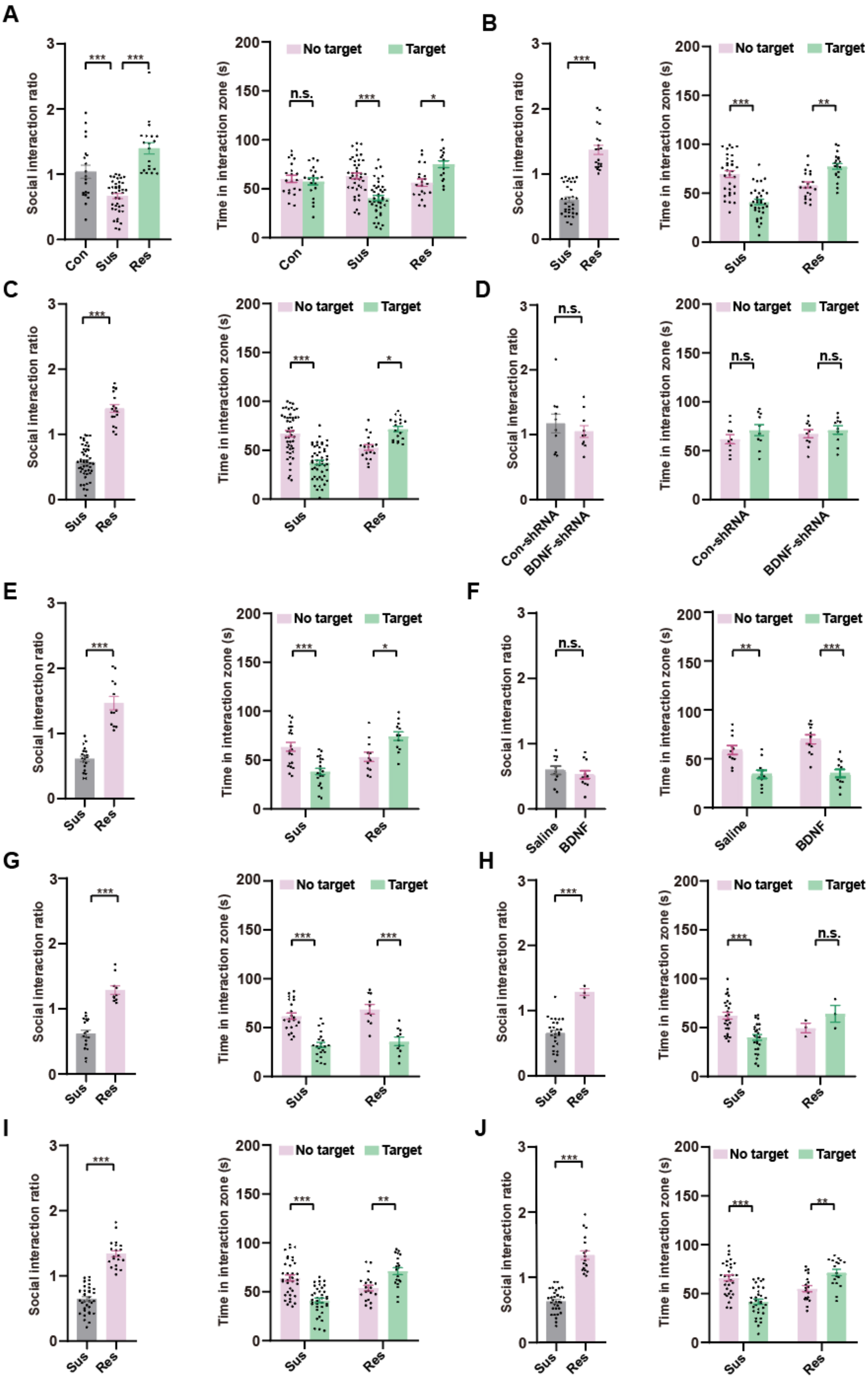


**Figure S24. Data of SIT$_1$ in Fig. 1H, 3H, 4G, 5J, 5N, 6B, 6K, 7B, 7F. (A)** Social

interaction ratio and the time spent in the interaction zone in the SIT in different groups in Fig. 1H (control, n = 20; susceptible, n = 40; resilient, n = 20). **(B)** Social interaction ratio and the time spent in the interaction zone in the SIT in different groups in Fig. 3H (susceptible, n = 32; resilient, n = 20). **(C)** Social interaction ratio and the time spent in the interaction zone in the SIT in different groups in Fig. 4G (susceptible, n = 48; resilient, n = 18). **(D)** Social interaction ratio and the time spent in the interaction zone in the SIT in different groups in Fig. 5J (con-shRNA, n = 10; BDNF-shRNA, n = 10;). **(E)** Social interaction ratio and the time spent in the interaction zone in the SIT in different groups in Fig. 5N (susceptible, n = 20; resilient, n = 12). **(F)** Social interaction ratio and the time spent in the interaction zone in the SIT in different groups in Fig. 6B (Saline, n = 11; BDNF, n = 11). **(G)** Social interaction ratio and the time spent in the interaction zone in the SIT in *TH-Cre* mice in Fig. 6K (susceptible, n = 18; resilient, n = 10). **(H)** Social interaction ratio and the time spent in the interaction zone in the SIT in *BDNF-cKO* mice in Fig. 6K (susceptible, n = 26; resilient, n = 3). **(I)** Social interaction ratio and the time spent in the interaction zone in the SIT in different groups in Fig. 7B (susceptible, n = 36; resilient, n = 20). **(J)** Social interaction ratio and the time spent in the interaction zone in the SIT in different groups in Fig. 7F (susceptible, n = 32; resilient, n = 18). Data represented as mean ± SEM. $^*P < 0.05$, $^{**}P < 0.01$, $^{***}P < 0.001$; n.s., not significant. The statistical details can be found in Table S1, Supporting Information.

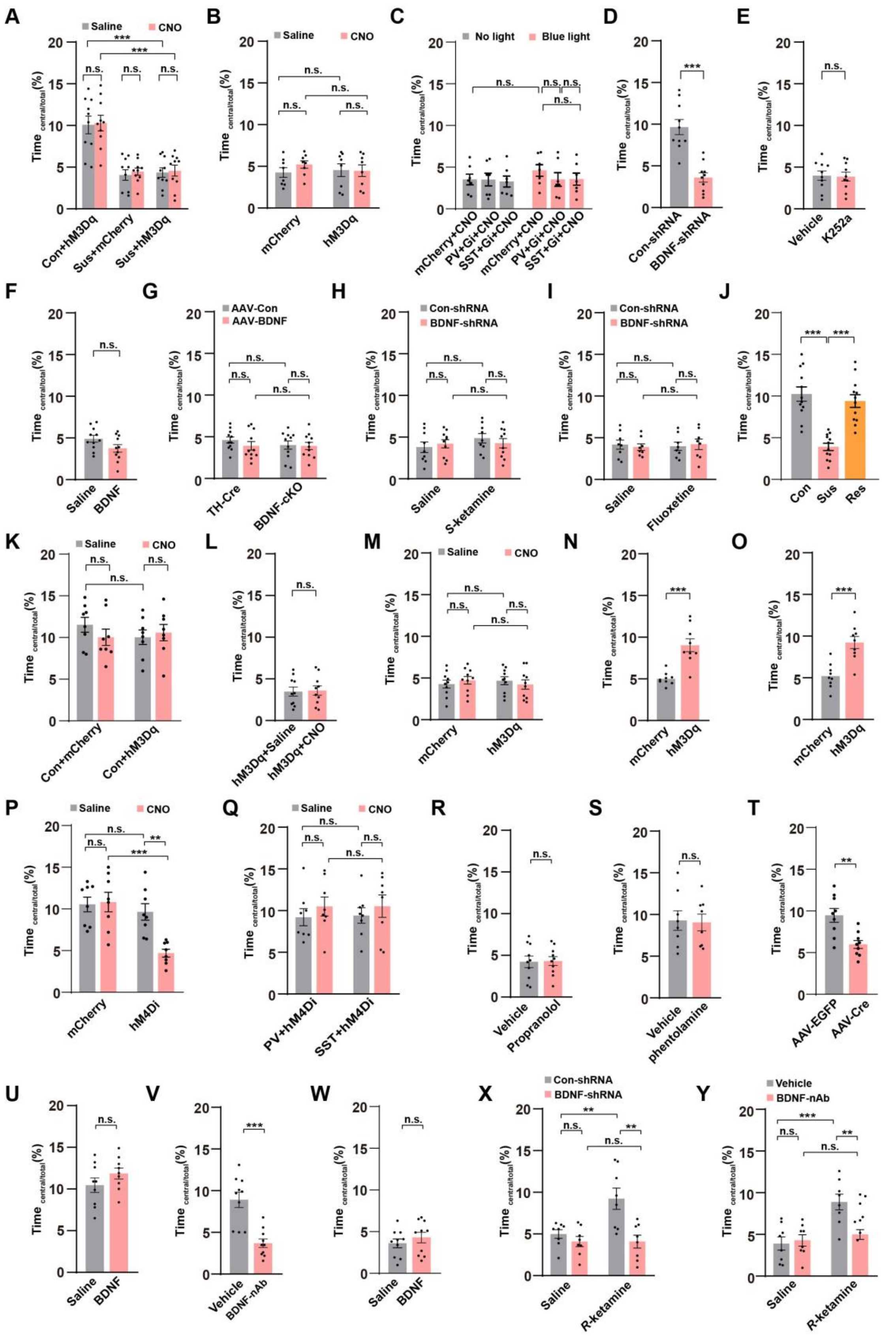


**Figure S25. Results of time spent in the center of open filed test in Fig. 1N, 3K, 4J,**

**5L, 5Q, 6F, 6O, 7D, 7H, S1C, S6D, S7D, S9D, S10D, S10H, S11E, S13E, S14F, S14J, S16G, S19D, S20D, S21D, S23D, S23H.** **(A)** Ratio of the time spent in the center of arena in OFT in different groups in Fig. 1N (n = 10). **(B)** Ratio of the time spent in the center of arena in OFT in different groups in Fig. 3K (n = 8). **(C)** Ratio of the time spent in the center of arena in OFT in different groups in Fig. 4J (n = 7). **(D)** Ratio of the time spent in the center of arena in OFT in different groups in Fig. 5L (n = 10). **(E)** Ratio of the time spent in the center of arena in OFT in different groups in Fig. 5Q (n = 10). **(F)** Ratio of the time spent in the center of arena in OFT in different groups in Fig. 6F (n = 10). **(G)** Ratio of the time spent in the center of arena in OFT in different groups in Fig. 6O (n = 11). **(H)** Ratio of the time spent in the center of arena in OFT in different groups in Fig. 7D (n = 9). **(I)** Ratio of the time spent in the center of arena in OFT in different groups in Fig. 7H (n = 8). **(J)** Ratio of the time spent in the center of arena in OFT in different groups in Fig. S1C (n = 12). **(K)** Ratio of the time spent in the center of arena in OFT in different groups in Fig. S6D (n = 8). **(L)** Ratio of the time spent in the center of arena in OFT in different groups in Fig. S7D (n = 10). **(M)** Ratio of the time spent in the center of arena in OFT in different groups in Fig. S9D (n = 10). **(N)** Ratio of the time spent in the center of arena in OFT in different groups in Fig. S10D (n = 9). **(O)** Ratio of the time spent in the center of arena in OFT in different groups in Fig. S10H (n = 9). **(P)** Ratio of the time spent in the center of arena in OFT in different groups in Fig. S11E (n = 8). **(Q)** Ratio of the time spent in the center of arena in OFT in different groups in Fig. S13E (n = 8). **(R)** Ratio of the time spent in the center of arena in OFT in different groups in Fig. S14F

(n = 10). **(S)** Ratio of the time spent in the center of arena in OFT in different groups in Fig. S14J (n = 8). **(T)** Ratio of the time spent in the center of arena in OFT in different groups in Fig. S16G (n = 9). **(U)** Ratio of the time spent in the center of arena in OFT in different groups in Fig. S19D (n = 9). **(V)** Ratio of the time spent in the center of arena in OFT in different groups in Fig. S20D (n = 10). **(W)** Ratio of the time spent in the center of arena in OFT in different groups in Fig. S21D (n = 10). **(X)** Ratio of the time spent in the center of arena in OFT in different groups in Fig. S23D (n = 8). **(Y)** Ratio of the time spent in the center of arena in OFT in different groups in Fig. S23H (n = 8). Data represented as mean ± SEM. *P < 0.05, **P < 0.01, ***P < 0.001; n.s., not significant. The statistical details can be found in Table S1, Supporting Information.

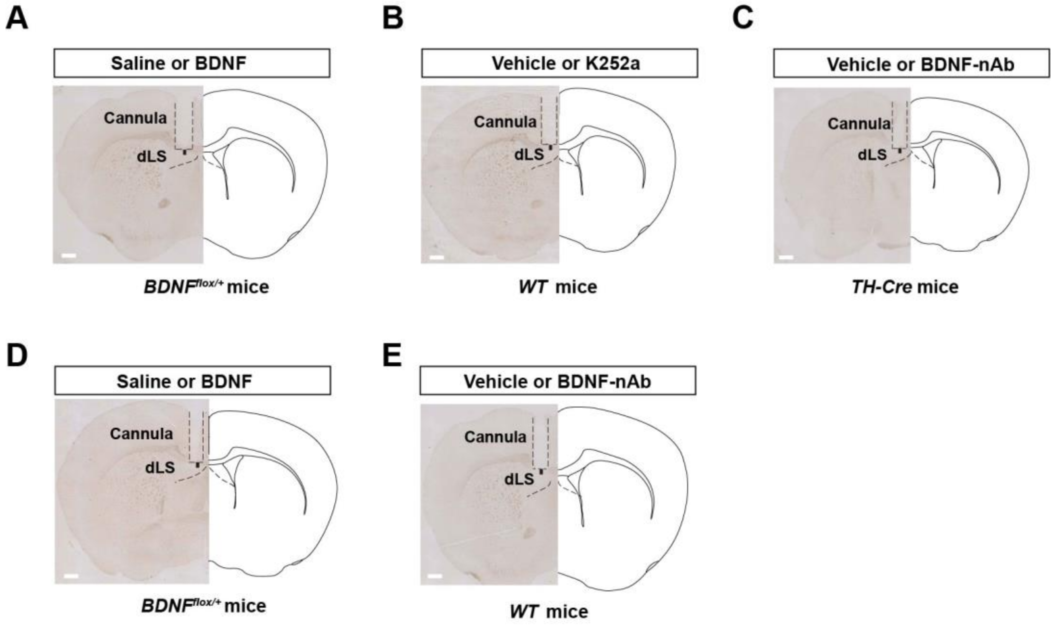


**Figure S26. Representative photos of infusion sites of BDNF, K252a, and anti-BDNF-nAb in Figs. 6, S19, S20, S21, and S23. (A)** The example slice showing

the location of cannula in Figure 6. Scale bar = 500 μm. **(B)** The example slice showing the location of cannula in Figure S19. Scale bar = 500 μm. **(C)** The example slice showing the location of cannula in Figure S20. Scale bar = 500 μm. **(D)** The example slice showing the location of cannula in Figure S21. Scale bar = 500 μm. **(E)** The example slice showing the location of cannula in Figure S23. Scale bar = 500 μm.

**Supplementary Table 1 Statistics.**
**Figure 1-7**

| Fig | Statistic methods | F/t value | P value | Post hoc multiple comparisons test |
|---|---|---|---|---|
| **1F** | One-way ANOVA, post hoc Tukey's test | F (2, 12) = 11.22 | P = 0.0018 | Con vs. Sus, P = 0.0057** Sus vs. Res, P = 0.0028** |
| **1J** | Unpaired two-tailed Student's t-test | _ | _ | Saline vs. CNO, P < 0.0001*** |
| **1K** | Two-way ANOVA, post hoc Bonferroni's test | Interaction: F (2, 54) = 0.4954 | P = 0.6121 | Target: Con+hM3Dq+Saline vs. Con+hM3Dq+CNO, P > 0.05 |
| | | Treatment: F (2, 54) = 47.62 | P < 0.0001 | Target: Sus + mCherry + Saline vs. Sus + mCherry + CNO, P > 0.05 |
| | | Groups: F (1, 54) = 0.02189 | P = 0.8829 | Target: Sus+hM3Dq+Saline vs. Sus+hM3Dq+CNO, P > 0.05 Target: Con+hM3Dq+Saline vs.sus+hM3Dq+saline, P < 0.0001*** Target: Sus + mCherry + CNO vs.Sus+hM3Dq+CNO, P < 0.0001*** Target: Sus + mCherry +Saline |

| | | | | |
|---|---|---|---|---|
| | | | | vs.Sus+hM3Dq+Saline, P > 0.05 |
| **1L** | Two-way ANOVA, post hoc Bonferroni's test | Interaction: F (2, 54) = 2.598 | P = 0.0837 | Target: Con+hM3Dq+Saline vs. Con+hM3Dq+CNO, P > 0.05 |
| | | Treatment: F (2, 54) = 56.03 | P < 0.0001 | Target: Sus + mCherry + Saline vs. Sus + mCherry + CNO, P > 0.05 |
| | | Groups: F (1, 54) = 0.4327 | P = 0.5135 | Target: Sus+hM3Dq+Saline vs. Sus+hM3Dq+CNO, P > 0.05 |
| | | | | Target: Con+hM3Dq+Saline vs.sus+hM3Dq+saline, P < 0.0001*** |
| | | | | Target: Sus + mCherry + CNO vs. Sus+hM3Dq+CNO, P = 0.0015** |
| | | | | Target: Sus + mCherry +Saline vs. Sus+hM3Dq+Saline, P > 0.05 |
| **1M** | Two-way ANOVA, post hoc Bonferroni's test | Interaction: F (2, 54) = 4.417 | P = 0.0167 | Target: Con+hM3Dq+Saline vs. Con+hM3Dq+CNO, P > 0.05 |
| | | Treatment: F (2, 54) = 34.84 | P < 0.0001 | Target: Sus+ mCherry + Saline vs. Sus + mCherry + CNO, P > 0.05 |
| | | Groups: F (1, 54) = 4.545 | P = 0.0376 | Target: Sus+hM3Dq+Saline vs. Sus+hM3Dq+CNO, P = 0.0088** |
| | | | | Target: Con+hM3Dq+Saline vs.sus+hM3Dq+saline, P < 0.0001*** |
| | | | | Target: Sus + mCherry + CNO vs. Sus+hM3Dq+CNO,P> 0.05 |

| | | | | |
|---|---|---|---|---|
| | | | | Target: Sus + mCherry +Saline vs. Sus + hM3Dq + Saline, P > 0.05 |
| **1N (OFT)** | Two-way ANOVA, post hoc Bonferroni's test | Interaction: F (2, 54) = 0.4872 | P = 0.6170 | Con+hM3Dq+Saline vs. Con+hM3Dq+CNO, P > 0.05 |
| | | Treatment: F (2, 54) = 0.6490 | P = 0.6490 | Sus + mCherry + Saline vs. Sus + mCherry + CNO, P > 0.05 |
| | | Groups: F (1, 54) = 0.5594 | P = 0.5594 | Sus+hM3Dq+Saline vs. Sus+hM3Dq+CNO, P > 0.05<br>Con+hM3Dq+Saline vs.sus+hM3Dq+saline, P > 0.05<br>Sus + mCherry + CNO vs.Sus+hM3Dq+CNO, P > 0.05 |
| **1N (FST)** | Two-way ANOVA, post hoc Bonferroni's test | Interaction: F (2, 54) = 5.548 | P = 0.0064 | Con+hM3Dq+Saline vs. Con+hM3Dq+CNO, P > 0.05 |
| | | Treatment: F (2, 54) = 21.22 | P < 0.0001 | Sus + mCherry + Saline vs. Sus + mCherry + CNO, P > 0.05 |
| | | Groups: F (1, 54) = 3.382 | P = 0.0714 | Sus+hM3Dq+Saline vs. Sus+hM3Dq+CNO, P = 0.0073**<br>Con+hM3Dq+Saline vs.sus+hM3Dq+saline, P < 0.0001***<br>Sus + mCherry + CNO vs. Sus +hM3Dq+CNO, P > 0.05 |
| **1N (TST)** | Two-way ANOVA, post hoc Bonferroni's test | Interaction: F (2, 54) = 22.21 | P < 0.0001 | Con+hM3Dq+Saline vs. Con+hM3Dq+CNO, P > 0.05 |
| | | Treatment: F (2, 54) = 47.58 | P < 0.0001 | Sus + mCherry + Saline vs. Sus+mCherry+CNO, P > 0.05 |
| | | Groups: F (1, 54) = 13.26 | P = 0.0006 | Sus+hM3Dq+Saline vs. Sus+hM3Dq+CNO, P < 0.0001***<br>Con+hM3Dq+Saline |

| | | | | |
|---|---|---|---|---|
| | | | | vs.sus+hM3Dq+saline, P < 0.0001*** |
| | | | | Sus + mCherry + CNO vs. Sus +hM3Dq+CNO, P > 0.05 |
| **1N (SPT)** | Two-way ANOVA, post hoc Bonferroni's test | Interaction: F (2, 54) = 8.121 | P = 0.0008 | Con+hM3Dq+Saline vs. Con+hM3Dq+CNO, P > 0.05 |
| | | Treatment: F (2, 54) = 35.91 | P < 0.0001 | Sus + mCherry + Saline vs. Sus + mCherry + CNO, P > 0.05 |
| | | Groups: F (1, 54) = 14.15 | P = 0.0004 | Sus + hM3Dq + Saline vs. Sus + hM3Dq + CNO, P < 0.0001*** |
| | | | | Con+hM3Dq+Saline vs.sus+hM3Dq+saline, P < 0.0001*** |
| | | | | Sus + mCherry + CNO vs.Sus+hM3Dq+CNO, P > 0.05 |
| **3B** | Unpaired two-tailed Student's t-test | _ | _ | dLS (con vs. sus), P < 0.0001*** |
| **3D** | Unpaired two-tailed Student's t-test | _ | _ | mPFCF (con vs. sus), P > 0.05 |
| **3F** | Unpaired two-tailed Student's t-test | _ | _ | CeA (con vs. sus), P > 0.05 |
| **3J** | Two-way ANOVA, post hoc Bonferroni's test | Interaction: F (1, 28) = 19.32 | P = 0.0001 | Target: mCherry + Saline vs. hM3Dq+Saline, P > 0.05 |
| | | Treatment: F (1, 28) = 34.52 | P < 0.0001 | Target: mCherry + CNO vs. hM3Dq+CNO, P < 0.0001*** |
| | | Groups: F (1, 28) = 40.63 | P < 0.0001 | Target: hM3Dq + Saline vs. hM3Dq + CNO, P < 0.0001*** |
| **3K (OFT)** | Two-way ANOVA, post hoc Bonferroni's test | Interaction: F (1, 28) = 0.02363 | P = 0.8789 | mCherry + Saline vs. hM3Dq + Saline, P > 0.05 |
| | | Treatment: F (1, 28) = | P = 0.3275 | mCherry + CNO vs. hM3Dq + CNO, P > 0.05 |

| | | | | |
|---|---|---|---|---|
| | | 0.9933 | | |
| | | Groups: F (1, 28) = 0.1151 | P = 0.7369 | hM3Dq + Saline vs. hM3Dq + CNO, P > 0.05 |
| **3K (FST)** | Two-way ANOVA, post hoc Bonferroni's test | Interaction: F (1, 28) = 2.023 | P = 0.1660 | mCherry + Saline vs. hM3Dq + Saline, P > 0.05 |
| | | Treatment: F (1, 28) = 8.539 | P = 0.0068 | mCherry + CNO vs. hM3Dq+CNO, P = 0.0282* |
| | | Groups: F (1, 28) = 11.48 | P = 0.0021 | hM3Dq+Saline vs. hM3Dq+CNO, P = 0.0122* |
| **3K (TST)** | Two-way ANOVA, post hoc Bonferroni's test | Interaction: F (1, 28) = 1.024 | P = 0.3202 | mCherry + Saline vs. hM3Dq+Saline, P > 0.05 |
| | | Treatment: F (1, 28) = 11.02 | P = 0.0025 | mCherry + CNO vs. hM3Dq+CNO, P = 0.0288* |
| | | Groups: F (1, 28) = 10.90 | P = 0.0026 | hM3Dq+Saline vs. hM3Dq+CNO, P = 0.0298* |
| **3K (SPT)** | Two-way ANOVA, post hoc Bonferroni's test | Interaction: F (1, 28) = 7.578 | P = 0.0103 | mCherry + Saline vs. hM3Dq + Saline, P > 0.05 |
| | | Treatment: F (1, 28) = 14.68 | P = 0.0007 | mCherry + CNO vs. hM3Dq+CNO, P = 0.0004*** |
| | | Groups: F (1, 28) = 4.339 | P = 0.0465 | hM3Dq+Saline vs. hM3Dq+CNO, P = 0.0117* |
| **4H** | Unpaired two-tailed Student's t-test | _ | _ | mCherry + CNO vs. Gi +CNO, P = 0.0026** |
| **4I** | Two-way ANOVA, post hoc Bonferroni's test | Interaction: F (2, 36) = 5.234 | P = 0.0101 | Target: No light: mCherry + CNO vs. Light: mCherry + CNO, P = 0.0108* |
| | | Treatment: F (1, 36) = 15.50 | P = 0.0004 | Target: Light: mCherry + CNO vs. Light: PV + GI， P > 0.05 |
| | | Groups: F(2, 36) = 3.119 | P = 0.0563 | Target: Light: mCherry + CNO vs. Light: SST + GI, P = 0.0112* |
| | | | | Target: Light: PV + GI vs. Light: SST + GI, P = 0.0337* |
| **4J (OFT)** | Two-way ANOVA, post hoc Bonferroni's test | Interaction: F (2, 36) = 0.2507 | P = 0.7796 | No light: mCherry + CNO vs. Light: mCherry + CNO, P > 0.05 |
| | | Treatment: F (1, 36) = 0.07470 | P = 0.7862 | Light: mCherry + CNO vs. Light: PV + GI， P > 0.05 |

| | | | | |
|---|---|---|---|---|
| | | Groups: F (2, 36) = 0.1988 | P = 0.8206 | Light: mCherry + CNO vs. Light: SST+GI, P > 0.05<br>Light: PV+GI vs. Light: SST+GI, P > 0.05 |
| **4J (FST)** | Two-way ANOVA, post hoc Bonferroni's test | Interaction: F (2, 35) = 4.345 | P = 0.0206 | No light: mCherry + CNO vs. Light: mCherry + CNO, P = 0.0039** |
| | | Treatment: F (1, 35) = 21.86 | P < 0.0001 | Light: mCherry + CNO vs. Light: PV + GI， P > 0.9999 |
| | | Groups: F(2, 35) = 5.605 | P = 0.0077 | Light: mCherry + CNO vs. Light: SST+GI, P = 0.0052**<br>Light: PV+GI vs. Light: SST+GI, P = 0.0133* |
| **4J (TST)** | Two-way ANOVA, post hoc Bonferroni's test | Interaction: F (2, 36) = 7.542 | P = 0.0018 | No light: mCherry + CNO vs. Light: mCherry + CNO, P = 0.0002*** |
| | | Treatment: F (1, 36) = 27.06 | P < 0.0001 | Light: mCherry + CNO vs. Light: PV+GI， P > 0.9999 |
| | | Groups: F (2, 36) = 2.010 | P = 0.1488 | Light: mCherry + CNO vs. Light: SST+GI, P = 0.0076**<br>Light: PV+GI vs. Light: SST+GI, P = 0.0331* |
| **4J (SPT)** | Two-way ANOVA, post hoc Bonferroni's test | Interaction: F(2, 54) = 3.578 | P = 0.0347 | No light: mCherry + CNO vs. Light: mCherry + CNO, P = 0.0194* |
| | | Treatment: F(1, 54) = 22.33 | P < 0.0001 | Light: mCherry + CNO vs. Light: PV + GI， P > 0.9999 |
| | | Groups: F(2. 54) = 3.575 | P = 0.0348 | Light: mCherry + CNO vs. Light: SST+GI, P = 0.0424*<br>Light: PV + GI vs. Light: SST + GI, P = 0.0172* |
| **5C** | Unpaired two-tailed Student's t-test | _ | _ | *BDNF*: Con vs. Sus, P = 0.0455*<br>*Shank1*: Con vs. Sus, P = 0.0439*<br>*Shank3*: Con vs. Sus, P = 0.0394* |

| | | | | |
|---|---|---|---|---|
| | | | | *Pllp*: Con vs. Sus, P = 0.0157* |
| **5D** | One-way ANOVA, post hoc Tukey's test | F (3, 8) = 15.56 | P = 0.0011 | Protein-LC: Con vs. Sus, P = 0.0025** |
| | | | | Protein-dLS: Con vs. Sus, P = 0.0200* |
| **5H** | Unpaired two-tailed Student's t-test | _ | _ | BDNF（Con vs. ChR2), P = 0.0288* |
| **5K** | Two-way ANOVA, post hoc Bonferroni's test | Interaction: F (1, 36) = 7.954 | P = 0.0078 | Target: Pre SSDS (Con -shRNA vs. BDNF-shRNA), P > 0.05 |
| | | Treatment: F (1, 36) = 3.658 | P = 0.0638 | Target: Post SSDS (Con -shRNA vs. BDNF-shRNA), P = 0.0115* |
| | | Groups: F (1, 36) = 5.181 | P = 0.0289 | Target: BDNF-shRNA (Pre SSDS vs. Post SSDS), P = 0.0056** |
| **5L (OFT)** | Unpaired two-tailed Student's t-test | _ | _ | AAV-EGFP vs. AAV-Cre, P > 0.05 |
| **5L (FST)** | Unpaired two-tailed Student's t-test | _ | _ | AAV-EGFP vs. AAV-Cre, P = 0.0015** |
| **5L (TST)** | Unpaired two-tailed Student's t-test | _ | _ | AAV-EGFP vs. AAV-Cre, P = 0.0054** |
| **5L (SPT)** | Unpaired two-tailed Student's t-test | _ | _ | AAV-EGFP vs. AAV-Cre, P = 0.0017** |
| **5P** | Two-way ANOVA, post hoc Bonferroni's test | Interaction: F (1, 36) = 3.186 | P = 0.0001 | Target: Vehicle vs. K252a, P = 0.0056** |
| | | Treatment: F (1, 36) = 4.225 | P = 0.0471 | Vehicle (no target vs target), P > 0.05 |
| | | Groups: F (1, 36) = 11.02 | P = 0.0021 | No target (Vehicle vs K252a), P > 0.05 |
| **5Q (OFT)** | Unpaired two-tailed Student's t-test | _ | _ | hM3Dq (CNO): Vehicle vs. K252a P > 0.05 |
| **5Q (FST)** | Unpaired two-tailed | _ | _ | hM3Dq (CNO): Vehicle vs. K252A, P = 0.0096** |

| | | | | |
|---|---|---|---|---|
| | Student's t-test | | | |
| **5Q (TST)** | Unpaired two-tailed Student's t-test | _ | _ | hM3Dq (CNO): Vehicle vs. K252A, P = 0.0015** |
| **5Q (SPT)** | Unpaired two-tailed Student's t-test | _ | _ | hM3Dq (CNO): Vehicle vs. K252A, P < 0.0001*** |
| **6D** | Unpaired two-tailed Student's t-test | _ | _ | Saline vs. BDNF, P = 0.048* |
| **6E** | Two-way ANOVA, post hoc Bonferroni's test | Interaction: F (1, 40) = 17.65 | P = 0.0001 | No target: SSDS + Saline vs. No target: SSDS+BDNF, P > 0.05 |
| | | Treatment: F (1, 40) = 17.12 | P = 0.0002 | No target: SSDS + Saline vs. Target: SSDS + Saline, P = 0.0003*** |
| | | Groups: F (1, 40) = 5.010 | P = 0.0308 | Target: SSDS + Saline vs. Target: SSDS + BDNF, P < 0.0001*** |
| **6F (OFT)** | Unpaired two-tailed Student's t-test | _ | _ | SSDS + Saline vs. SSDS + BDNF, P > 0.05 |
| **6F (FST)** | Unpaired two-tailed Student's t-test | _ | _ | SSDS + Saline vs. SSDS + BDNF, P = 0.0073** |
| **6F (TST)** | Unpaired two-tailed Student's t-test | _ | _ | SSDS + Saline vs. SSDS + BDNF, P =0.0201* |
| **6F (SPT)** | Unpaired two-tailed Student's t-test | _ | _ | SSDS + Saline vs. SSDS + BDNF, P = 0.0005*** |
| **6I** | One-way ANOVA, post hoc Tukey's test | F (3, 12) = 28.33, | P<0.0001 | LC: Con vs. Sus, P < 0.0001***<br>dLS: Con vs. Sus, P = 0.0005*** |
| **6M** | One-way ANOVA, post hoc Tukey's test | F (3, 8) = 5.995, | P = 0.0192 | *TH-Cre* mice: AAV-Con vs. AAV-BDNF P = 0.0465*<br>BDNF-cKO mice: AAV-Con vs. AAV-BDNF P = 0.0288* |
| **6N** | Two-way ANOVA, post hoc | Interaction: F (1, 40) = 0.1132 | P = 0.7382 | Target: Con + AAV-Con vs. Con + AAV-BDNF, P = |

| | | | | |
|---|---|---|---|---|
| | Bonferroni's test | | | 0.0002*** |
| | | Treatment: F (1, 40) = 15.23 | P = 0.0004 | Target: Con + AAV-Con vs. cKO + AAV-Con, P = 0.0280* |
| | | Groups: F (1, 40) = 48.05 | P < 0.0001 | Target: cKO + AAV-Con vs. cKO + AAV-BDNF, P < 0.0001*** |
| **6O (OFT)** | Two-way ANOVA, post hoc Bonferroni's test | Interaction: F (1, 40) = 0.04480 | P = 0.8335 | Con + AAV-Con vs. Con + AAV-BDNF, P > 0.05 |
| | | Treatment: F (1, 40) = 1.026 | P = 0.3172 | Con + AAV-Con vs. cKO + AAV-Con, P > 0.05 |
| | | Groups: F (1, 40) = 0.05761 | P = 0.8115 | cKO + AAV-Con vs. cKO + AAV-BDNF, P > 0.05 |
| **6O (FST)** | Two-way ANOVA, post hoc Bonferroni's test | Interaction: F (1, 40) = 7.738 | P = 0.0082 | Con + AAV-Con vs. Con + AAV-BDNF, P = 0.0361* |
| | | Treatment: F (1, 40) = 2.564 | P = 0.1172 | Con + AAV-Con vs. cKO + AAV-Con, P = 0.0213* |
| | | Groups: F (1, 40) = 47.39 | P < 0.0001 | cKO + AAV-Con vs. cKO + AAV-BDNF, P < 0.0001*** |
| **6O (TST)** | Two-way ANOVA, post hoc Bonferroni's test | Interaction: F (1, 40) = 3.791 | P = 0.0586 | Con + AAV-Con vs. Con + AAV-BDNF, P = 0.0030** |
| | | Treatment: F (1, 40) = 2.807 | P = 0.1017 | Con + AAV-Con vs. cKO + AAV-Con, P = 0.0858 |
| | | Groups: F (1, 40) = 53.36 | P < 0.0001 | cKO + AAV-Con vs. cKO + AAV-BDNF, P < 0.0001*** |
| **6O (SPT)** | Two-way ANOVA, post hoc Bonferroni's test | Interaction: F (1, 40) = 5.501 | P = 0.0241 | Con + AAV-Con vs. Con + AAV-BDNF, P = 0.0003*** |
| | | Treatment: F (1, 40) = 4.020 | P = 0.0518 | Con + AAV-Con vs. cKO + AAV-Con, P = 0.0226* |
| | | Groups: F (1, 40) = 76.96 | P < 0.0001 | cKO + AAV-Con vs. cKO +AAV-BDNF, P < 0.0001*** |
| **6P** | One-way ANOVA, Fisher's exact test | _ | _ | Correlation: P = 0.0295* |
| **7C** | Two-way ANOVA, post hoc Bonferroni's test | Interaction: F (1, 32) = 5.445 | P = 0.0261 | Target: Con-shRNA (Saline vs. ketamine), P = 0.0035** |
| | | Treatment: F | P = 0.0018 | Target: Ketamine |

| | | | | |
|---|---|---|---|---|
| | | (1, 32) = 11.59 | | (Con-shRNA vs. BDNF-shRNA), P = 0.0018** |
| | | Groups: F (1, 32) = 9.364 | P = 0.0045 | |
| **7D (OFT)** | Two-way ANOVA, post hoc Bonferroni's test | Interaction: F (1, 32) = 0.3266 | P = 0.5717 | Con-shRNA (Saline vs. Ketamine), P > 0.05 |
| | | Treatment: F (1, 32) = 0.4545 | P = 0.5050 | Ketamine (Con-shRNA vs. BDNF-shRNA), P > 0.05 |
| | | Groups: F (1, 32) = 0.01366 | P = 0.9077 | |
| **7D (FST)** | Two-way ANOVA, post hoc Bonferroni's test | Interaction: F (1, 32) = 0.6395 | P = 0.4298 | Con-shRNA (Saline vs. Ketamine), P = 0.0307* |
| | | Treatment: F (1, 32) = 11.91 | P = 0.0016 | Ketamine (Con-shRNA vs. BDNF-shRNA), P = 0.0421* |
| | | Groups: F (1, 32) = 10.73 | P = 0.0025 | |
| **7D (TST)** | Two-way ANOVA, post hoc Bonferroni's test | Interaction: F (1, 32) = 4.150 | P = 0.0500 | Con-shRNA (Saline vs. Ketamine), P = 0.0022** |
| | | Treatment: F (1, 32) = 12.97 | P = 0.0011 | Ketamine (Con-shRNA vs. BDNF-shRNA), P > 0.05 |
| | | Groups: F (1, 32) = 3.693 | P = 0.0636 | |
| **7D (SPT)** | Two-way ANOVA, post hoc Bonferroni's test | Interaction: F (1, 32) = 3.460 | P = 0.0721 | Con-shRNA (Saline vs. Ketamine), P = 0.0124* |
| | | Treatment: F (1, 32) = 8.311 | P = 0.0070 | Ketamine (Con-shRNA vs. BDNF-shRNA), P = 0.0022** |
| | | Groups: F (1, 32) = 14.17 | P = 0.0007 | |
| **7G** | Two-way ANOVA, post hoc Bonferroni's test | Interaction: F (1, 28) = 0.2076 | P = 0.6521 | Target: Con-shRNA (Saline vs. Fluoxetine), P = 0.0003*** |
| | | Treatment: F (1, 28) = 0.1511 | P = 0.7004 | Target: Fluoxetine (Con-shRNA vs. BDNF-shRNA), P > 0.05 |
| | | Groups: F (1, 28) = 40.48 | P < 0.0001 | |
| **7H (OFT)** | Two-way | Interaction: F | P = 0.7513 | Con-shRNA (Saline vs. |

| | | | | |
|---|---|---|---|---|
| | ANOVA, post hoc Bonferroni's test | (1, 28) = 0.1024 | | Fluoxetine), P > 0.05 |
| | | Treatment: F (1, 28) = 0.6023 | P = 0.4442 | Fluoxetine (Con-shRNA vs. BDNF-shRNA), P > 0.05 |
| | | Groups: F (1, 28) = 0.3767 | P = 0.5443 | |
| **7H (FST)** | Two-way ANOVA, post hoc Bonferroni's test | Interaction: F (1, 28) = 0.3932 | P = 0.5357 | Con-shRNA (Saline vs. Fluoxetine), P = 0.0051** |
| | | Treatment: F (1, 28) = 34.92 | P < 0.0001 | Fluoxetine (Con-shRNA vs. BDNF-shRNA), P > 0.05 |
| | | Groups: F (1, 28) = 0.09149 | P = 0.7645 | |
| **7H (TST)** | Two-way ANOVA, post hoc Bonferroni's test | Interaction: F (1, 28) = 0.3525 | P = 0.5575 | Con-shRNA (Saline vs. Fluoxetine), P = 0.0111* |
| | | Treatment: F (1, 28) = 29.76 | P < 0.0001 | Fluoxetine (Con-shRNA vs. BDNF-shRNA), P > 0.05 |
| | | Groups: F (1, 28) = 0.9158 | P = 0.3468 | |
| **7H (SPT)** | Two-way ANOVA, post hoc Bonferroni's test | Interaction: F (1, 28) = 0.0007843 | P = 0.9779 | Con-shRNA (Saline vs. Fluoxetine), P = 0.0185* |
| | | Treatment: F (1, 28) = 20.74 | P < 0.0001 | Fluoxetine (Con-shRNA vs. BDNF-shRNA), P > 0.05 |
| | | Groups: F (1, 28) = 1.344 | P = 0.2561 | |

**Supplementary Figure 1-25**

| **Fig** | **Statistic methods** | **F/t value** | **P value** | **Post hoc multiple comparisons test** |
|---|---|---|---|---|
| **1A** | One-way ANOVA, post hoc Tukey's test | F (2, 33) = 21.88 | P < 0.0001 | Con vs. Sus, P < 0.0001*** |
| | | | | Sus vs. Res, P < 0.0001*** |
| **1B** | Two-way ANOVA, post hoc Bonferroni's test | Interaction: F (2, 66) = 9.904 | P = 0.0002 | Con (No target vs. Target), P > 0.05 |
| | | Treatment: F (2, 66) = 12.43 | P < 0.0001 | Sus (No target vs. Target), P = 0.0006*** |
| | | Groups: F (1, 66) = 1.787 | P = 0.1859 | Res (No target vs. Target), P > 0.05 |

| | | | | |
|---|---|---|---|---|
| **1C** | One-way ANOVA, post hoc Tukey's test | F (2, 33) = 0.1664 | P = 0.8474 | Con vs. Sus, P > 0.05 |
| | | | | Sus vs. Res, P > 0.05 |
| **1D** | One-way ANOVA, post hoc Tukey's test | F (2, 33) = 8.732 | P = 0.0009 | Con vs. Sus, P = 0.0023** |
| | | | | Sus vs. Res, P = 0.0037** |
| **1E** | One-way ANOVA, post hoc Tukey's test | F (2, 33) = 6.580 | P = 0.0039 | Con vs. Sus, P = 0.0143* |
| | | | | Sus vs. Res, P = 0.0068** |
| **1F** | One-way ANOVA, post hoc Tukey's test | F (2, 33) = 20.30 | P < 0.0001 | Con vs. Sus, P < 0.0001*** |
| | | | | Sus vs. Res, P = 0.0002*** |
| **1I** | Pearson correlation | _ | _ | r =0.8231, p < 0.0001*** |
| **1J** | One-way ANOVA, post hoc Tukey's test | F (2, 33) = 54.28 | P < 0.0001 | Con vs. Sus, P < 0.0001*** |
| | | | | Sus vs. Res, P < 0.0001*** |
| **3C** | One-way ANOVA, post hoc Tukey's test | F (2, 12) = 2.522 | P = 0.1218 | Con vs. Sus, P > 0.05 |
| | | | | Sus vs. Res, P > 0.05 |
| **4B** | Two-way ANOVA, post hoc Bonferroni's test | Interaction: F (9, 40) = 37.60 | P < 0.0001 | Day1: Pre-attack vs. Post-attack, P < 0.0001*** |
| | | Treatment: F (9, 40) = 45.86 | P < 0.0001 | Day2: Pre-attack vs. Post-attack, P < 0.0001*** |
| | | Groups: F (1, 40) = 893.8 | P < 0.0001 | Day3: Pre-attack vs. Post-attack, P < 0.0001*** |
| | | | | Day4: Pre-attack vs. Post-attack, P < 0.0001*** |
| | | | | Day5: Pre-attack vs. Post-attack, P < 0.0001*** |
| | | | | Day6: Pre-attack vs. Post-attack, P < 0.0001*** |

| | | | | |
|---|---|---|---|---|
| | | | | Day7: Pre-attack vs. Post-attack, P < 0.0001***<br>Day8: Pre-attack vs. Post-attack, P = 0.0052**<br>Day9: Pre-attack vs. Post-attack, P > 0.9999<br>Day10: Pre-attack vs. Post-attack, P > 0.9999 |
| **5D** | One-way ANOVA, post hoc Tukey's test | F (2, 12) = 19.07 | P = 0.0002 | Control vs. Susceptible, P < 0.0001***<br><br>Susceptible vs. Resilient, P = 0.0144* |
| **5H** | One-way ANOVA, post hoc Tukey's test | F (2, 12) = 23.34 | P < 0.0001 | Control vs. Susceptible, P = 0.0002***<br><br>Susceptible vs. Resilient, P = 0.0011** |
| **6C** | Two-way ANOVA, post hoc Bonferroni's test | Interaction: F (1, 28) = 1.715 | P = 0.2010 | Target: Con-mCherry: Saline vs. Con-mCherry: CNO, P > 0.9999 |
| | | Treatment: F (1, 28) = 1.715 | P = 0.2010 | Target: Con-mCherry: Saline vs. Con-Gq: Saline, P > 0.9999 |
| | | Groups: F (1, 28) = 1.715 | P = 0.2010 | Target: Con-Gq: Saline vs. Con-Gq: CNO, P > 0.9999<br>Target: Con-mCherry: CNO vs. Con-Gq: CNO, P > 0.9999 |
| **6D** | Two-way ANOVA, post hoc Bonferroni's test | Interaction: F (1, 28) = 0.1610 | P = 0.6913 | Target: Con-mCherry: Saline vs. Con-mCherry: CNO, P > 0.9999 |
| | | Treatment: F (1, 28) = 0.2705 | P = 0.6071 | Target: Con-mCherry: Saline vs. Con-Gq: Saline, P > 0.9999 |
| | | Groups: F (1, 28) = 0.03962 | P = 0.8437 | Target: Con-Gq: Saline vs. Con-Gq: CNO, P > 0.9999<br>Target: Con-mCherry: CNO vs. Con-Gq: CNO, P > 0.9999 |
| **6E** | Two-way ANOVA, post hoc Bonferroni's test | Interaction: F (1, 28) = 1.312 | P = 0.2617 | Target: Con-mCherry: Saline vs. Con-mCherry: CNO, P > 0.9999 |

| | | | | |
|---|---|---|---|---|
| | | Treatment: F (1, 28) = 0.0005004 | P = 0.9823 | Target: Con-mCherry: Saline vs. Con-Gq: Saline, P > 0.9999 |
| | | Groups: F (1, 28) = 0.7700 | P = 0.3877 | Target: Con-Gq: Saline vs. Con-Gq: CNO, P > 0.9999<br>Target: Con-mCherry: CNO vs. Con-Gq: CNO, P > 0.9999 |
| **6F (OFT)** | Two-way ANOVA, post hoc Bonferroni's test | Interaction: F (1, 28) = 0.6529 | P = 0.4259 | Con-mCherry: Saline vs. Con-mCherry: CNO, P > 0.9999 |
| | | Treatment: F (1, 28) = 0.02878 | P = 0.8665 | Con-mCherry: Saline vs. Con-Gq: Saline, P > 0.9999 |
| | | Groups: F (1, 28) = 2.085 | P = 0.1599 | Con-Gq: Saline vs. Con-Gq: CNO, P > 0.9999<br>Con-mCherry: CNO vs. Con-Gq: CNO, P > 0.9999 |
| **6F (FST)** | Two-way ANOVA, post hoc Bonferroni's test | Interaction: F (1, 28) = 0.03506 | P = 0.8528 | Con-mCherry: Saline vs. Con-mCherry: CNO, P > 0.9999 |
| | | Treatment: F (1, 28) = 0.6061 | P = 0.4428 | Con-mCherry: Saline vs. Con-Gq: Saline, P > 0.9999 |
| | | Groups: F (1, 28) = 1.375 | P = 0.2508 | Con-Gq: Saline vs. Con-Gq: CNO, P > 0.9999<br>Con-mCherry: CNO vs. Con-Gq: CNO, P > 0.9999 |
| **6F (TST)** | Two-way ANOVA, post hoc Bonferroni's test | Interaction: F (1, 28) = 0.02636 | P = 0.8722 | Con-mCherry: Saline vs. Con-mCherry: CNO, P > 0.9999 |
| | | Treatment: F (1, 28) = 1.201 | P = 0.2825 | Con-mCherry: Saline vs. Con-Gq: Saline, P > 0.9999 |
| | | Groups: F (1, 28) = 0.003707 | P = 0.9519 | Con-Gq: Saline vs. Con-Gq: CNO, P > 0.9999<br>Con-mCherry: Saline vs. Con-mCherry: CNO, P > 0.9999 |
| **6F (SPT)** | Two-way ANOVA, post hoc Bonferroni's test | Interaction: F (1, 28) = 0.1656 | P = 0.6871 | Con-mCherry: Saline vs. Con-mCherry: CNO, P > 0.9999 |
| | | Treatment: F (1, 28) = | P = 0.9461 | Con-mCherry: Saline vs. Con-Gq: Saline, P > 0.9999 |

| | | | | |
|---|---|---|---|---|
| | | 0.004645 | | |
| | | Groups: F (1, 28) = 0.2059 | P = 0.6535 | Con-Gq: Saline vs. Con-Gq: CNO, P > 0.9999<br>Con-mCherry: Saline vs. Con-mCherry: CNO, P > 0.9999 |
| **7C** | Two-way ANOVA, post hoc Bonferroni's test | Interaction: F (1, 36) = 0.03179 | P = 0.8595 | hM3Dq-Saline (No target vs. Target), P = 0.0093** |
| | | Treatment: F (1, 36) = 0.1178 | P = 0.7334 | hM3Dq-CNO (No target vs. Target), P = 0.0046** |
| | | Groups: F (1, 36) = 25.20 | P < 0.0001 | Target (hM3Dq-Saline vs. hM3Dq-CNO), P > 0.05 |
| **7D** | Unpaired two-tailed Student's t-test | _ | _ | hM3Dq-Saline vs. hM3Dq-CNO, P > 0.05 |
| **7E** | Unpaired two-tailed Student's t-test | _ | _ | hM3Dq-Saline vs. hM3Dq-CNO, P > 0.05 |
| **7F** | Unpaired two-tailed Student's t-test | _ | _ | hM3Dq-Saline vs. hM3Dq-CNO, P > 0.05 |
| **7G** | Unpaired two-tailed Student's t-test | _ | _ | hM3Dq-Saline vs. hM3Dq-CNO, P > 0.05 |
| **8B** | One-way ANOVA, post hoc Tukey's test | F (2, 8) = 3.102 | P = 0.1006 | mPFC (Con vs. Sus), P > 0.05<br>mPFC (Sus vs. Res), P > 0.05 |
| | One-way ANOVA, post hoc Tukey's test | F (2, 8) = 10.66 | P = 0.0055 | dLS (Con vs. Sus), P = 0.0078**<br>dLS (Con vs. Res), P = 0.0096** |
| | One-way ANOVA, post hoc Tukey's test | F (2, 8) = 3.942 | P = 0.0644 | CeA (Con vs. Sus), P > 0.05<br>CeA (Sus vs. Res), P > 0.05 |
| **9C** | Two-way ANOVA, post hoc Bonferroni's test | Interaction: F (1, 36) = 22.07 | P < 0.0001 | Target (mCherry: Saline vs. hM3Dq: Saline), P > 0.05 |
| | | Treatment: F | P = 0.0333 | Target (mCherry:CNO vs. |

| | | | | |
|---|---|---|---|---|
| | | (1, 36) = 4.899 | | hM3Dq:CNO), P = 0.0001*** |
| | | Groups: F (1, 36) = 9.935 | P = 0.0033 | Target (hM3Dq: Saline vs. hM3Dq: CNO), P < 0.0001*** |
| **9D (OFT)** | Two-way ANOVA, post hoc Bonferroni's test | Interaction: F (1, 36) = 1.782e-005 | P = 0.9967 | mCherry: Saline vs. hM3Dq: Saline, P > 0.05 |
| | | Treatment: F (1, 36) = 1.962 | P = 0.1698 | mCherry: CNO vs. hM3Dq: CNO, P > 0.05 |
| | | Groups: F (1, 36) = 0.01511 | P = 0.9028 | hM3Dq: Saline vs. hM3Dq: CNO, P > 0.05 |
| **9D (FST)** | Two-way ANOVA, post hoc Bonferroni's test | Interaction: F (1, 36) = 5.268 | P = 0.0276 | mCherry: Saline vs. hM3Dq: Saline, P > 0.05 |
| | | Treatment: F (1, 36) = 2.405 | P = 0.1297 | mCherry: CNO vs. hM3Dq: CNO, P > 0.05 |
| | | Groups: F (1, 36) = 6.417 | P = 0.0158 | hM3Dq: Saline vs. hM3Dq: CNO, P = 0.0096** |
| **9D (TST)** | Two-way ANOVA, post hoc Bonferroni's test | Interaction: F (1, 36) = 2.127 | P = 0.1534 | mCherry: Saline vs. hM3Dq: Saline, P > 0.05 |
| | | Treatment: F (1, 36) = 5.672 | P = 0.0226 | mCherry: CNO vs. hM3Dq: CNO, P > 0.05 |
| | | Groups: F (1, 36) = 7.427 | P = 0.0099 | hM3Dq: Saline vs. hM3Dq: CNO, P = 0.0326* |
| **9D (SPT)** | Two-way ANOVA, post hoc Bonferroni's test | Interaction: F (1, 36) = 4.212 | P = 0.0475 | mCherry: Saline vs. hM3Dq: Saline, P > 0.05 |
| | | Treatment: F (1, 36) = 4.875 | P = 0.0337 | mCherry: CNO vs. hM3Dq: CNO, P = 0.0283* |
| | | Groups: F (1, 36) = 5.889 | P = 0.0204 | hM3Dq: Saline vs. hM3Dq: CNO, P = 0.0188* |
| **10C** | Two-way ANOVA, post hoc Bonferroni's test | Interaction: F (1, 32) = 1.360 | P = 0.2521 | mCherry (No target vs. Target), P = 0.043* |
| | | Treatment: F (1, 32) = 0.7530 | P = 0.3920 | Target (mCherry vs. hM3Dq), P > 0.05 |
| | | Groups: F (1, 32) = 8.385 | P = 0.0068 | |
| **10D (OFT)** | Unpaired | _ | _ | mCherry vs. hM3Dq, P > |

| | | | | |
|---|---|---|---|---|
| | two-tailed Student’s t-test | | | 0.05 |
| **10D (FST)** | Unpaired two-tailed Student’s t-test | _ | _ | mCherry vs. hM3Dq, P > 0.05 |
| **10D (TST)** | Unpaired two-tailed Student’s t-test | _ | _ | mCherry vs. hM3Dq, P > 0.05 |
| **10D (SPT)** | Unpaired two-tailed Student’s t-test | _ | _ | mCherry vs. hM3Dq, P > 0.05 |
| **10G** | Two-way ANOVA, post hoc Bonferroni’s test | Interaction: F (1, 32) = 1.925 | P = 0.1748 | mCherry (No target vs. Target), P = 0.0013** |
| | | Treatment: F (1, 32) = 1.763 | P = 0.1937 | Target (mCherry vs. hM3Dq), P > 0.05 |
| | | Groups: F (1, 32) = 20.19 | P < 0.0001 | |
| **10H (OFT)** | Unpaired two-tailed Student’s t-test | _ | _ | mCherry vs. hM3Dq, P > 0.05 |
| **10H (FST)** | Unpaired two-tailed Student’s t-test | _ | _ | mCherry vs. hM3Dq, P > 0.05 |
| **10H (TST)** | Unpaired two-tailed Student’s t-test | _ | _ | mCherry vs. hM3Dq, P > 0.05 |
| **10H (SPT)** | Unpaired two-tailed Student’s t-test | _ | _ | mCherry vs. hM3Dq, P > 0.05 |
| **11D** | Two-way ANOVA, post hoc Bonferroni’s test | Interaction: F (1, 28) = 14.46 | P = 0.0007 | Target: mCherry + Saline vs. hM4Di+Saline, P > 0.05 |
| | | Treatment: F (1, 28) = 10.69 | P = 0.0029 | Target: mCherry + CNO vs. hM4Di+CNO, P = 0.0002*** |
| | | Groups: F (1, 28) = 2.867 | P = 0.1015 | Target: hM4Di+Saline vs. hM4Di+CNO, P = 0.0034*** |
| **11E (OFT)** | Two-way ANOVA, post hoc Bonferroni’s test | Interaction: F (1, 28) = 0.9067 | P = 0.3491 | mCherry + Saline vs. hM4Di + Saline, P > 0.05 |

| | | | | |
|---|---|---|---|---|
| | | Treatment: F (1, 28) = 0.03542 | P = 0.8521 | mCherry + CNO vs. hM4Di+CNO, P > 0.05 |
| | | Groups: F (1, 28) = 0.1759 | P = 0.6781 | hM4Di + Saline vs. hM4Di + CNO, P > 0.05 |
| **11E (FST)** | Two-way ANOVA, post hoc Bonferroni's test | Interaction: F (1, 28) = 6.241 | P = 0.0186 | mCherry + Saline vs. hM4Di+Saline, P > 0.05 |
| | | Treatment: F (1, 28) = 8.402 | P = 0.0072 | mCherry + CNO vs. hM4Di + CNO, P = 0.0041** |
| | | Groups: F (1, 28) = 8.513 | P = 0.0069 | hM4Di+Saline vs. hM4Di+CNO, P = 0.004** |
| **11E (TST)** | Two-way ANOVA, post hoc Bonferroni's test | Interaction: F (1, 28) = 21.82 | P < 0.0001 | mCherry + Saline vs. hM4Di + Saline, P > 0.05 |
| | | Treatment: F (1, 28) = 12.29 | P = 0.0016 | mCherry + CNO vs. hM4Di +CNO, P < 0.0001*** |
| | | Groups: F (1, 28) = 15.90 | P = 0.0004 | hM4Di+Saline vs. hM4Di+CNO, P < 0.0001*** |
| **11E (SPT)** | Two-way ANOVA, post hoc Bonferroni's test | Interaction: F (1, 28) = 2.538 | P = 0.1223 | mCherry + Saline vs. hM4Di + Saline, P > 0.05 |
| | | Treatment: F (1, 28) = 13.38 | P = 0.0010 | mCherry + CNO vs. hM4Di+CNO, P = 0.0054** |
| | | Groups: F (1, 28) = 8.609 | P = 0.0066 | hM4Di+Saline vs. hM4Di+CNO, P = 0.0204* |
| **13D** | Two-way ANOVA, post hoc Bonferroni's test | Interaction: F (1, 28) = 0.06592 | P = 0.7992 | Target: Saline (PV+hM4Di) vs. Saline (SST+hM4Di), P > 0.05 |
| | | Treatment: F (1, 28) = 1.303 | P = 0.2634 | Target: CNO (PV+hM4Di) vs. CNO (SST+hM4Di), P > 0.05 |
| | | Groups: F (1, 28) = 0.1942 | P = 0.6628 | Target: Saline (SST+hM4Di) vs. CNO (SST+hM4Di), P > 0.05 |
| **13E (OFT)** | Two-way ANOVA, post hoc Bonferroni's test | Interaction: F (1, 28) = 0.05962 | P = 0.8089 | Target: Saline (PV+hM4Di) vs. Saline (SST+hM4Di), P > 0.05 |
| | | Treatment: F (1, 28) = | P = 0.4305 | Target: CNO (PV+hM4Di) vs. CNO (SST+hM4Di), P |

| | | | | |
|---|---|---|---|---|
| | | 0.6398 | | > 0.05 |
| | | Groups: F (1, 28) = 0.004254 | P = 0.9485 | Target: Saline (SST+hM4Di) vs. CNO (SST+hM4Di), P > 0.05 |
| **13E (FST)** | Two-way ANOVA, post hoc Bonferroni's test | Interaction: F (1, 28) = 4.283 | P = 0.0478 | Target: Saline (PV+hM4Di) vs. Saline (SST+hM4Di), P > 0.05 |
| | | Treatment: F (1, 28) = 0.01162 | P = 0.9149 | Target: CNO (PV+hM4Di) vs. CNO (SST+hM4Di), P > 0.05 |
| | | Groups: F (1, 28) = 0.000 | P > 0.9999 | Target: Saline (SST+hM4Di) vs. CNO (SST+hM4Di), P > 0.05 |
| **13E (TST)** | Two-way ANOVA, post hoc Bonferroni's test | Interaction: F (1, 28) = 1.397 | P = 0.2471 | Target: Saline (PV+hM4Di) vs. Saline (SST+hM4Di), P > 0.05 |
| | | Treatment: F (1, 28) = 0.4284 | P = 0.5181 | Target: CNO (PV+hM4Di) vs. CNO (SST+hM4Di), P > 0.05 |
| | | Groups: F (1, 28) = 9.543e-005 | P = 0.9923 | Target: Saline (SST+hM4Di) vs. CNO (SST+hM4Di), P > 0.05 |
| **13E (SPT)** | Two-way ANOVA, post hoc Bonferroni's test | Interaction: F (1, 28) = 0.1060 | P = 0.7471 | Target: Saline (PV+hM4Di) vs. Saline (SST+hM4Di), P > 0.05 |
| | | Treatment: F (1, 28) = 1.015 | P = 0.3224 | Target: CNO (PV+hM4Di) vs. CNO (SST+hM4Di), P > 0.05 |
| | | Groups: F (1, 28) = 0.04963 | P = 0.8253 | Target: Saline (SST+hM4Di) vs. CNO (SST+hM4Di), P > 0.05 |
| **14B** | Unpaired two-tailed Student's t-test | _ | _ | Con vs. Sus, P > 0.05 |
| **14E** | Two-way ANOVA, post hoc Bonferroni's test | Interaction: F (1.36) = 0.3748 | P = 0.5442 | Target: Vehicle vs. Propranolol, P > 0.05 |
| | | Treatment: F (1.36) = 0.03173 | P = 0.8596 | |
| | | Groups: F (1,36) = 0.04248 | P = 0.8379 | |

| | | | | |
|---|---|---|---|---|
| **14F (OFT)** | Unpaired two-tailed Student's t-test | _ | _ | Vehicle vs. Propranolol, $P > 0.05$ |
| **14F (FST)** | Unpaired two-tailed Student's t-test | _ | _ | Vehicle vs. Propranolol, $P > 0.05$ |
| **14F (TST)** | Unpaired two-tailed Student's t-test | _ | _ | Vehicle vs. Propranolol, $P > 0.05$ |
| **14F (SPT)** | Unpaired two-tailed Student's t-test | _ | _ | Vehicle vs. Propranolol, $P > 0.05$ |
| **14I** | Two-way ANOVA, post hoc Bonferroni's test | Interaction: $F_{(1, 28)} = 0.2048$<br>Treatment: $F_{(1, 28)} = 0.6179$<br>Groups: $F_{(1, 28)} = 0.1126$ | $P = 0.6544$<br>$P = 0.4384$<br>$P = 0.7397$ | Target: Vehicle vs. Phentolamine, $P > 0.05$ |
| **14J (OFT)** | Unpaired two-tailed Student's t-test | _ | _ | Vehicle vs. Phentolamine, $P > 0.05$ |
| **14J (FST)** | Unpaired two-tailed Student's t-test | _ | _ | Vehicle vs. Phentolamine, $P > 0.05$ |
| **14J (TST)** | Unpaired two-tailed Student's t-test | _ | _ | Vehicle vs. Phentolamine, $P > 0.05$ |
| **14J (SPT)** | Unpaired two-tailed Student's t-test | _ | _ | Vehicle vs. Phentolamine, $P > 0.05$ |
| **15D** | Unpaired two-tailed Student's t-test | _ | _ | FACS: Con vs. Sus, $P = 0.0012$** |
| **16C** | Unpaired two-tailed Student's t-test | _ | _ | IF LC: EGFP vs. Cre, $P < 0.0001$*** |
| **16D** | One-way ANOVA, post hoc Bonferroni's test | _ | _ | Protein LC: EGFP vs. Cre, $P = 0.0482$*<br>Protein dLS: EGFP vs. Cre, |

| | | | | |
|---|---|---|---|---|
| | | | | P = 0.0223* |
| **16F** | Two-way ANOVA, post hoc Bonferroni's test | Interaction: F (1, 44) = 3.661 | P = 0.0622 | Target: Pre SSDS+AAV-EGFP vs. Pre SSDS+AAV-Cre, P > 0.05 |
| | | Treatment: F (1, 44) = 9.061 | P = 0.0043 | Target: Pre SSDS+AAV-Cre vs. Post SSDS+AAV-Cre, P = 0.0068** |
| | | Groups: F (1, 44) = 11.46 | P = 0.0015 | Target: Post SSDS+AAV-EGFP vs. Post SSDS+AAV-Cre, P = 0.0031** |
| **16G (OFT)** | Unpaired two-tailed Student's t-test | _ | _ | AAV-EGFP vs. AAV-Cre, P > 0.05 |
| **16G (FST)** | Unpaired two-tailed Student's t-test | _ | _ | AAV-EGFP vs. AAV-Cre, P = 0.0002*** |
| **16G (TST)** | Unpaired two-tailed Student's t-test | _ | _ | AAV-EGFP vs. AAV-Cre, P = 0.0064** |
| **16G (SPT)** | Unpaired two-tailed Student's t-test | _ | _ | AAV-EGFP vs. AAV-Cre, P = 0.0002*** |
| **17A** | Unpaired two-tailed Student's t-test | _ | _ | Protein: Con-shRNA vs. BDNF-shRNA, P = 0.003** |
| **18E** | One-way repeated-measures ANOVA, Dunn's multiple comparisons test | _ | _ | Stimu vs. Base P < 0.0001*** |
| | | | | Base vs. Stimu P < 0.0001*** |
| **18F** | One-way repeated-measures ANOVA, Dunn's multiple comparisons test | _ | _ | Light off vs. Light on P < 0.0001*** |
| | | | | Light on vs. Light off P < 0.0001*** |

| | | | | |
|---|---|---|---|---|
| **18G** | Unpaired two-tailed Student's t-test | _ | _ | Saline vs. K252a, P = 0.0032** |
| **19C** | Two-way ANOVA, post hoc Bonferroni's test | Interaction: F (1, 32) = 0.1109 | P = 0.7413 | Target: Vehicle vs. K252a, P > 0.9999 |
| | | Treatment: F (1, 32) = 2.330 | P = 0.1367 | No Target: Con vs. Target: Con, P > 0.9999 |
| | | Groups: F (1, 32) = 1.356 | P = 0.2528 | No Target: K252a vs. Target: K252a, P > 0.9999 |
| **19D (OFT)** | Unpaired two-tailed Student's t-test | _ | _ | Vehicle vs. K252a, P > 0.05 |
| **19D (FST)** | Unpaired two-tailed Student's t-test | _ | _ | Vehicle vs. K252a, P > 0.05 |
| **19D (TST)** | Unpaired two-tailed Student's t-test | _ | _ | Vehicle vs. K252a, P > 0.05 |
| **19D (SPT)** | Unpaired two-tailed Student's t-test | _ | _ | Vehicle vs. K252a, P > 0.05 |
| **20C** | Two-way ANOVA, post hoc Bonferroni's test | Interaction: F (1, 36) = 3.744 | P=0.0609 | Target: Vehicle vs. BDNF-nAb, P = 0.0246* |
| | | Treatment: F (1, 36) = 5.767 | P=0.0216 | No Target: BDNF-nAb vs. Target: BDNF-nAb, P = 0.0246* |
| | | Groups: F (1, 36) = 5.767 | P=0.0216 | |
| **20D (OFT)** | Unpaired two-tailed Student's t-test | _ | _ | Vehicle vs. BDNF-nAb, P > 0.05 |
| **20D (FST)** | Unpaired two-tailed Student's t-test | _ | _ | Vehicle vs. BDNF-nAb, P = 0.0028** |
| **20D (TST)** | Unpaired two-tailed Student's t-test | _ | _ | Vehicle vs. BDNF-nAb, P = 0.0023** |
| **20D (SPT)** | Unpaired two-tailed Student's t-test | _ | _ | Vehicle vs. BDNF-nAb, P = 0.0157* |
| **21C** | Two-way | Interaction: F | P = 0.0592 | Target: Saline vs. BDNF, P |

| | | | | |
|---|---|---|---|---|
| | ANOVA, post hoc Bonferroni's test | (1, 36) = 3.796 | | = 0.0191* |
| | | Treatment: F (1, 36) = 10.92 | P = 0.0022 | No target: Saline vs. Target: saline, P = 0.0041** |
| | | Groups: F (1, 36) = 6.363 | P = 0.0162 | No target: BDNF vs. Target: BDNF, P > 0.9999 |
| **21D (OFT)** | Unpaired two-tailed Student's t-test | _ | _ | Saline vs. BDNF, P > 0.05 |
| **21D (FST)** | Unpaired two-tailed Student's t-test | _ | _ | Saline vs. BDNF, P = 0.0159* |
| **21D (TST)** | Unpaired two-tailed Student's t-test | _ | _ | Saline vs. BDNF, P = 0.0010** |
| **21D (SPT)** | Unpaired two-tailed Student's t-test | _ | _ | Saline vs. BDNF, P = 0.0133* |
| **22B** | Unpaired two-tailed Student's t-test | _ | _ | Body weight: Con vs. cKO, P > 0.05 |
| **22D** | Unpaired two-tailed Student's t-test | _ | _ | TH neurons: Con vs. cKO, P > 0.05 |
| **23C** | Two-way ANOVA, post hoc Bonferroni's test | Interaction: F (1, 28) = 4.540 | P = 0.0420 | Target: Con-shRNA: Saline vs. Con-shRNA: *R*-ketamine, P = 0.0238* |
| | | Treatment: F (1, 28) = 3.775 | P = 0.0621 | Target: Con-shRNA: *R*-ketamine vs. BDNF-shRNA: *R*-ketamine, P = 0.0452* |
| | | Groups: F (1, 28) = 5.332 | P = 0.0285 | |
| **23D (OFT)** | Two-way ANOVA, post hoc Bonferroni's test | Interaction: F (1, 28) = 0.4583 | P = 0.5040 | Saline: Con-shRNA vs. *R*-ketamine: Con-shRNA, P > 0.9999 |
| | | Treatment: F (1, 28) = 0.02127 | P = 0.8851 | *R*-ketamine: con-shRNA vs. R-ketamine: BDNF-shRNA, P > 0.9999 |
| | | Groups: F (1, | P = 0.6817 | |

| | | | | |
|---|---|---|---|---|
| | | 28) = 0.1717 | | |
| **23D (FST)** | Two-way ANOVA, post hoc Bonferroni's test | Interaction: F (1, 28) = 9.436 | P = 0.0047 | Saline: Con-shRNA vs. *R*-ketamine: Con-shRNA, P = 0.0005*** |
| | | Treatment: F (1, 28) = 11.71 | P = 0.0019 | *R*-ketamine: Con-shRNA vs. *R*-ketamine: BDNF-shRNA, P = 0.0104* |
| | | Groups: F (1, 28) = 3.336 | P = 0.0785 | |
| **23D (TST)** | Two-way ANOVA, post hoc Bonferroni's test | Interaction: F (1, 28) = 7.521 | P = 0.0105 | Saline: Con-shRNA vs. *R*-ketamine: Con-shRNA, P = 0.0224* |
| | | Treatment: F (1, 28) = 3.001 | P = 0.0942 | *R*-ketamine: Con-shRNA vs. *R*-ketamine: BDNF-shRNA, P = 0.0165* |
| | | Groups: F (1, 28) = 3.613 | P = 0.0677 | |
| **23D (SPT)** | Two-way ANOVA, post hoc Bonferroni's test | Interaction: F (1, 28) = 6.756 | P = 0.0147 | Saline: Con-shRNA vs. *R*-ketamine: Con-shRNA, P = 0.0036** |
| | | Treatment: F (1, 28) = 8.259 | P = 0.0077 | *R*-ketamine: Con-shRNA vs. *R*-ketamine: BDNF-shRNA, P = 0.0138* |
| | | Groups: F (1, 28) = 4.604 | P = 0.0407 | |
| **23G** | Two-way ANOVA, post hoc Bonferroni's test | Interaction: F (1, 28) = 4.765 | P = 0.0376 | Target: Vehicle: Saline vs. Vehicle: *R*-ketamine, P = 0.0307* |
| | | Treatment: F (1, 28) = 3.894 | P = 0.0584 | Target: Vehicle: *R*-ketamine vs. BDNF-nAb: *R*-ketamine, P = 0.0392* |
| | | Groups: F (1, 28) = 4.461 | P = 0.0437 | |
| **23H (OFT)** | Two-way ANOVA, post | Interaction: F (1, 28) = | P = 0.5263 | Saline: Vehicle vs. *R*-ketamine: Vehicle, P > |

| | | | | |
|---|---|---|---|---|
| | hoc Bonferroni's test | 0.4117 | | 0.05 |
| | | Treatment: F (1, 28) = 0.2758 | P = 0.6036 | *R*-ketamine: Vehicle vs. *R*-ketamine: BDNF-nAb, P > 0.05 |
| | | Groups: F (1, 28) = 0.03023 | P = 0.8632 | |
| **23H (FST)** | Two-way ANOVA, post hoc Bonferroni's test | Interaction: F (1, 28) = 7.808 | P = 0.0093 | Saline: Vehicle vs. R-ketamine: Vehicle, P = 0.0080** |
| | | Treatment: F (1, 28) = 5.040 | P = 0.0328 | *R*-ketamine: Vehicle vs. R-ketamine: BDNF-nAb, P = 0.0082** |
| | | Groups: F (1, 28) = 4.979 | P = 0.0338 | |
| **23H (TST)** | Two-way ANOVA, post hoc Bonferroni's test | Interaction: F (1, 28) = 6.932 | P = 0.0136 | Saline: Vehicle vs. R-ketamine: Vehicle, P = 0.0019** |
| | | Treatment: F (1, 28) = 9.994 | P = 0.0038 | R-ketamine: Vehicle vs. R-ketamine: BDNF-nAb, P = 0.0018** |
| | | Groups: F (1, 28) = 10.23 | P = 0.0034 | |
| **23H (SPT)** | Two-way ANOVA, post hoc Bonferroni's test | Interaction: F (1, 28) = 4.952 | P = 0.0343 | Saline: Vehicle vs. R-ketamine: Vehicle, P = 0.0164* |
| | | Treatment: F (1, 28) = 5.876 | P = 0.0221 | R-ketamine: Vehicle vs. R-ketamine: BDNF-nAb, P = 0.0121* |
| | | Groups: F (1, 28) = 6.709 | P = 0.0151 | |
| **24A (SIR)** | One-way ANOVA, post hoc Tukey's test | F (2, 77) = 31.93 | P < 0.0001 | Con vs. Sus, P = 0.0004*** |
| | | | | Sus vs. Res, P < 0.0001*** |
| **24A (SIT)** | Two-way ANOVA, post hoc Bonferroni's test | Interaction: F (2, 154) = 19.91 | P < 0.0001 | Con (No target vs. Target), P > 0.9999 |
| | | Treatment: F | P = 0.0002 | Sus (No target vs. Target), P |

| | | | | |
|---|---|---|---|---|
| | | (2, 154) = 9.225 | | < 0.0001*** |
| | | Groups: F (1, 154) = 0.5948 | P = 0.4417 | Res (No target vs. Target), P = 0.0109* |
| **24B (SIR)** | Unpaired two-tailed Student's t-test | _ | _ | Sus vs. Res, P < 0.0001*** |
| **24B (SIT)** | Two-way ANOVA, post hoc Bonferroni's test | Interaction: F (1, 100) = 52.11 | P < 0.0001 | Sus (No target vs. Target), P < 0.0001*** |
| | | Treatment: F (1, 100) = 14.85 | P = 0.0002 | Res (No target vs. Target), P = 0.0022** |
| | | Groups: F (1, 100) = 2.056 | P = 0.1547 | |
| **24C (SIR)** | Unpaired two-tailed Student's t-test | _ | _ | Sus vs. Res, P < 0.0001*** |
| **24C (SIT)** | Two-way ANOVA, post hoc Bonferroni's test | Interaction: F (1, 128) = 48.37 | P < 0.0001 | Sus (No target vs. Target), P <0.0001 *** |
| | | Treatment: F (1, 128) = 8.280 | P = 0.0047 | Res (No target vs. Target), P = 0.0103* |
| | | Groups: F (1, 128) = 2.234 | P = 0.1374 | |
| **24D (SIR)** | Unpaired two-tailed Student's t-test | _ | _ | Con-shRNA vs. BDNF-shRNA, P > 0.05 |
| **24D (SIT)** | Two-way ANOVA, post hoc Bonferroni's test | Interaction: F (1, 36) = 0.3361 | P = 0.5657 | Sus (No target vs. Target), P > 0.9999 |
| | | Treatment: F (1, 36) = 0.3660 | P = 0.5490 | Res (No target vs. Target), P > 0.9999 |
| | | Groups: F (1, 36) = 1.892 | P = 0.1775 | |
| **24E (SIR)** | Unpaired two-tailed Student's t-test | _ | _ | Sus vs. Res, P < 0.0001*** |

| | | | | |
|---|---|---|---|---|
| **24E (SIT)** | Two-way ANOVA, post hoc Bonferroni's test | Interaction: F (1, 60) = 27.84 | P < 0.0001 | Sus (No target vs. Target), P = 0.0001*** |
| | | Treatment: F (1, 60) = 8.678 | P = 0.0046 | Res (No target vs. Target), P = 0.0203* |
| | | Groups: F (1, 60) = 0.2015 | P = 0.6551 | |
| **24F (SIR)** | Unpaired two-tailed Student's t-test | _ | _ | Saline vs. BDNF, P > 0.05 |
| **24F (SIT)** | Two-way ANOVA, post hoc Bonferroni's test | Interaction: F (1, 40) = 1.344 | P = 0.2533 | Saline (No target vs. Target), P = 0.0014** |
| | | Treatment: F (1, 40) = 1.893 | P = 0.1765 | BDNF (No target vs. Target), P < 0.0001*** |
| | | Groups: F (1, 40) = 47.42 | P < 0.0001 | |
| **24G (SIR)** | Unpaired two-tailed Student's t-test | _ | _ | Sus vs. Res, P < 0.0001*** |
| **24G (SIT)** | Two-way ANOVA, post hoc Bonferroni's test | Interaction: F (1, 52) = 23.90 | P < 0.0001 | Sus (No target vs. Target), P < 0.0001*** |
| | | Treatment: F (1, 52) = 9.832 | P = 0.0028 | Res (No target vs. Target), P < 0.0001*** |
| | | Groups: F (1, 52) = 1.591 | P = 0.2128 | |
| **24H (SIR)** | Unpaired two-tailed Student's t-test | _ | _ | Sus vs. Res, P < 0.0001*** |
| **24H (SIT)** | Two-way ANOVA, post hoc Bonferroni's test | Interaction: F (1, 54) = 6.993 | P = 0.0107 | Sus (No target vs. Target), P < 0.0001*** |
| | | Treatment: F (1, 54) = 0.6691 | P = 0.4170 | Res (No target vs. Target), P > 0.9999 |
| | | Groups: F (1, 54) = 0.3052 | P = 0.5829 | |
| **24I (SIR)** | Unpaired | _ | _ | Sus vs. Res, P < 0.0001*** |

| | | | | |
|---|---|---|---|---|
| | two-tailed Student's t-test | | | |
| **24I (SIT)** | Two-way ANOVA, post hoc Bonferroni's test | Interaction: F (1, 108) = 41.73 | P < 0.0001 | Sus (No target vs. Target), P < 0.0001*** |
| | | Treatment: F (1, 108) = 10.01 | P = 0.0020 | Res (No target vs. Target), P = 0.0046** |
| | | Groups: F (1, 108) = 0.8235 | P = 0.3662 | |
| **24J (SIR)** | Unpaired two-tailed Student's t-test | _ | _ | Sus vs. Res, P < 0.0001*** |
| **24J (SIT)** | Two-way ANOVA, post hoc Bonferroni's test | Interaction: F (1, 96) = 41.77 | P < 0.0001 | Sus (No target vs. Target), P < 0.0001*** |
| | | Treatment: F (1, 96) = 9.015 | P = 0.0034 | Res (No target vs. Target), P = 0.0080** |
| | | Groups: F (1, 96) = 1.517 | P = 0.2211 | |
| **25A** | Two-way ANOVA, post hoc Bonferroni's test | Interaction: F (2, 54) = 0.01097 | P = 0.9891 | Con-Gq: Saline vs. Sus-Gq: Saline, P < 0.0001*** |
| | | Treatment: F (2, 54) = 40.87 | P < 0.0001 | Con-Gq: CNO vs. Sus-Gq: CNO, P < 0.0001*** |
| | | Groups: F (1, 54) = 0.2178 | P = 0.6426 | |
| **25B** | Two-way ANOVA, post hoc Bonferroni's test | Interaction: F (1, 28) = 0.6596 | P = 0.4236 | mCherry: Saline vs. Gq: Saline, P > 0.9999 |
| | | Treatment: F (1, 28) = 0.1311 | P = 0.7200 | mCherry: CNO vs. Gq: CNO, P > 0.9999 |
| | | Groups: F (1, 28) = 0.4827 | P = 0.4929 | Gq: Saline vs. Gq: CNO, P > 0.9999 |
| **25C** | Two-way ANOVA, post hoc Bonferroni's test | Interaction: F (2, 36) = 0.3035 | P = 0.7401 | mCherry: No light vs. mCherry: Light, P > 0.9999 |

| | | | | |
|---|---|---|---|---|
| | | Treatment: F (2, 36) = 0.4724 | P = 0.6273 | mCherry: Light vs. PV + Gi: Light, P > 0.9999 |
| | | Groups: F (1, 36) = 0.6261 | P = 0.4340 | mCherry: Light vs. SST + Gi: Light, P > 0.9999 |
| **25D** | Unpaired two-tailed Student's t-test | _ | _ | Con-shRNA vs. BDNF-shRNA, P < 0.0001*** |
| **25E** | Unpaired two-tailed Student's t-test | _ | _ | K252a vs. Vehicle, P > 0.05 |
| **25F** | Unpaired two-tailed Student's t-test | _ | _ | BDNF vs. Saline, P > 0.05 |
| **25G** | Two-way ANOVA, post hoc Bonferroni's test | Interaction: F (1, 40) = 0.4260 | P = 0.5177 | TH-Cre: AAV-Con vs. BDNF-cKO: AAV-Con, P > 0.9999 |
| | | Treatment: F (1, 40) = 0.4800 | P = 0.4924 | TH-Cre: AAV-Con vs. TH-Cre: AAV-BDNF, P > 0.9999 |
| | | Groups: F (1, 40) = 0.7631 | P = 0.3876 | BDNF-cKO: AAV-Con vs. BDNF-cKO: AAV-BDNF, P > 0.9999 |
| **25H** | Two-way ANOVA, post hoc Bonferroni's test | Interaction: F (1, 34) = 0.7922 | P = 0.3797 | Con-shRNA: Saline vs. Con-shRNA: *S*-Ketamine, P > 0.9999 |
| | | Treatment: F (1, 34) = 0.9923 | P = 0.3262 | Con-shRNA: *S*-Ketamine vs. BDNF-shRNA:*S*-Ketamine, P > 0.9999 |
| | | Groups: F (1, 34) = 0.02724 | P = 0.8699 | |
| **25I** | Two-way ANOVA, post hoc Bonferroni's test | Interaction: F (1, 28) = 0.2655 | P = 0.6104 | Con-shRNA: Saline vs. Con-shRNA: Fluoxetine, P > 0.9999 |
| | | Treatment: F (1, 28) = 0.01738 | P = 0.8961 | Con-shRNA: Fluoxetine, vs. BDNF-shRNA: Fluoxetine, P > 0.9999 |
| | | Groups: F (1, 28) = 0.001292 | P = 0.9716 | |

| | | | | |
|---|---|---|---|---|
| **25J** | One-way ANOVA, post hoc Bonferroni's test | F (2, 33) = 23.63 | P < 0.0001 | Con vs. Sus, P < 0.0001*** |
| | | | | Sus vs. Res, P < 0.0001*** |
| **25K** | Two-way ANOVA, post hoc Bonferroni's test | Interaction: F (1, 28) = 1.208 | P = 0.2810 | Con + mCherry: Saline vs. Con + mCherry: CNO, P > 0.9999 |
| | | Treatment: F (1, 28) = 0.2595 | P = 0.6145 | Con+ Gq: Saline vs. Con+ Gq: CNO, P > 0.9999 |
| | | Groups: F (1, 28) = 0.2595 | P = 0.6145 | Con + mCherry: CNO vs. Con + Gq: CNO, P > 0.9999 |
| **25L** | Unpaired two-tailed Student's t-test | _ | _ | Gq + Saline vs. Gq + CNO, P > 0.05 |
| **25M** | Two-way ANOVA, post hoc Bonferroni's test | Interaction: F (1, 36) = 0.8569 | P = 0.3608 | mCherry: Saline vs. Gq: Saline, P > 0.9999 |
| | | Treatment: F (1, 36) = 0.008381 | P = 0.9276 | mCherry: CNO vs. Gq: CNO, P > 0.9999 |
| | | Groups: F (1, 36) = 0.0001035 | P = 0.9919 | Gq: Saline vs. Gq: CNO, P > 0.9999 |
| **25N** | Unpaired two-tailed Student's t-test | _ | _ | mCherry vs. Gq, P = 0.0001*** |
| **25O** | Unpaired two-tailed Student's t-test | _ | _ | mCherry vs. Gq, P = 0.0003*** |
| **S25P** | Two-way ANOVA, post hoc Bonferroni's test | Interaction: F (1, 28) = 8.076 | P = 0.0083 | mCherry: Saline vs. Gi: Saline, P > 0.9999 |
| | | Treatment: F (1, 28) = 14.48 | P = 0.0007 | mCherry: CNO vs. Gi: CNO, P = 0.0004*** |
| | | Groups: F (1, 28) = 6.400 | P = 0.0173 | Gi: Saline vs. Gi: CNO, P = 0.0043** |
| **S25Q** | Two-way | Interaction: F | P = 0.9338 | PV+ Gi: Saline vs. SST+ |

| | | | | |
|---|---|---|---|---|
| | ANOVA, post hoc Bonferroni's test | (1, 28) = 0.007033 | | Gi: Saline, P > 0.9999 |
| | | Treatment: F (1, 28) = 0.01128 | P = 0.9162 | PV+ Gi: CNO vs. SST+ Gi: CNO, P > 0.9999 |
| | | Groups: F (1, 28) = 1.164 | P = 0.2898 | SST+ Gi: Saline vs. SST+ Gi: CNO, P > 0.9999 |
| **S25R** | Unpaired two-tailed Student's t-test | _ | _ | Vehicle vs. Propranolol, P > 0.05 |
| **S25S** | Unpaired two-tailed Student's t-test | _ | _ | Vehicle vs. Phentolamine, P > 0.05 |
| **S25T** | Unpaired two-tailed Student's t-test | _ | _ | AAV-EGFP vs. AAV-Cre, P = 0.0020** |
| **S25U** | Unpaired two-tailed Student's t-test | _ | _ | Vehicle vs. K252a, P > 0.05 |
| **S25V** | Unpaired two-tailed Student's t-test | _ | _ | Vehicle vs. BDNF-nAb, P = 0.0001*** |
| **S25W** | Unpaired two-tailed Student's t-test | _ | _ | Saline vs. BDNF, P > 0.05 |
| **S25X** | Two-way ANOVA, post hoc Bonferroni's test | Interaction: F (1, 28) = 6.369 | P = 0.0176 | Saline: Con-shRNA vs. R-Ketamine: Con-shRNA, P = 0.0081** |
| | | Treatment: F (1, 28) = 6.294 | P = 0.0182 | R-Ketamine: Con-shRNA vs. R-Ketamine: BDNF-shRNA, P = 0.0011** |
| | | Groups: F (1, 28) = 12.80 | P = 0.0013 | |
| **S25Y** | Two-way ANOVA, post hoc Bonferroni's test | Interaction: F (1, 28) = 7.762 | P = 0.0095 | Saline: Vehicle vs. R-Ketamine: Vehicle, P = 0.0005*** |
| | | Treatment: F (1, 28) = 13.66 | P = 0.0009 | R-Ketamine: Vehicle vs. R-Ketamine: BDNF-nAb, P = 0.0076** |

| | | |
|---|---|---|
| | Groups: $F_{(1, 28)} = 5.209$ | $P = 0.0303$ |
